# Brain Permeable SGK1 Inhibitors: A Promising Therapeutic Strategy for Neurodegenerative Diseases

**DOI:** 10.1021/acs.jmedchem.5c03050

**Published:** 2026-03-16

**Authors:** Enrique Madruga, Alfonso Garcia-Rubia, Carlos Sanchez-Nuñez, Loreto Martinez-Gonzalez, Ana María Fernandez-Escamilla, Isabel Lastres-Becker, Carmen Gil, Ana Martinez

**Affiliations:** † 54446 Centro de Investigaciones Biológicas “Margarita Salas”—CSIC, Ramiro de Maeztu 9, 28040 Madrid, Spain; ‡ Centro de Investigación Biomédica en Red en Enfermedades Neurodegeneartivas (CIBERNED), Instituto de Salud Carlos III, 28029 Madrid, Spain; § Instituto de Investigación, Desarrollo e Innovación en Biotecnología Sanitaria de Elche (IDiBE), 213118 Universitas Miguel Hernández, 03202 Elche, Alicante, Spain; ∥ Instituto de Investigaciones Biomédica “Sols-Morreale”—CSIC/UAM, Arturo Duperier 4, 28039 Madrid, Spain; ⊥ Instituto de Investigación Sanitaria La Paz (IdiPaz), Paseo de la Castellana, 261, 28046 Madrid, Spain

## Abstract

A major challenge in modern medicine is developing new therapies for aging-related diseases such as neurodegenerative disorders, whose prevalence increases with longer life expectancy. Although kinase inhibitors have achieved clinical success, their development for central nervous system (CNS) disorders remains limited due to the complexity of kinase networks and poor blood–brain barrier (BBB) permeability. Serum/glucocorticoid-regulated kinase 1 (SGK1) participates in multiple signaling pathways but remains an underexplored target in neurodegeneration. Following a mixed ligand- and structure-based virtual screening, we have previously identified a brain-penetrant SGK1 inhibitor. A medicinal chemistry program based on hit expansion and optimization for BBB permeability reported here has generated a new family of SGK1 inhibitors as chemical probes that enable the investigation of SGK1’s role in neurological disorders and serve as promising starting points for drug development. These findings highlight SGK1 as a potential therapeutic target for neurodegenerative diseases, such as Alzheimer’s disease.

## Graphical abstract

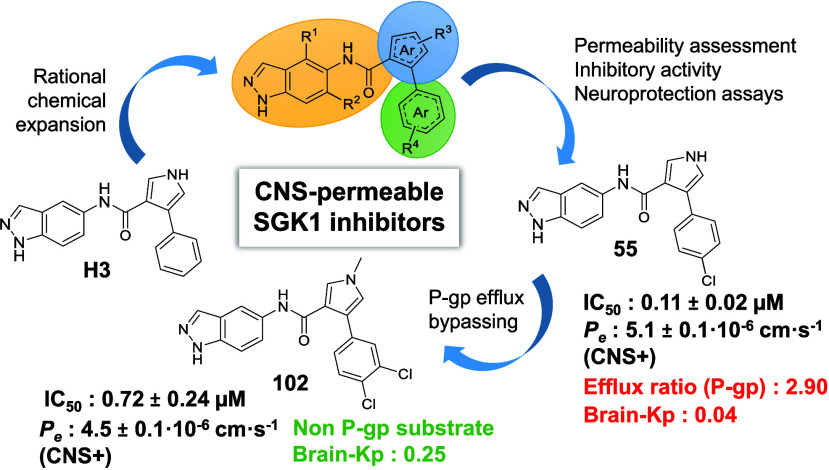

## Introduction

Neurodegeneration is defined as the progressive loss of neuronal structure and function, which ultimately results in cell death. This pathological process underlies a range of debilitating disorders, including Alzheimer’s disease (AD), Parkinson’s disease (PD), amyotrophic lateral sclerosis (ALS), and others.^1^ As global life expectancy rises, the prevalence of neurodegenerative diseases is increasing, posing a major challenge to public health systems. These conditions severely impact the quality of life of affected individuals and the sustainability of current healthcare systems.^2^ Consequently, the pursuit of novel therapeutic interventions has emerged as a primary concern in contemporary society. In the search for effective pharmacological targets, protein kinases have been the focus of repeated exploration over the last few decades, being considered as one of the most significant drug targets in the 21st century.^3^ Historically, kinases have been regarded as promising targets in the domain of oncology research. This assertion is substantiated by the fact that of the 85 FDA-approved drugs are classified as kinase inhibitors, 75 are designated for the treatment of several types of cancer.^3^ However, in other fields, such as neurodegenerative diseases, the development of kinase-based therapies remains less advanced. This is largely due to several challenges, including the complexity of kinase networks, limited blood-brain barrier (BBB) permeability, and the lack of robust biomarkers. Nevertheless, this area represents a promising frontier for expansion in the coming years.^4^

Serum/glucocorticoid regulated kinase 1 (SGK1), a ubiquitous serine/threonine kinase, regulates numerous signaling pathways being associated with various human diseases.^5^ Its pharmacological inhibition has been demonstrated to be therapeutic in the field of oncology,^6^ as well as in other areas such as diabetes,^7^ cardiovascular diseases,^8^ and inflammatory diseases.^9^ Despite extensive research on the complex relationship between SGK1 and the nervous system, its role in neurodegenerative disorders remains unclear. SGK1 is known to participate in molecular mechanisms including autophagy, oxidative stress, neuroinflammation, and TAU protein phosphorylation, processes that are central to pathologies such as AD and PD.^10^ However, controversy persists regarding its dual role, as SGK1 activity also appears to be essential for neuronal function.^11^

Two main factors contribute to this debate. The first is the limited characterization of SGK1 within neurodegenerative contexts. The second is the absence of selective, brain-permeable SGK1 inhibitors.^12^ Although several inhibitor families have been reported (Figure 1A), their ability to cross the BBB remains poor or unverified, rendering SGK1 an underexplored pharmacological target in neurodegeneration. In this regard, our research group has recently identified through a mixed ligand- and structure-based virtual screening using the European Chemical Biology Library (ECBL)^13^ as a source of chemical entities a novel SGK1 inhibitor, designated **H3** (Figure 1B). This inhibitor demonstrated neuroprotective potential *in vitro* and exhibited adequate effective permeability values, suggesting its potential to reach the central nervous system (CNS).^14^

**Figure 1:**
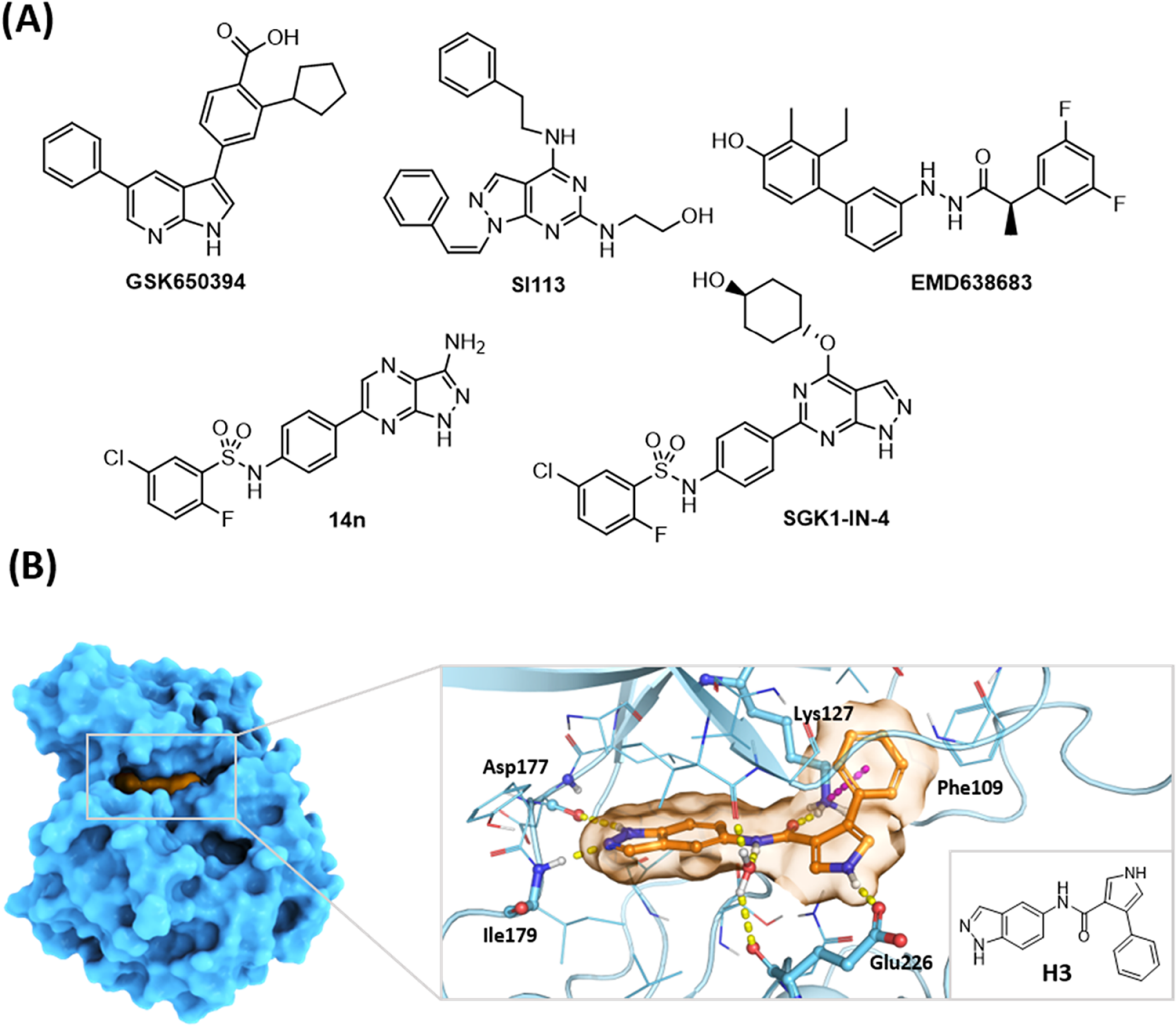
(A) Chemical structures of some known SGK1 inhibitors. **GSK650394**,^15^ **SI113**,^16^ **EMD638683**,^17^ **14n** ^18^, and **SGK1-IN-4**.^19^ (B) Chemical structure of **H3** and its predicted binding mode in complex with SGK1 (PDB: 3HDM).^14^ Right: **H3** positioned within the active site of the kinase. Key interacting amino acids are highlighted as sticks. Yellow, hydrogen bond interaction; purple, π–cation interaction.

This work is focused on the hit-to-lead optimization of the initial SGK1 inhibitor **H3**, the study of permeability across the BBB, and the evaluation of the neuroprotective potential of the most promising inhibitors in cellular models of AD. Furthermore, the investigation of the pharmacokinetic characteristics *in vivo* of these new SGK1 inhibitors prompted a subsequent cycle of medicinal chemistry improvement, providing compound **112**, to study the involvement of SGK1 in neurological disorders and/or to be optimized as drug candidate.

## Results and Discussion

### Hit-to-Lead Optimization of a Novel Family of SGK1 Inhibitors

Given the lack of SGK1 inhibitors capable of exerting therapeutic action within the CNS and our recent discovery of a novel BBB-permeable SGK1 inhibitor **H3**,^14^ we initiated a medicinal chemistry program aimed at improving the potency of **H3** while retaining BBB permeability. *In silico* binding mode analysis suggested that **H3** acts as a type I kinase inhibitor (Figure 1). Based on this model, the molecule was divided into three parts for chemical optimization: (i) the indazole fragment as a privileged scaffold able to establish two hydrogen bond interactions with the hinge region, (ii) the pyrrole ring, which exhibits hydrogen bond-type interactions with residues exposed to the solvent, and (iii) the phenyl ring, embedded within a hydrophobic pocket of the active site (Figure 2). Several structural modifications were proposed in order to study the different steric, electrostatic, and H-bonding properties of the various compounds when bound to SGK1.

**Figure 2:**
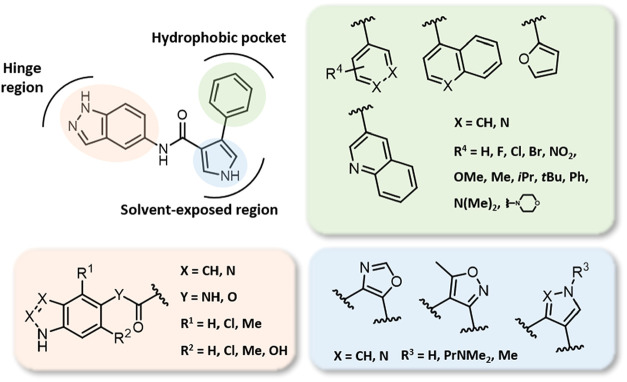
Proposed hit-to-lead optimization process.

Highlighting the importance of the indazole ring in forming a critical double hydrogen bond, analogues featuring alternative scaffolds such as indole and benzimidazole were designed and synthesized, along with a derivative in which the amide bond was replaced with an ester to elucidate the role of that polar hydrogen in SGK1 inhibition. Following the synthetic route for **H3** (Scheme 1), the cinnamic acid reacted with trimethylsilyl chloride (TMSCl) that, in the presence of MeOH, gives rise to methyl ester **1**.^20^ The cinnamic ester was converted to methyl 4-phenyl-1*H*-pyrrole-3-carboxylate **2**, using toluenesulfonylmethyl isocyanide (TosMIC) and NaH as a strong base, also known as Van Leusen synthesis.^21^ Subsequently, intermediate **2** was subjected to basic conditions in 1:1 solution of MeOH and H_2_O, heating under reflux to give the corresponding acid **3**. Finally, acid **3** was used to obtain the final products **H3**, **4** and **5** using 1-ethyl-3-(3-(dimethylamino)­propyl)­carbodiimide (EDC) as an activator of the acid group, *N*,*N*-diisopropylethylamine (DIPEA) as a base and 1-hydroxybenzotriazole (HOBt) as reaction catalyst,^22^ obtaining low yields for this last step (12, 17 and 5%, respectively). In the case of carboxylate **6**, 1,1′-carbonyldiimidazole (CDI) as coupling agent in the presence of 4-dimethylaminopyridine (DMAP) was employed,^23^ resulting also in poor yields (3%).

**Scheme 1:**
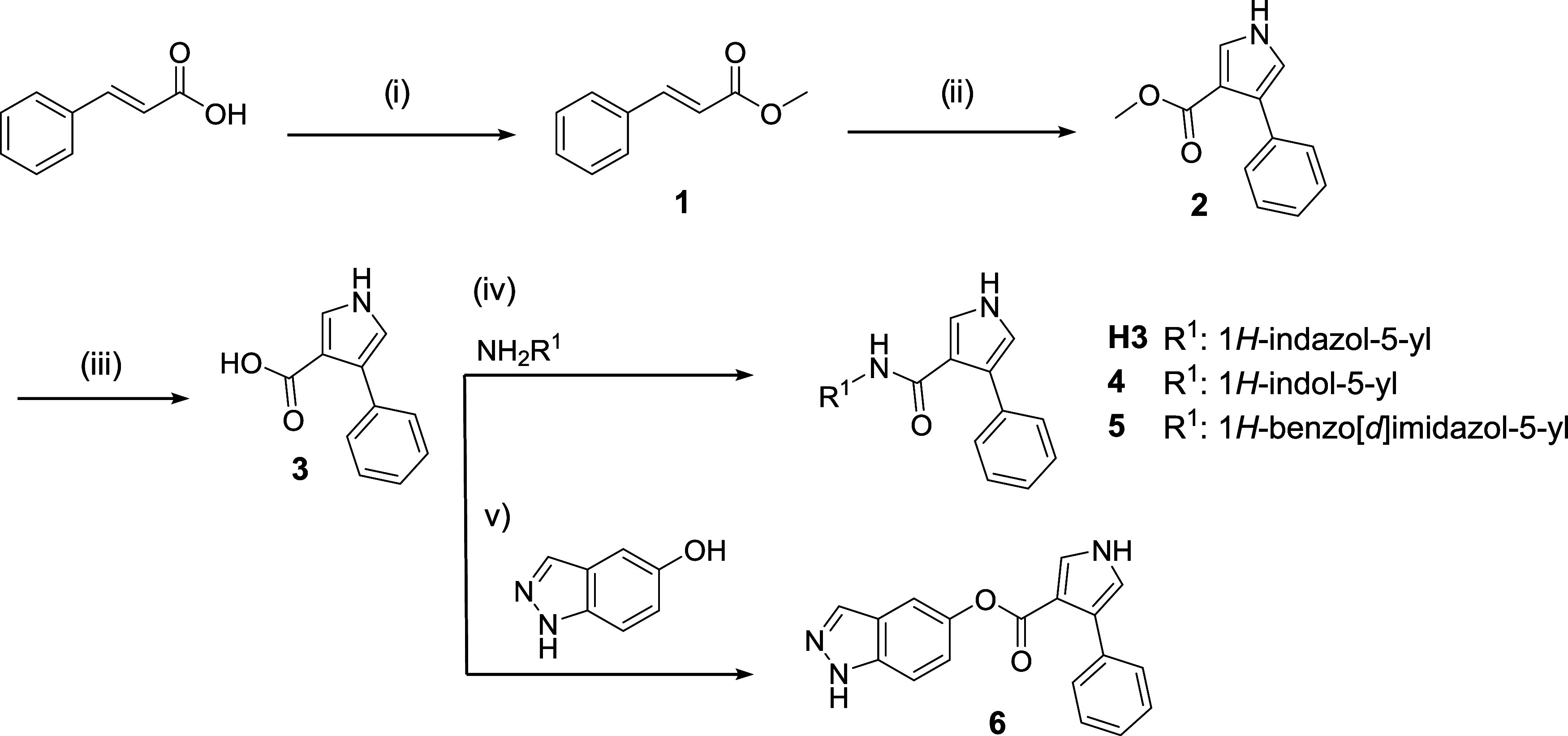
Initial Synthesis of **H3** and Derivatives^a^

a (i) TMSCl, MeOH, r.t., 24 h; (ii) TosMIC, NaH, DMF, 0 °C to r.t., 1 h; (iii) NaOH, MeOH, H_2_O, reflux, 2 h; (iv) EDC, HOBt, DMAP, DIPEA, CH_3_CN, r.t., 2–24 h; (v) CDI, DMAP, CH_3_CN, r.t., 48 h.

After that, a series of structural modifications to the phenyl ring attached to the pyrrole were proposed. This time, in order to increase the overall yield, a new synthetic route was defined: a first reaction where amidation occurs from the corresponding 3-arylacrylic acid and, subsequently, the formation of pyrrole in the same conditions as described before (Scheme 2). For the amidation reaction, hexafluorophosphate benzotriazole tetramethyl uronium (HBTU) was used as coupling agent, which gave rise to acrylamide derivatives **7–10**. In addition, to test whether or not the NH of indazole could interfere with the amide formation, the protected *tert*-butyl 5-amino-1*H*-indazole-1-carboxylate derivative was used in the latter two cases (**9**, **10**). Regardless of the presence of the protective group, higher yields were achieved than with the previous methodology. Then, *N*-(1*H*-indazole-5-yl)­arylacrylamide derivatives **7–10** were used in the next step to form the final products **H3**, **11–13**. Under these reaction conditions, the protecting group was unstable for **12** and **13**, obtaining yields similar to compounds **H3** and **11** (7, 3, 10, and 7%, respectively), which drastically decreases the overall yield of the route.

**Scheme 2:**
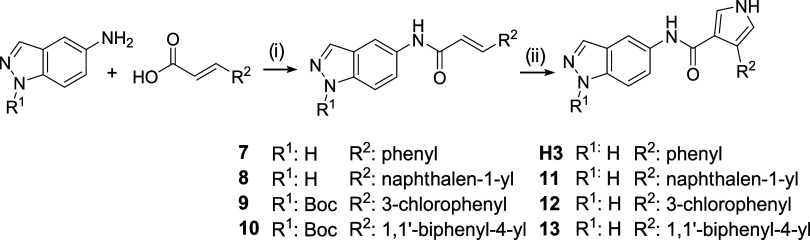
Alternative Synthesis of the **H3** Derivatives^a^

a (i) HBTU, DIPEA, DMF, r.t., 2 h; (ii) TosMIC, NaH, 0 °C to r.t., 2 h. Boc: *tert*-butoxycarbonyl.

Due to these results, we proceeded to use the original methodology but varying the conditions of the amide bond formation. It was found that the use of benzotriazole-1-yl-oxy-tris­(dimethylamino)­phosphonium hexafluorophosphate (BOP) as coupling agent allowed the synthesis of a collection of final products with higher yields in this last synthesis step (15–76%) (Scheme 3).^24^ It is noteworthy that the initial compound **H3** reached a 42% yield in this final step following this methodology, a considerable improvement over the initial 12%.

**Scheme 3:**
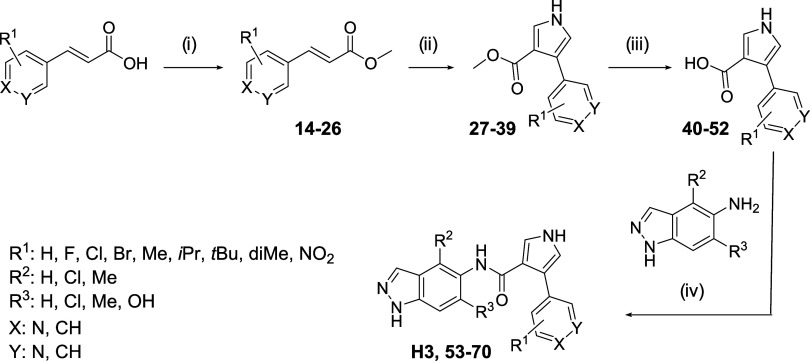
Optimized Synthesis of the **H3** Derivatives^a^

a (i) TMSCl, MeOH, r.t., 24 h; (ii) TosMIC, NaH, DMF, 0 °C to r.t., 1 to 2 h; (iii) NaOH, MeOH, H_2_O, reflux, 1 to 2 h; (iv) BOP, DIPEA, THF, r.t., 2 to 24 h.

According to Scheme 3, different analogues with electron-donating substituents that increase the electron density in the phenyl ring were proposed. However, as described in the literature, pyrrole intermediates could not be obtained.^25^ To solve this, a new synthetic route was employed. The corresponding aromatic aldehyde was subjected to a pyrrolidine-catalyzed Knoevenagel condensation with a 1,3-dicarbonyl compound to afford the corresponding conjugated alkene.^26,27^ Subsequent reaction with TosMIC and ester deprotection then led to the formation of acid-type intermediates, not only with electron-donating substituents (**71**-**73**), but also others such as quinoline derivatives **74** and **75** (Scheme 4). In the last step, the same conditions to coupling this carboxylic acids with the 5-aminoindazol were used, yielding compounds **76**-**80**.

**Scheme 4:**
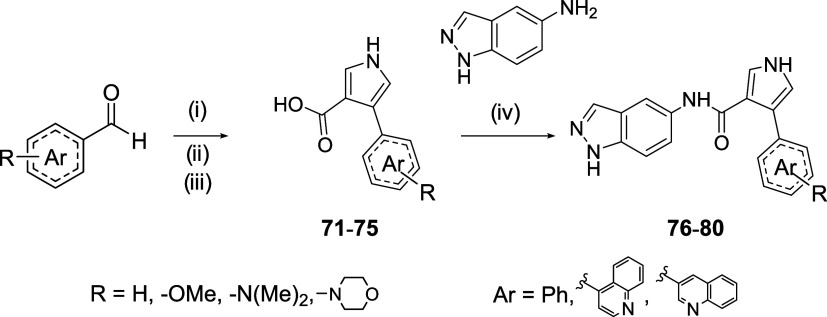
Synthesis of Pyrrole Derivatives with Electron-Donor Substituents in the Aromatic Ring^a^

a (i) Diethyl malonate, piperidine, triethylamine, DCM, 130 °C, 2h, MW; (ii) TosMIC, NaOH, EtOH, 1h to 2h; (iii) NaOH, EtOH, H_2_O, reflux, 1 to 2 h; (iv) BOP, DIPEA, THF, r.t., 2 to 24 h.

Finally, alternatives to the pyrrole ring were explored. The first modifications were aimed at replacing the NH of the pyrrole with alkyl substituents, specifically methyl (in **83**) and 3-(dimethylamino)­propyl (in **85**) moieties. In the first case, we started from the intermediate ester **29**, which in the presence of NaH and CH_3_I, gives the methylated derivative **81**. Under the described conditions, hydrolysis of the ester takes place to obtain the acid **82** which, after the corresponding amidation, gives rise to the final compound **83** (Scheme 5). For the synthesis of compound **85**, a different order of reactions was proposed, since the corresponding pyrrole acid with an aminoalkyl substituent would have an amphoteric character that would hinder its isolation by precipitation in acidic media (Scheme 6). Therefore, intermediate **42** was used to form the amide with the *tert*-butyl 5-amino-1*H*-indazole-1-carboxylate (**84**). Subsequently, the pyrrole was alkylated by using 3-chloro-*N*,*N*-dimethylpropan-1-amine under basic conditions and in the presence of KI as a catalyst for the reaction. After deprotection of the indazole by trifluoroacetic acid (TFA), the final product **85** was obtained (Scheme 6).

**Scheme 5:**
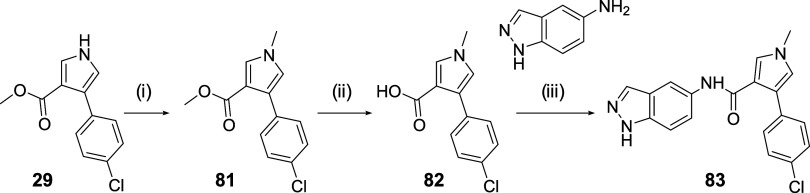
Synthesis of Compound **83** ^a^

a (i) NaH, MeI, DMF, 0 °C to r.t., 2 h; (ii) NaOH, MeOH, H_2_O, reflux, 2 h; (iii) BOP, DIPEA, THF, r.t., 24 h.

**Scheme 6:**
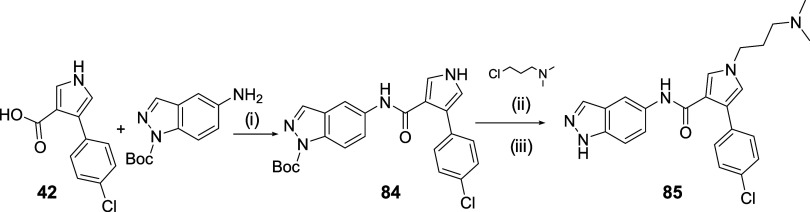
Synthesis of Compound **85** ^a^

a (i) BOP, DIPEA, THF, r.t., 2 h. (ii) Cs_2_CO_3_, KI, DMF, 80 °C, 18 h. (iii) TFA, CH_2_Cl_2_, r.t., 6 h. Boc: *tert*-butoxycarbonyl.

As an alternative to the pyrrolic ring, the five-membered pyrazole heterocycle was proposed (Scheme 7). (*E*)-2-(Nitrovinyl)­benzene derivatives were used as starting materials, which in the presence of a weak base such as triethylamine (TEA) and ethyl diazoacetate led to the formation of ethyl 4-aryl-1*H*-pyrazole-3-carboxylate derivatives.^28,29^ Subsequent hydrolysis afforded the corresponding carboxylic acids **86**-**90**. Finally, amide bond formation was carried out, yielding the final compounds **91**-**95**. An additional advantage of this approach is that it allows the incorporation of π-excessive or electron-rich aromatic rings, such as in compounds **94** and **95**, which is not feasible through our proposed synthesis (Scheme 3). Lastly, other five-membered heterocycles were explored as potential pyrrole replacements, specifically isoxazole (in **96**) and oxazole (in **97**) rings (Scheme 8). These compounds were synthesized in a single-step amidation reaction using the previously described coupling agent BOP.

**Scheme 7:**
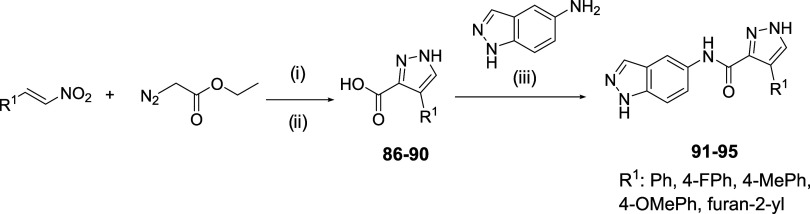
Synthesis of Pyrazole-Type Derivatives **91**-**95** ^a^

a (i) TEA, 24 h; (ii) 37% HCl, reflux, 4 h; (iii) BOP, DIPEA, THF, r.t., 2 to 24 h.

**Scheme 8:**
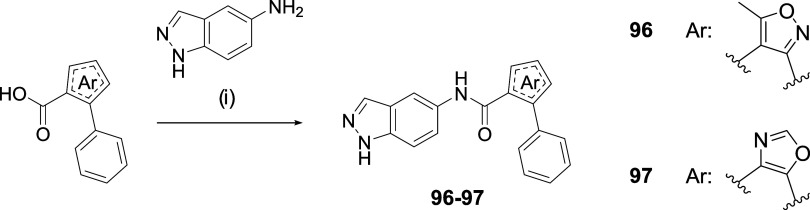
Synthesis of Isoxazole/Oxazole Derivatives **96**-**97** ^a^

a (i) BOP, DIPEA, THF, r.t., 2–24 h.

### *In Vitro* Evaluation of the Inhibitory Activity against SGK1 and Structure–Activity Relationship

Evaluation of the activity of the newly synthesized compounds, along with **H3**, was carried out with the KinaseGlo methodology,^30^ obtaining similar results for the already reported **H3** inhibitory profile (IC_50_ = 0.63 ± 0.01 μM) (Table 1). First, all the compounds were evaluated at a fixed concentration of 10 μM, and only in the cases than the percentage of SGK1 inhibition is greater than 50%, the IC_50_ value was calculated trough a dose response curve. We use as control standards the commercially available SGK1 inhibitors, compounds **GSK650394**, **EMD638683**, and **SGK1-IN-4**, obtaining similar inhibitory activity to that reported in the literature (Figure S1). Data are collected in Table 1. The great majority of the evaluated compounds showed SGK1 inhibition at low micromolar level while some compounds are in the submicromolar region, finding derivatives more potent than the initial hit.

**Table 1:**
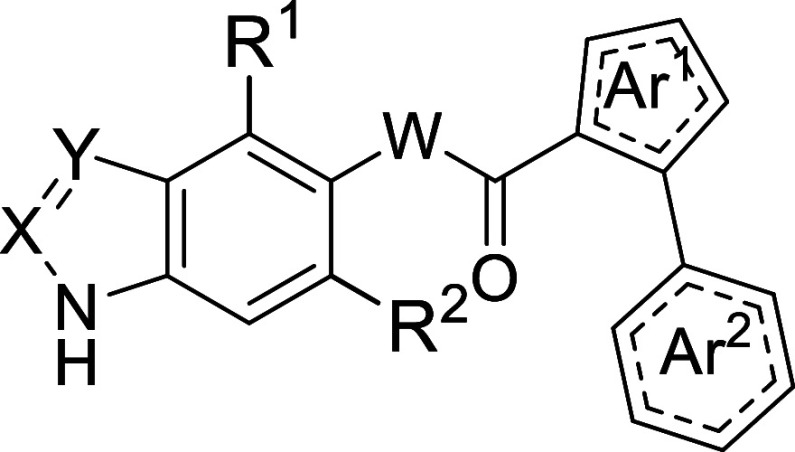

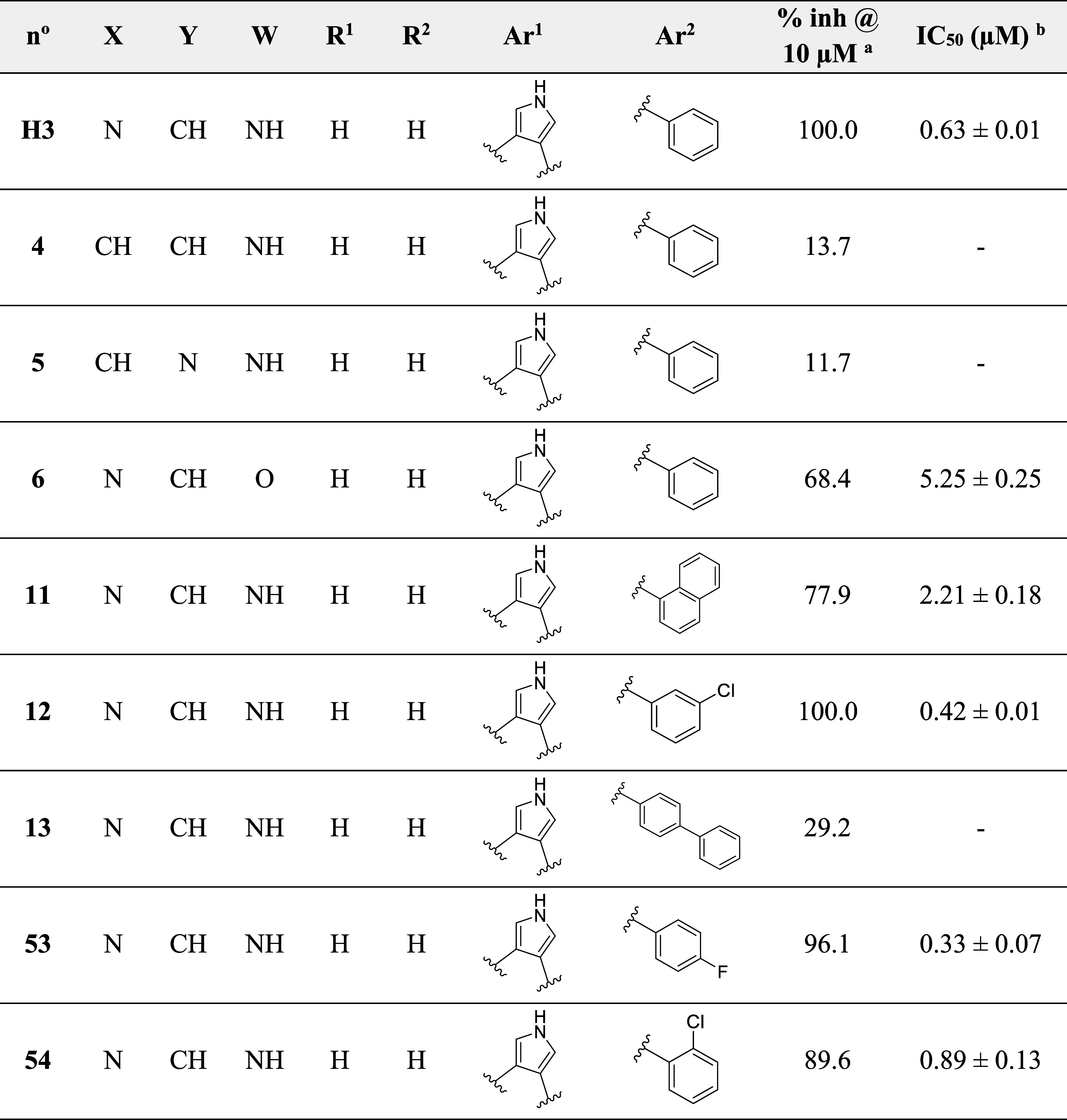

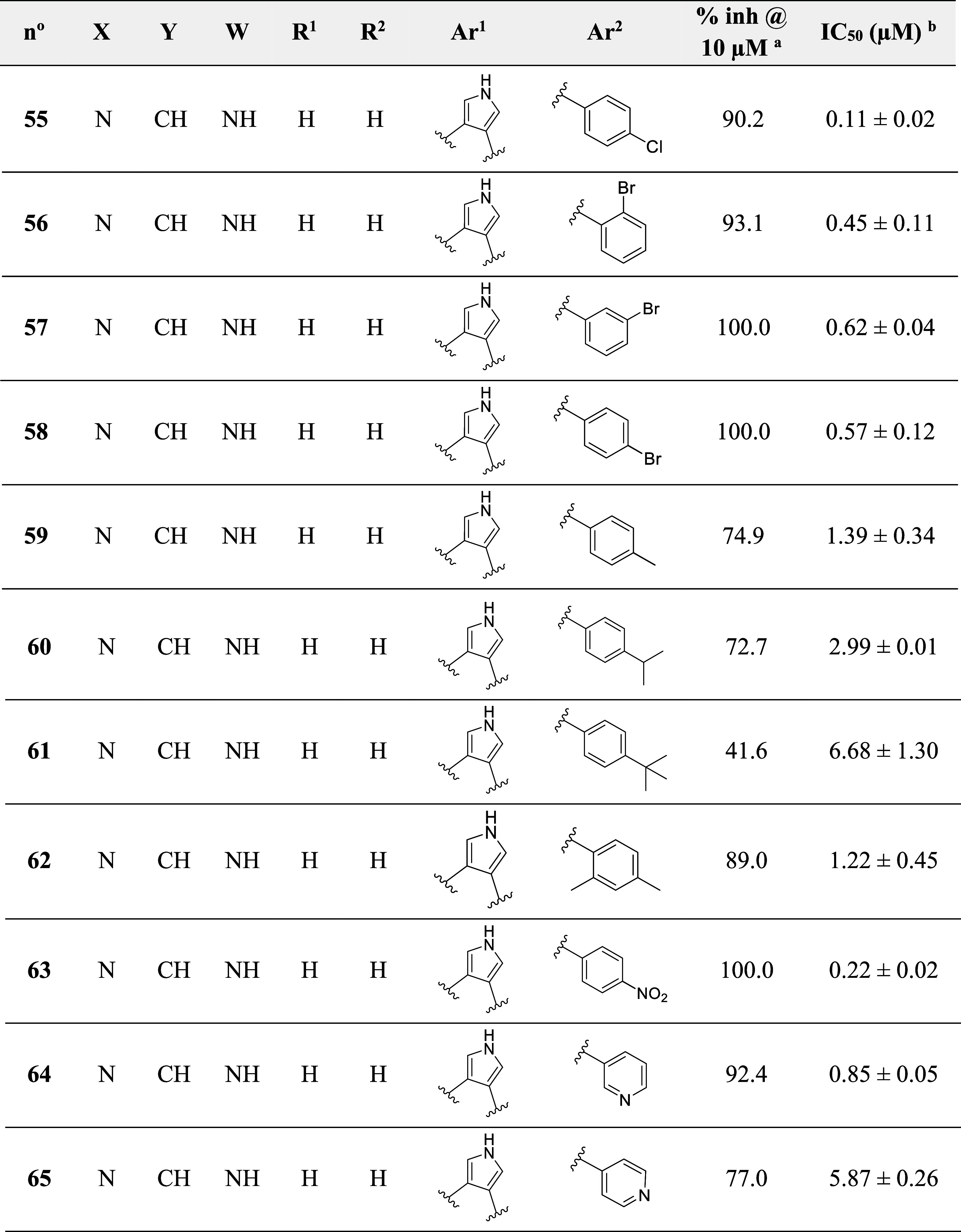

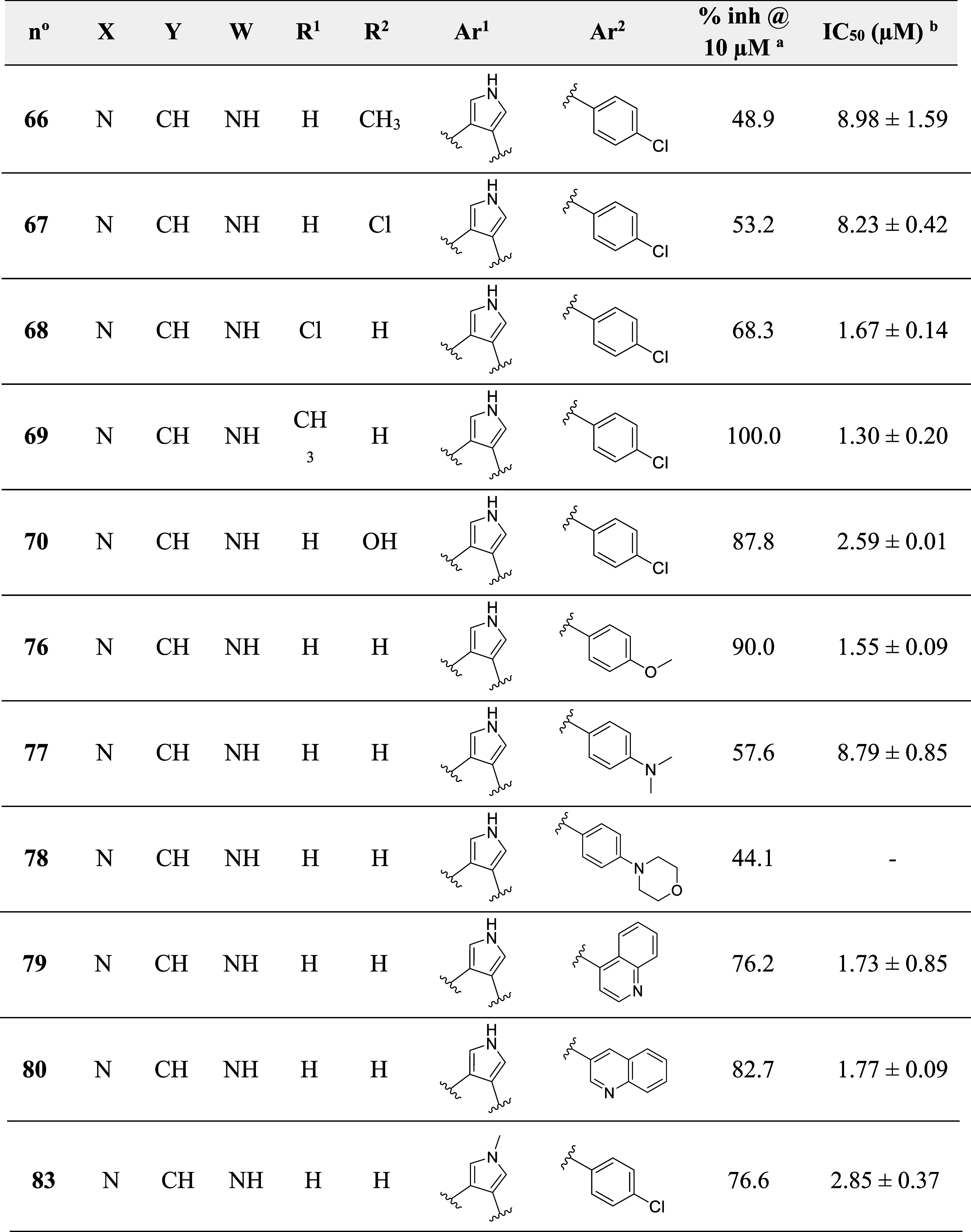

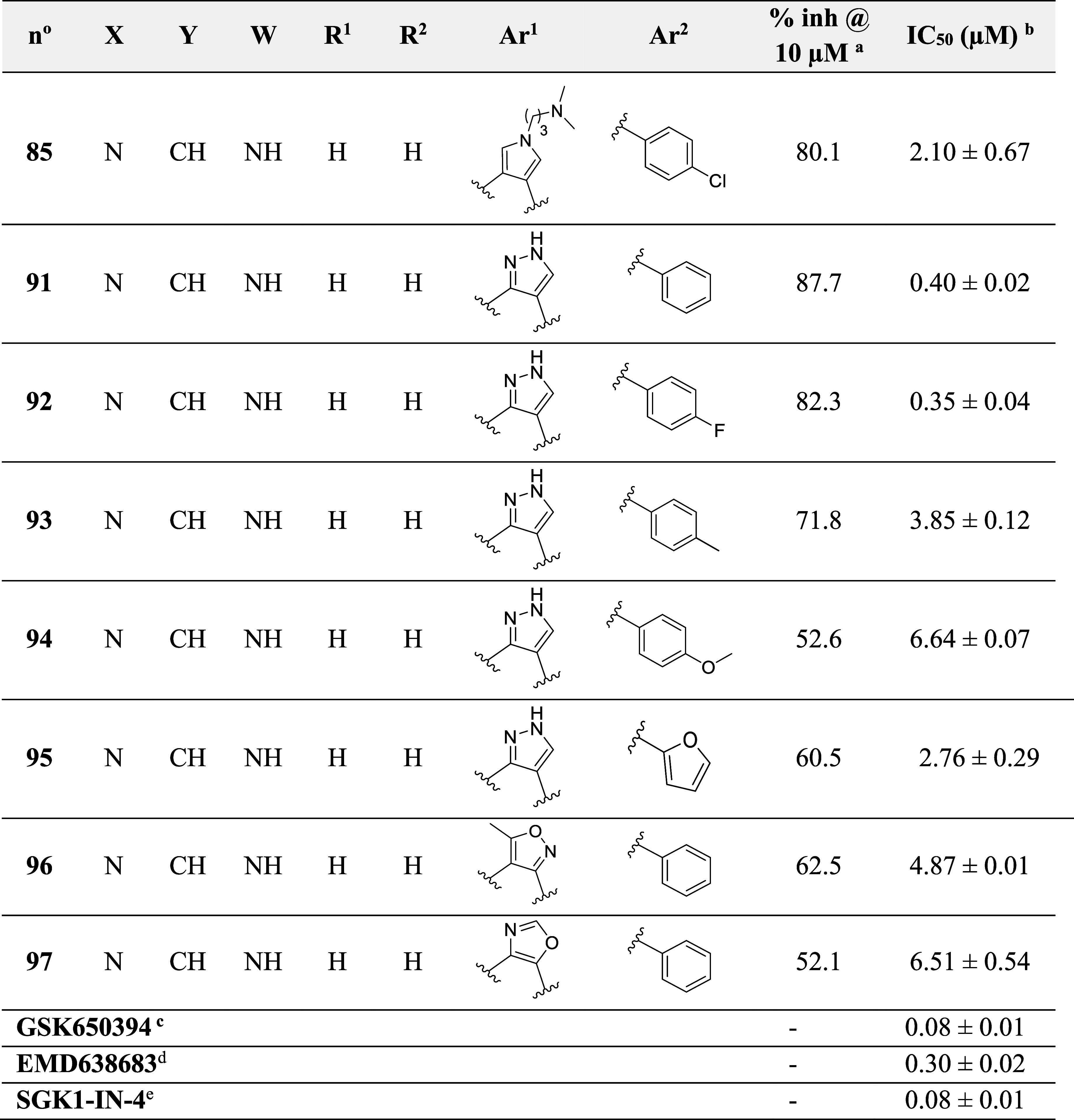
Inhibitory Activity of the Synthesized Compounds against Recombinant Human SGK1 Using the Kinase-Glo Assay

a Percentage of inhibition against SGK1 at 10 μM.

b Half maximal inhibitory concentration. Data are reported as duplicate ± standard deviation.

c Reported IC_50_ = 0.06 μM.^15^

d Reported IC_50_ (cell-based assay) = 3.35 μM.^17^

e Reported IC_50_ = 0.003 μM.^19^

The results showed that replacing the indazole heterocycle present in the hit with either an indole (compound **4**) or a benzimidazole (compound **5**) led to a complete loss of activity. This observation is consistent with the proposed binding mode of **H3** (Figure 1), because these substitutions prevent the formation of a key hydrogen bond. On the other hand, replacing the amide group with an ester (derivative **6**) resulted in reduced activity. Although the amide NH in **H3** may participate in a hydrogen bond with a water molecule (Figure 1), this interaction appears to be less critical than those involving the hinge region, allowing the ester derivative **6** to retain some residual SGK1 activity.

Regarding the phenyl ring in position 4 of the pyrrole core, alkyl substituents such as methyl, isopropyl or *tert*-butyl at position 4 led to a reduction in inhibitory activity for compounds **59–62** compared to **H3**, showing a clear correlation between substituent size and activity loss (Table 1). Similarly, derivatives **11**, **13**, and **78** bearing naphtyl, biphenyl and morpholinylsubstituents respectively, displayed decreased activity, likely for the same reason. Moreover, quinoline derivatives **79** and **80**, as matched pairs of the naphtyl compound **11**, showed similar inhibitory activity, highlighting the hypothesis that the hydrophobic pocket that accommodates the aryl ringcan only toleratea limited increase in steric volume.

On the other hand, the introduction of an halogen and/or electron-withdrawing substituents on the phenyl ring were well tolerated (derivatives **12**, **53**-**58**, **63**), since the IC_50_ always remained in the submicromolar range (Table 1). Notably, compounds **53**, **55**, and **63** with F, Cl and NO_2_ at the position 4 of the ring showed a notable increase of the inhibitory activity in relation to the **H3** (IC_50_ = 0.33, 0.11, and 0.22 μM, respectively). In contrast, when the phenyl ring was decorated with electron-donor substituents, such as methoxyl, dimethyl or morpholinyl (compounds **74–76**), the inhibitory potency decreased. However, it is possible that a significant loss of activity is due to steric hindrance of the substituents. The activity of compounds **64** and **65**, with pyridine substituents in the pyrrole, is striking. While the π-deficient pyridin-3-yl ring was well tolerated, pyridin-4-yl produced a noticeable drop in activity probably due, at least in part, to its higher solvation energy (Tables 1 and S1). Furthermore, slight modifications at positions 4- and 6- of the indazole scaffold (compounds **66–70**) were evaluated, mainly involving chlorine, methyl and hydroxyl substituents. In all cases, a reduction in activity was observed (Table 1). This decrease can be attributed to the steric effects of these substituents, which may induce conformational changes in the molecule, such as amide bond rotation, thereby disrupting the overall hydrogen-bonding interaction network.

When considering the pyrrole ring, the evaluation of the *N*-alkylated compounds **83** and **85**, revealed that the NH group of the pyrrole contributes partially to the interaction with the protein through a hydrogen bond with the Glu226. However, the loss of this interaction in derivatives **83** and **85** is not as critical as that observed with the indazole ring in its binding with the hinge region (Table 1). That is similar to the amide group when replaced by an ester (compound **6** versus **H3**). Replacement of the pyrrole ring with a pyrazole was found to be compatible with protein inhibition (compounds **91** and **92**). As with the pyrrole series, relatively bulky substituents at the 4-position of the phenyl ring (derivative **93**) led to a loss of inhibitory activity (Table 1). Similarly, substitution with π-excessive rings such as the furan **95** or electron-rich systems as **94** also resulted in a decrease of SGK1 inhibition (Table 1). Finally, and in line with was previously described above, substitution of pyrrole by other five-membered rings like isoxazole **96** or oxazole **97** that were unable to participate in hydrogen bonding with Glu226 were found to be less active (Table 1).

In parallel, the binding mode of the different derivatives from this new chemical family was studied by molecular docking. The SGK1 conformation obtained in our previous work was here used, and inhibitors’ conformations within the active site were predicted using Glide (Schrödinger) (Table S2 and Figure 3A). It is well-known that traditional force fields tend to underestimate the energetic penalty associated with adopting the bioactive conformation.^31^ Given the rigid nature of this molecules, this factor can be critical when distinguishing realistic conformations from false positives. Therefore, in addition to the docking score, the strain energy associated with each pose was calculated. The hit compound obtained a notable docking score of −10.828 kcal·mol^–1^ (Table S2). Consistent with the proposed binding mode, analogues **4** and **5** displayed a marked decrease in docking score, whereas derivative **6** showed a comparatively moderate reduction. This finding underscores the critical importance of hydrogen bonding interactions within the hinge region relative to those in the amide region.

**Figure 3:**
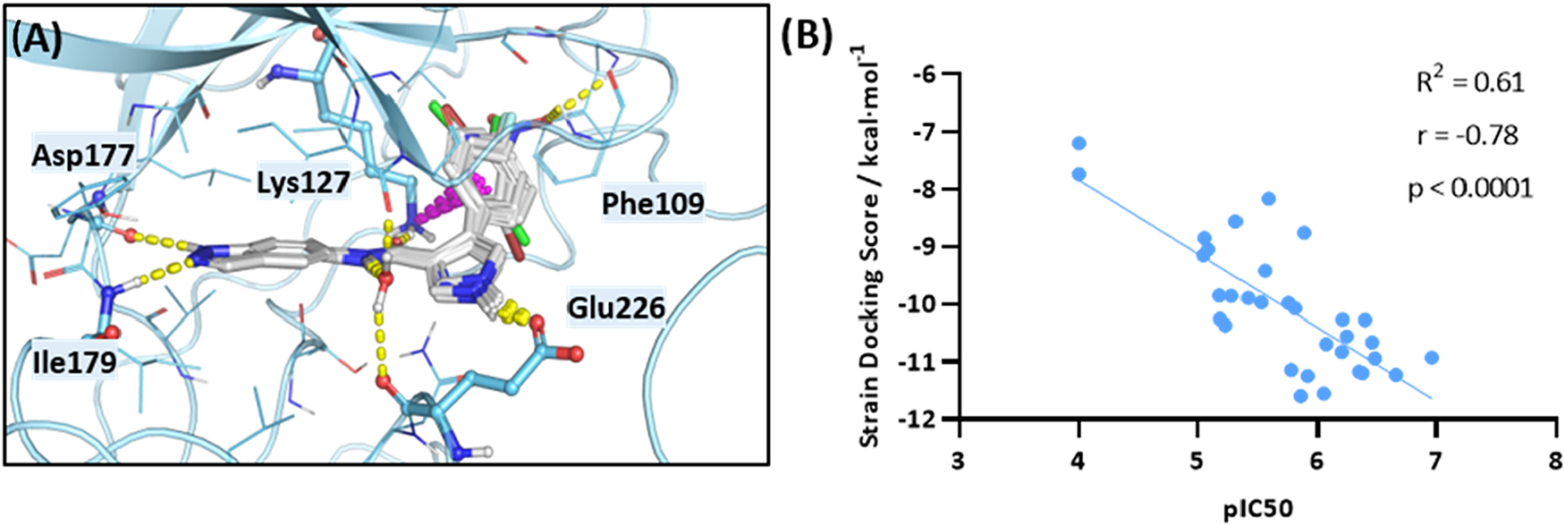
Docking analysis of SGK1 (PDB: 3HDM) with the new synthesized inhibitors. (A) Binding mode of compounds **12**, **53**-**58**, **63**-**64**, and **81–82**. Key interacting amino acids are highlighted as sticks. Yellow, hydrogen bond interaction; purple, π-cation interaction. (B) Lineal regression model of the inhibitors. For compounds **4** and **5**, the IC_50_ was considered as 100 μM. *R* ^2^, coefficient of determination; *r*, Pearson correlation coefficient.

For the phenyl ring in position 4 of the pyrrole ring, the docking score decreased as the size of the substituent increased (Table S2), most notably for compounds **13** and **61**, in which bulky groups caused a complete loss of the binding pose. Although compounds bearing methyl substituents exhibited slightly better docking scores than the reference compound (**59**, −11.594 kcal·mol^–1^; **62**, −11.296 kcal·mol^–1^), clear steric clashes with residue Phe109 were observed (Figure S2A). Given the high flexibility of this loop, molecular dynamics simulations might reveal increased system instability, an effect not captured by conventional docking methodologies.

In the case of the compound with a naphthalene substituent (derivative **11**), steric hindrance induced a rearrangement of the ligand into a coplanar conformation, thereby weakening the interaction and increasing strain energy (Figure S2B and Table S2). Similarly, derivatives modified at positions 4- and 6- of the indazole scaffold (**66** and **70**) adopted highly strained conformations, and the observed loss of activity correlated well with the corresponding strain energy values (Table S2). On the other hand, compounds **53**, **55**, and **63** bearing F, Cl, and NO_2_ substituents at position 4- of the phenyl ring, respectively, exhibited a slight improvement in docking scores (Figure 3A and Table S2).

Regarding the replacement of pyrrole ring, pyrazole derivative **91** achieved a docking score comparable to that of the reference compound (Figure 3A and Table S2). When different substitutions on the phenyl ring in position 4 of the pyrazole were simulated (compounds **92**-**95**, Figures 3A and S2C), the docking scores followed a trend similar to that observed in the pyrrole series (Table S2). Substitutions such as isoxazole **96** or oxazole **97**, which are unable to establish a hydrogen bond interaction with Glu226, exhibited decreased docking scores (Figure S2D). Bulky substitutions at the pyrrole N–H position (derivatives **83** and **85**) caused a loss of the canonical binding conformation due to steric clashes with Glu226. As observed for derivatives **59** and **62**, it is expected that employing alternative computational approaches that incorporate flexibility of Glu226 could yield more realistic binding poses.

Taking all the above into account, we developed a predictive model correlating the experimental activity values with the docking scores. Given the relevance of strain energy for certain compounds, the docking scores were adjusted accordingly. As shown in Figure 3B, both variables exhibit a strong negative correlation, which can be described by a linear regression model (*r* = −0.78, *p* < 0.0001, *R* ^2^ = 0.61).

### Assessment of Brain Permeability of the Inhibitors through PAMPA Methodology

In order to advance the development of the most potent compounds as potential candidates for neurological disorders, it is essential to determine whether they are capable of crossing the BBB. To address this, the Parallel Artificial Membrane Permeability Assay (PAMPA) methodology was employed. This model is based on the use of two compartments (donor and acceptor) separated by an artificial membrane composed of porcine brain lipids that mimics the BBB, allowing us to estimate the brain penetrance of the evaluated molecules by passive diffusion and using as controls drugs in currently therapeutic used with known human brain permeability.^32^ Only the most potent compounds (IC_50_ < 3 μM) were evaluated in the BBB permeability assay, alongside three reference SGK1 inhibitors: **GSK650394**, **EMD638683**, and **SGK1-IN-4**, The results are summarized in Figure 4 and Table S3. Regarding the reference compounds, as expected, their passive permeability was generally poor: **SGK1-IN-4** was not soluble in the mediun used in the assay, while **EMD638683** showed no permeability and **GSK650394** exhibited low probability for passive diffusion. In contrast, 58.3% of the newly tested compounds displayed effective permeability values (*Pe*) consistent with CNS penetration (CNS+). An additional29.2% fell within the uncertain classification range (CNS–/CNS+), while compounds **64**, **70**, **79**, and **80** were categorized as non permeables (CNS)

**Figure 4:**
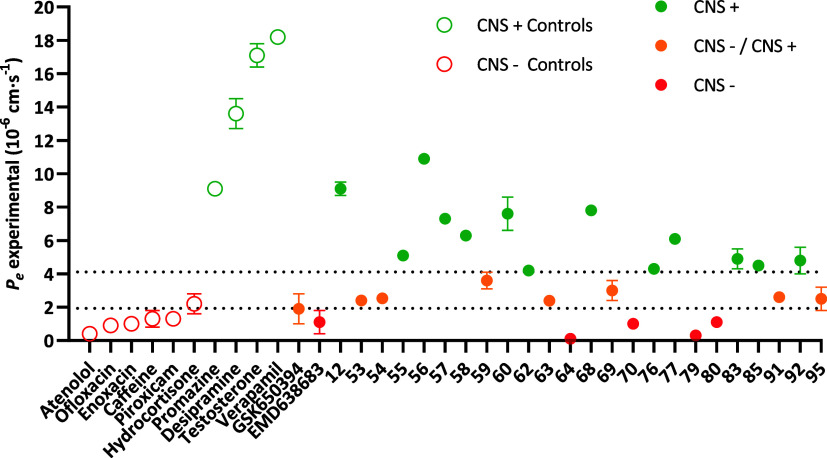
Brain permeability of SGK1 inhibitors according to the PAMPA methodology. **○**, controls; **●**, SGK1 inhibitors; green, CNS+; orange, CNS–/CNS+; red, CNS–. Represented as the mean ± standard deviation of two independent experiments.

### *In Vitro* Evaluation of the Neuroprotective Effect of SGK1 Inhibitors in a Cellular AD-Related Model

Those SGK1 inhibitors with an IC_50_ value <1 μM able of crossing the BBB or in the uncertainty zone, were evaluated in the SH-SY5Y human neuroblastoma cell line. As a first step, cell viability was assessed at concentrations of 1 and 5 μM (Figure 5A). None of the compounds showed a significant reduction in cell viability, except for compound **55** at the highest concentration. Therefore, a concentration of 5 μM was used for all subsequent experiments, except for compound **55**, which was tested at 1 μM.

**Figure 5:**
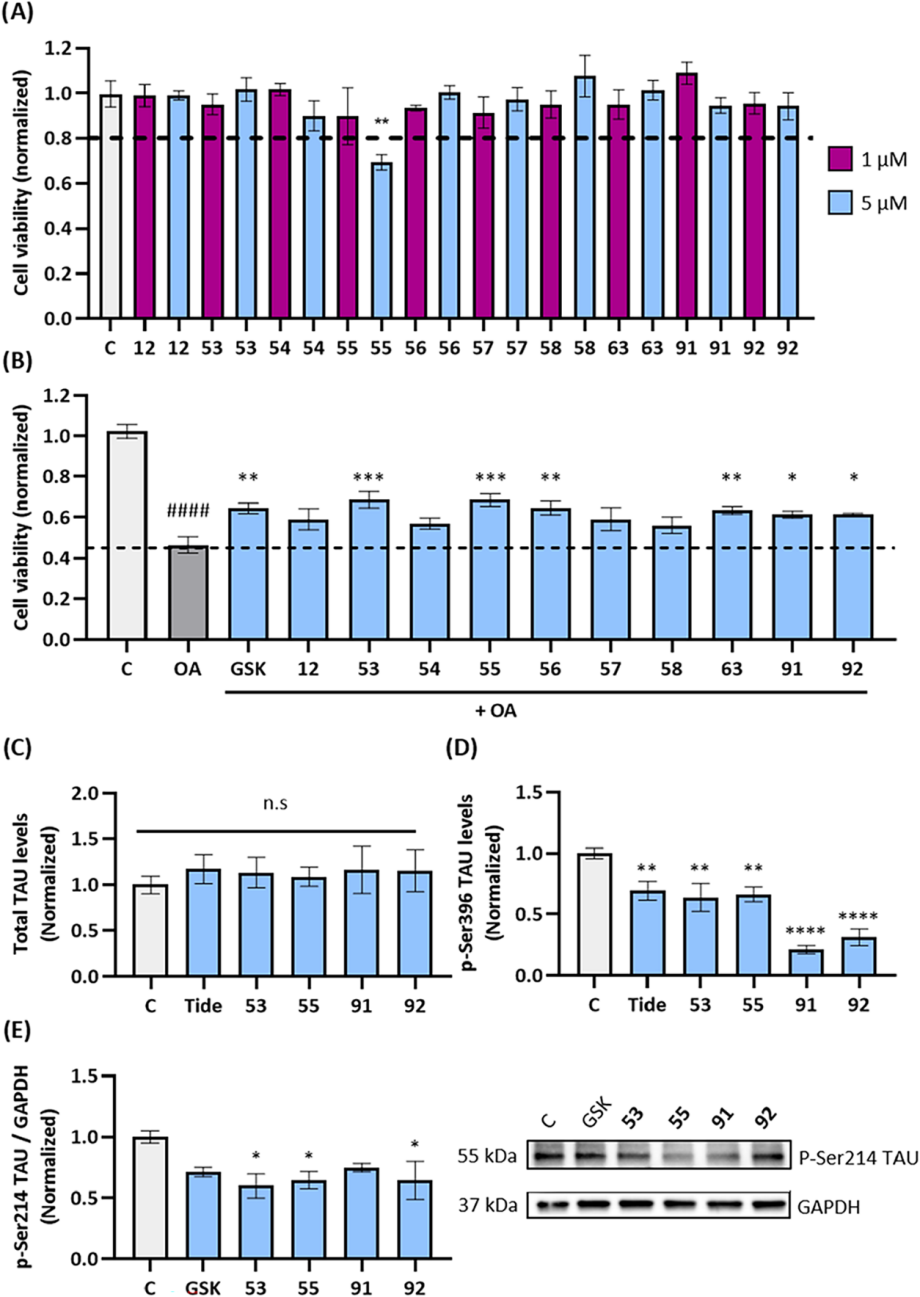
Biological characterization of SGK1 inhibitors in SH-SY5Y cell lines. (A) Cell viability of SGK1 inhibitors at 1 and 5 μM measured with MTT after 24 h. *n* = 3 independent experiments. (B) Neuroprotective effects of the inhibitors in the presence of OA, measured with MTT, after 24 h. *, O.A vs treatment; #, O.A vs Control. *n* ≥ 3 independent experiments. (C) ELISA measures of total TAU levels quantified in the protein extracts of SH-SY5Y cells treated with SGK1 inhibitors. *n* ≥ 3 independent experiments. (D) p-Ser396 levels in the protein extracts measured by ELISA. *n* ≥ 3 independent experiments. (E) Representative immunoblot showing p-Ser214 levels of SH-SY5Y cells treated with the compounds. *n* = 3 independent experiments. All the compounds were tested at 5 μM except **55** (1 μM). Data is represented as the mean ± SEM. Statistical significance was assessed using ANOVA test with Dunnett’s *posthoc* correction. Asterisks denote statistical significance: *, *p*-value <0.05; **, *p*-value <0.01; ***, *p*-value <0.001; ****, *p*-value <0.0001; n.s, no significant differences. OA, okadaic acid at 30 nM; GSK, **GSK650394** at 10 μM; Tide, **Tideglusib** at 10 μM.

The neuroprotective effects of SGK1 inhibitors were evaluated using a cellular model of AD based on okadaic acid (OA) exposure to induce TAU hyperphosphorylation.^33^ OA inhibits phosphatases 1 (PP1) and 2A (PP2A), disrupting the equilibrium between phosphorylation and dephosphorylation of numerous cellular substrates. This imbalance activates multiple kinases, resulting in TAU hyperphosphorylation and ultimately causes neuronal death. Previous studies have shown that OA increases SGK1 activity,^34^ likely because PP2A normally dephosphorylates and inactivates SGK1.^35^ In this context, we first assessed the ability of our inhibitors to counteract OA-induced toxicity (Figure 5B). Cells were treated with the compounds at the indicated concentrations, followed by OA addiction at a final concentration of 30 nM. The results revealed that most compounds partially restored cell viability in the presence of OA. Statistically significant protection was observed for compounds **53**, **55**, **56**, **63**, **91**, and **92**, as well as the positive control **GSK650394**, a well-established SGK1 inhibitor.^36^

Given the observed neuroprotective efficacy of these inhibitors, a second step involved evaluating whether compounds **53**, **55**, **91**, and **92** could modulate TAU phosphorylation levels using ELISA and Western Blot analysis. SGK1 is known to phosphorylate TAU at Ser214,^37^ an epitope found to be increased in several AD-related models, including Tg106 or Tg-Prp-TAU^P301S^.^38,39^ Moreover, SGK1 has been shown to activate GSK3β, the main kinase responsible for TAU phosphorylation at Ser396.^38^ Therefore, we proceeded investigated whether SGK1 inhibitors could reduce TAU phosphorylation at both Ser214 and Ser396 sites.

First, an ELISA kit was used to verify that the SGK1 inhibitors did not alter total TAU levels after their incubation in SH-SY5Y cells. Tideglusib, a well-known inhibitor of GSK3β,^40^ was used as control reference (Figure 5C). Next, we proceeded to measure the level of phosphorylation of TAU at the Ser396 with an ELISA kit. Results indicated that the compounds were able to reduce this phosphorylation, with a remarkable decrease for the compounds **91** and **92**, which even exceeded the positive control (Figure 5D). Given the presence of a pyrazole ring in both compounds, we hypothesize that this structural difference may be responsible for the observed behavior. *In vitro* evaluation of the inhibitory activity against GSK3β^41^ of the four compounds showed that the pyrazole series were indeed dual inhibitors of SGK1 and GSK3β, with IC_50_ values in the nanomolar range for this last kinase (**91**, IC_50_ 0.04 ± 0.01 μM; **92**, IC_50_ 0.03 ± 0.01 μM). This potent GSK3β inhibitory activity was not observed for pyrroles **53** and **55** (**53**, IC_50_ 5.17 ± 0.70 μM; **55**, IC_50_ 3.39 ± 0.23 μM), suggesting that this dual inhibition is responsible for the marked decrease in phosphorylation levels. Second, the phosphorylation of the Ser214 epitope was quantified using Western blot analysis. The results showed that the inhibitors caused a tendency to decrease p-Ser214 levels, with statistically significant differences for compounds **53**, **55**, and **92.** In this assay, the known SGK1 inhibitor, **GSK650394**, was used as positive control (Figure 5E,F).

Based on these findings, compound **55** was prioritized for further development owing to its superior inhibitory activity, effective permeability in the PAMPA assay, and demonstrated neuroprotective effect in the SH-SY5Y cell line.

### *In Vivo* Pharmacokinetic Study of the SGK1 Inhibitors

In drug design, there are additional properties of a candidate compound beyond potency that must be characterized, such as selectivity and preclinical safety. In this regard, compound **55**, a BBB permeable compound based on PAMPA assay, proved to be a selective SGK1 inhibitor against an extensive kinase panel, and showed no signs of cardiotoxicity or mutagenic activity.^42^ This profile indicates that it is a suitable subject for evaluation in *in vivo* models related to neurodegeneration. To this end, and to determine an appropriate administration dose for the potential *in vivo* treatment, the pharmacokinetic profile of the compound was evaluated in BALB/c mice (Table 2). These animals received a single dose of 5 mg·kg^–1^ intraperitoneally (i.p.) and 10 mg·kg^–1^ orally (p.o.), observing a peak plasma concentration at 0.25 h in both cases and suggesting a fast absorption. However, regarding the brain distribution, the exposition was drastically low, as evidenced by the brain/plasma ratio (Brain-*K*_p_) of 0.04. To determine whether this unexpected behavior is specific to compound **55** or instead a characteristic of the entire inhibitor series, the same experiment was conducted with compound **53**. Similarly, brain levels also exhibited the same pattern (Brain-*K*_p_ 0.02), which may indicate an issue beyond the physicochemical properties of the compounds that define their passive diffusion.

**Table 2:** Pharmacokinetic Profiles of Compounds **53** and **55** after Single Dose Administration in Male BALB/c Mice^a^

*N*°	route	dose (mg·kg^–1^)	matrix	*T*_max_ (h)	*C*_max_ (ng·mL^–1^)	AUC_last_ (h·ng·mL^–1^)	*T*_1/2_ (h)	brain-*K*_p_ (AUC_last_)
**55** ^b^	i.p.	5	Plasma	0.25	568.84	591.58	0.55	-
			Brain	0.25	28.67	23.50	-	0.04
	p.o.	10	Plasma	0.25	777.40	599.08	0.33	-
			Brain	0.25	34.72	25.19	-	0.04
**53**	i.p.	5	Plasma	0.25	884.38	532.48	0.47	-
			Brain	0.25	25.74	9.75	-	0.02
	p.o.	10	Plasma	0.25	866.09	741.63	1.14	-
			Brain	0.25	26.49	15.53	-	0.02

a Brain *C*_max_ and AUC_last_ are expressed as ng·g^–1^ and h·ng·g^–1^, respectively. Density of brain tissue was considered as 1 which is equivalent to plasma density.

b Data of compound **55** were obtained from the corresponding ref 42.

### Analysis of Active Transport Mediated by P-Glycoprotein

Although passive diffusion across the BBB is a key property to optimize in CNS-targeted drug candidates, brain exposure can also be influenced by additional factors. Notably, endothelial cells forming the BBB express efflux transporters such as P-glycoprotein (P-gp), which actively recognize and eject substrates, preventing their accumulation in the brain. The possibility that this series of inhibitors could be P-gp substrates arose from the similarly low brain concentrations observed for compounds **53** and **55**. This efflux mechanism often limits the bioavailability of kinase inhibitors developed for neurodegenerative diseases and has been widely discussed in the field.^43^

To investigate the potential involvement of P-gp in the pharmacokinetics of compound **55**, the AI-driven PgpRules program was employed.^44^ This program first predicts whether a compound is a P-gp substrate and then provides molecular descriptors supporting the prediction (Table S4), which can guide future chemical modifications to minimize efflux. The prediction indicated that compound **55**, is likely a P-gp substrate. To further explore the predicted binding mode, an induced-fit docking simulation,^45^ using the crystallized P-gp structure (PDB 6QEX). The resulting P-gp-**55** complex revealed key interactions mediated by pyrrole hydrogen bonding and π-π stacking of the phenyl ring (Figure 6), consistent with previously described P-gp substrate binding patterns.^46^

**Figure 6:**
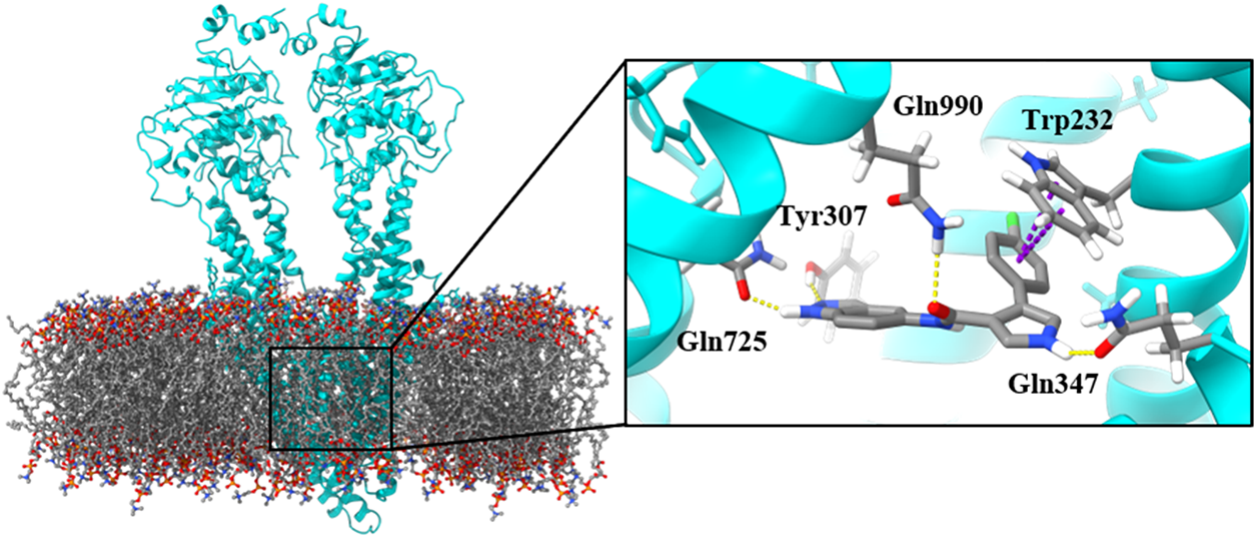
Binding mode between P-gp (PDB: 6QEX) and compound **55**. On the left, the protein embedded in the plasma membrane. On the right, compound **55** at the binding site. In yellow, hydrogen bond interactions. In purple, π–π coupling interactions.

Based on the available data, we analyzed which structural modifications could prevent active efflux. Previous studies indicate that high topological polar surface area and an increased number of hydrogen bond donors are key determinants of P-gp recognition.^47^ Thus, reducing these parameters may help to avoid efflux. According to PgpRules, the polar hydrogen of the pyrrole ring is the main contributor to P-gp binding, consistent with our proposed model in which pyrrole forms a hydrogen bond with Gln347 (Figure 6). Assuming this hypothesis, compound **83**, with its alkylated pyrrole, should not interact with P-gp. Indeed, PgpRules predicted it as a nonsubstrate (Table S4). To confirm this, bidirectional permeability assays were performed for compounds **55** and **83** using the MDCKII-MDR1 cell line, which overexpresses P-gp (Table 3).

**Table 3:** Bidirectional Permeability across MDCKII-MDR1 Cell Monolayers in Presence and Absence of P-gp Chemical Inhibitor Zosuquidar^a^

compound	AP-BL Papp/10^–6^ cm·sec^–1^	BL-AP Papp/10^–6^ cm·sec^–1^	efflux ratio
**Loperamide** ^b^	1.73	37.80	21.83
**Loperamide** ^b^ **+ ZSQ**	14.18	9.12	0.64
**Atenolol** ^c^	0.16	0.30	1.88
**Propranolol** ^d^	15.30	14.03	0.92
**55**	8.70	25.22	2.90
**55 + ZSQ**	17.32	11.80	0.68
**83**	20.13	23.98	1.19
**83 + ZSQ**	23.13	20.26	0.88

a ZSQ: zosuquidar.

b Loperamide as positive control of substrate recognition.

c Atenolol as low permeability control.

d Propranolol as high permeability control.

Compound **55** showed moderate absorptive and high secretory permeability, yielding an efflux ratio (*R*_E_) of 2.90. Upon coincubation with zosuquidar, a P-gp inhibitor, *R*_E_ decreased to 0.68, confirming P-gp–mediated efflux. In contrast, compound **83**, displayed high permeability in both conditions (*R*_E_ = 1.19 and 0.88), indicating it is not a P-gp substrate. Pharmacokinetic studies in mice further supported this, as compound **83** achieved higher brain levels than compound **55** (Brain-*K*_p_ 0.19, Table S5). Thus, pyrrole methylation effectively enhances brain penetration in this compound series, despite the associated reduction in activity (Table 1).

### Design, Synthesis, and Characterization of a Selective and Brain-Penetrant SGK1 Inhibitor

The main limitation of SGK1 inhibitor **83** is its relatively low potency (IC_50_ = 2.85 ± 0.37 μM, Table 1). To identify more active analogues that are not P-gp substrates, new structural modifications were designed to improve its activity. Based on the proposed binding mode, the phenyl ring occupies a hydrophobic pocket; thus, a derivative bearing two chlorine atoms at the 3- and 4-positions of the phenyl ring and a methylated pyrrole NH (compound **102**), was proposed and synthesized following the synthetic procedure previously optimized (Scheme 9). This modification is expected to increase ring lipophilicity and, consequently, enhance inhibitory potency.

**Scheme 9:**
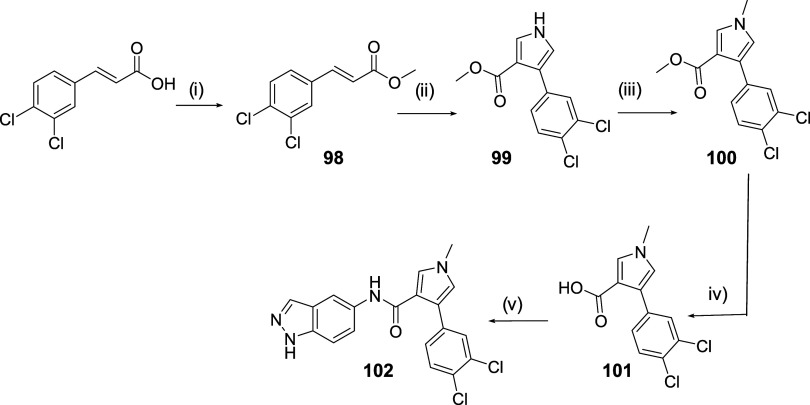
Synthesis of Compound **102** ^a^

a (i) TMSCl, MeOH, r.t., 24 h. (ii) TosMIC, NaH, DMF, 0 °C to r.t., 1 h. (iii) NaH, MeI, DMF, 0 °C to r.t., 2 h. (iv) NaOH, MeOH, H_2_O, reflux, 2 h. (v) 1*H*-indazol-5-amine, BOP, DIPEA, THF, r.t., 24 h.

The inhibitory activity of compound **102** was evaluated, obtaining an IC_50_ of 0.72 ± 0.24 μM, remaining in the submicromolar range (Table 4) and serving as a good starting point for the design of future inhibitors. Consequently, favorable permeability values were found in the PAMPA assay (Table 4), being considered CNS+ in terms of passive diffusion. In addition, AMES and ionic channel inhibition tests, including hERG,were performed as a preclinical safety measure, showing that the compound can be considered nonmutagenic and with no potential human cardiac issues, despite a slight inhibition of Cav1.2 channels (Table 4).

**Table 4:** Activity, Permeability, and Preclinical Safety Characterization of Compound **102**

*N*°	IC_50_ (μM)^a^	*P*_*e*_/10^–6^ cm·s^–1^ ^b^	AMES test	hERG inhibition (μM)	Nav1.5 inhibition (μM)	Cav1.2 inhibition (μM)
**102**	0.72 ± 0.24	4.5 ± 0.1 (CNS+)	negative	>50	>50	12.1

a Half-maximal inhibitory concentration.

b Effective permeability according to the PAMPA assay.

In the same way as the previous compounds, the biological characterization of compound **102** was done. The candidate showed no toxicity at a concentration of 5 μM and, moreover, exhibited a neuroprotective profile in the presence of OA (Figure 7A). Regarding the regulation of tau, the compound was able to show a clear reduction in the phosphorylation of epitope 396, and a slight—although significant—reduction of epitope 214 (Figure 7B,C). This behavior underscores the potential of this inhibitor as a therapeutic agent in tauopathies such as AD. In terms of kinase selectivity, compound **102** was evaluated against a panel of 140 diverse kinases at 10 μM, with a S_20_ score of 0.06, which indicates a selective compound (Table 4), despite some activity against kinases related to the AGC family, such as ROCK2, RSK2 and MSK1, or ERK family (Figure 7D and Table S6). Given these data, it is worth highlighting the relationship of these inhibitory activities with TAU homeostasis and AD. Within the AGC kinase family, in addition to SGK1, kinases such as MSK1 and RSK2 have been shown to phosphorylate TAU at the Ser214 epitope.^37^ To date, no direct association between TAU phosphorylation and CLK2 has been reported, and although ROCK2 is capable of phosphorylating TAU,^48^ its primary contribution to tau homeostasis appears to be linked to the regulation of autophagy.^49^ In contrast, ERK1/2—but not ERK8—can phosphorylate TAU at multiple sites, including Ser396, albeit predominantly under pathological conditions.^50,51^ Accordingly, ERK1/2 ^50^ and ROCK2 ^52^ have been positioned as interesting targets in tauopathies such as AD. These off-target inhibitions, although potentially beneficial in the context of tauopathies, should be carefully taken into consideration in the future.

**Figure 7:**
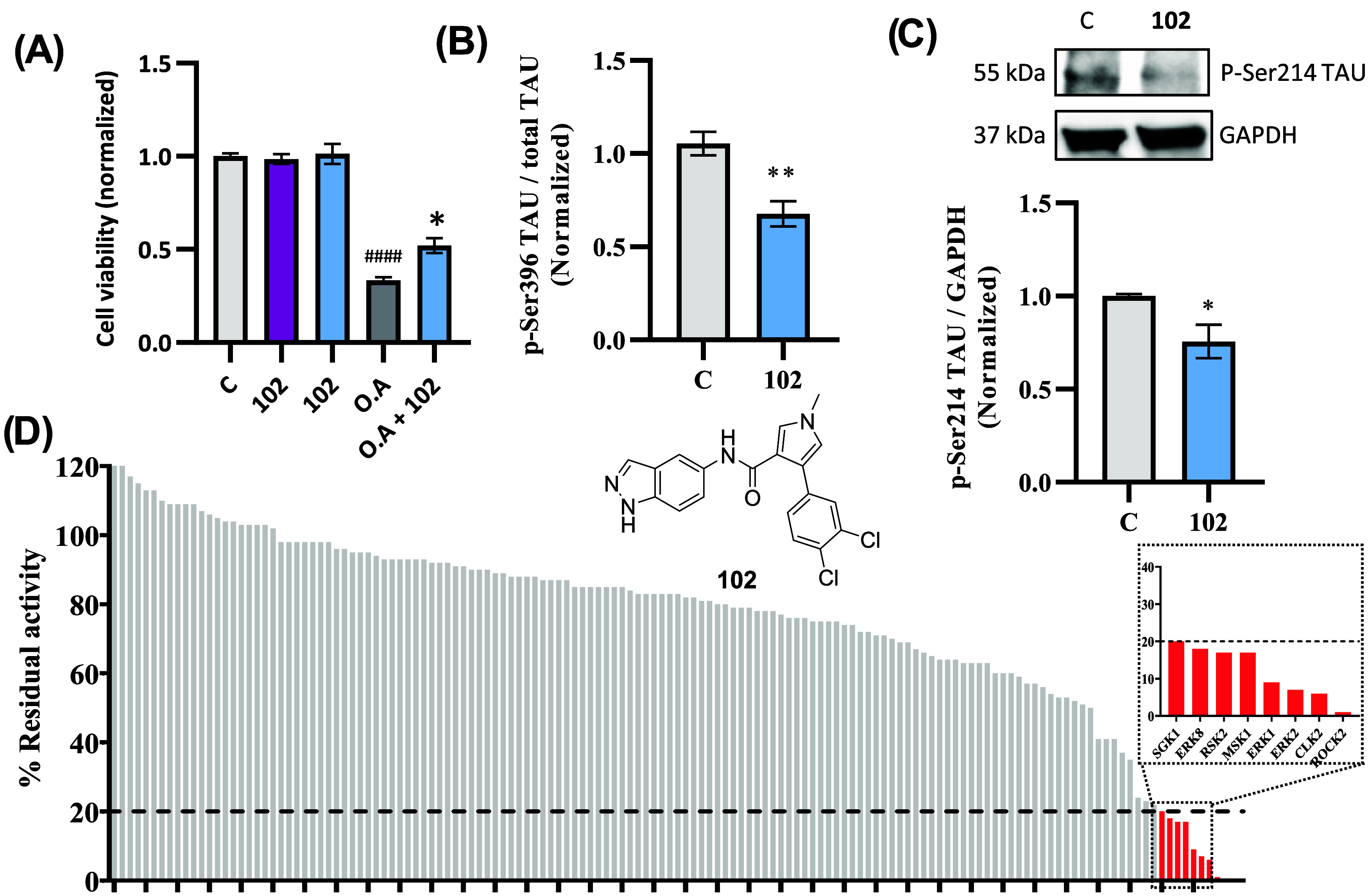
Biological characterization of compound **102**. (A) Neuroprotective effects of the inhibitors in the presence of OA, measured by the MTT assay after 24 h. *, OA vs treatment; #, OA vs control. *n* = 4 independent experiments. (B) p-Ser396 levels in protein extracts measured by ELISA. *n* = 4 independent experiments. (C) Representative immunoblot showing p-Ser214 levels in SH-SY5Y cells treated with the compounds. *n* = 4 independent experiments. (D) Selectivity profile of compound **102** against a panel of 140 kinases at a fixed concentration of 10 μM. Kinases with residual activity below 20% are shown on the right. Data are presented as mean ± SEM. Statistical significance was assessed using Student’s *t*-test or ANOVA with Tukey’s correction for multiple comparations. Asterisks indicate statistical significance: *, *p* < 0.05; **, *p* < 0.01; ****, *p* < 0.0001. OA, okadaic acid (30 nM).

Finally, as a way of validating the obtained results, a pharmacokinetic study *in vivo* of compound **102** was conducted. Plasma and brain levels were analyzed after administration of a single dose orally (p.o., 10 mg·kg^–1^), intraperitoneally (i.p., 5 mg·kg^–1^), and intravenously (i.v., 2.5 mg·kg^–1^) in BALB/c male mice (Table 5). It is noteworthy that the half-life values of compound **102** in this experiment are 0.93, 1.24, and 1.83 h for i.v., i.p., and p.o. administration, respectively. Metabolic stability studies were conducted using mouse, human and minipig liver microsomes, showing high clearance in mouse and medium in human and minipig microsomes (Table S7). *In silico* calculations indicated that methylation of the pyrrolic ring introduces a new metabolic hotspot in the molecule, which may lead to either hydroxylation or *N*-demethylation of the compound (Figure S3). Future efforts should be directed toward addressing this behavior as optimization of this candidate progresses. Regarding CNS penetration, Brain-*K*_p_ values of 0.25, 0.18, and 0.49 were obtained for each of the routes of administration, respectively. These cerebral permeability values were significantly higher than those of compound **55** (Table 2), which validates avoiding active transport of P-gp as a medicinal chemistry strategy to increase the presence of this type of inhibitors in the CNS.

**Table 5:** Pharmacokinetic Profiles of Compound **102** after Single Dose Administration in Male BALB/c Mice

*N*°	route	dose (mg·kg^–1^)	matrix	*T*_max_ (h)	*C*_0_ [Table-fn t5fn2]–*C*_max_ (ng·mL^–1^)	AUC_last_ (h·ng·mL^–1^)	*T*_1/2_ (h)	brain-*K*_p_ (AUC_last_)	%*F*
**102**	i.v.	2.5	Plasma	-	2138.85	954.87	0.93	-	-
			Brain	0.08	958.35	467.79	-	0.49	-
	i.p.	5	Plasma	0.25	902.54	793.15	1.24	-	-
			Brain	0.50	194.08	141.95	-	0.18	-
	p.o.	10	Plasma	0.50	360.06	806.97	1.83	-	21
			Brain	0.50	82.61	200.17	-	0.25	-

Brain *C*_max_ and AUC_last_ are expressed as ng·g^–1^ and h·ng·g^–1^, respectively. Density of brain tissue was considered as 1 which is equivalent to plasma density.

a Back extrapolated concentrations at *t* = 0 for i.v. *C*_max_ value (1666.24 ng/mL) for i.v. arm was considered for brain *K*_p_ calculations and not *C*_0_.

## Conclusions

SGK1 is an underexplored kinase in CNS diseases, with contradictory findings in the literature largely due to the absence of selective, brain-permeable inhibitors. Starting from a previously identified brain-permeable hit and guided by a rational design based on the proposed binding mode, we developed a novel chemical family of SGK1 inhibitors that showed a coherent structure and activity relationship, leading a subset of submicromolar inhibitors with brain permeability and neuroprotective activity in a cellular AD model. These compounds reduced TAU phosphorylation both directly and indirectly through GSK-3 modulation. Although they exhibited passive permeability, *in vivo* studies revealed low brain levels due to P-gp efflux. Using AI-based modeling and molecular docking, we identified the key structural features responsible for P-gp binding. By removing this moiety and introducing subtle modifications to enhance inhibitory potency, we generated a new SGK1 inhibitor, compound **102**, that is selective, brain-permeable, and potentially safe according to preclinical studies. This compound enables the study of SGK1 function in CNS pathologies such as AD and represents a promising starting point for additional optimization to the clinical candidate. Further studies to confirm their activity *in vitro* and *in vivo* are in progress. Overall, these findings highlight SGK1 as a potential therapeutic target for neurodegenerative diseases.

## Experimental Section

### Chemistry

All the reactions were carried out using analytical grade solvents and were obtained from Sigma-Aldrich. If needed, reactions were performed under an inter atmosphere of argon. Reactions were monitored using thin layer chromatography (TLC) with precoated aluminum foils (Merck, 60 F254, 0.2 mm). Melting points were obtained using a Büchi Melting point M-560 apparatus. NMR spectra, both ^1^H and ^13^C, were recorded on a Bruker AV 300 MHz or a 500 MHz located at the NMR unit of Research Assistance Centers from Complutense University of Madrid. Chemical shifts (δ) are expressed in parts per million (ppm), using the indicated deuterated solvent as reference. For each molecule, signal multiplicities (s, singlet; d, doublet; t, triplet; q, quartet; hept, heptuplet; m, multiplet) and coupling constants (*J*, Hz) are described. Spectroscopy data were analyzed using MestReNova software (v.12.0.0). Column chromatography was performed on silica gel 60 (Merck). High resolution mass spectra (HRMS) were obtained using a spectrometer (Agilent 6500) with ESI/APCI ionization source and quadrupole/time-of-flight (QTOF), which is coupled to an Agilent 1200 liquid chromatograph equipped with a Phenomenex Luna C18(2) reversed phase column (100 mm × 2.1 mm, 3 μm packing diameter) located at the Mass Spectrometry Service of the Institute of General Organic Chemistry (IQOG-CSIC). Values are expressed in mass units (*m*/*z*). The HPLC conditions for purity assessment were as follows: HPLC Surveyor equipped with a PDA Surveyor plus UV–vis detector; ZORBAX SB-C18 column (3.5 μm, 4.6 mm × 50 mm). The eluent was H_2_O (0.1% CH_2_O_2_)/CH_3_CN (0.09% CH_2_O_2_) at a flow rate of 0.8 mL·min^–1^ and 23 °C, with an injection volume of 5 μL, using the following gradient: initial CH_3_CN concentration of 5%, linear increase to 100% over 3 min, held at 100% for 1.45 min, followed by a linear decrease back to 5% over 0.55 min. All the evaluated compounds are >95% pure by HPLC analysis. Full synthesis of the intermediates compounds are in the Supporting Information.

#### General Procedure A for the Synthesis of **1**, **14–26**, **98**

The corresponding (*E*)-3-arylacrylic acid (1.0 equiv) was dissolved in MeOH (2 mL·mmol^–1^). Immediately afterward, and at rt, TMSCl (2.2 equiv) was added dropwise. The reaction mixture was stirred for 24 h. The MeOH was evaporated under reduced pressure, and the resulting crude product was dissolved in EtOAc (2 mL·mmol^–1^). The solution was washed with H_2_O (3 × 2 mL·mmol^–1^), the organic phase was dried over anhydrous Na_2_SO_4_, filtered, and finally evaporated under reduced pressure to yield the corresponding (*E*)-methyl 3-arylacrylate.

#### General Procedure B for the Synthesis of **7–10**

A solution of the corresponding (*E*)-3-arylacrylic acid (1.0 equiv) in anhydrous DMF (1 mL·mmol^–1^) under an argon atmosphere was treated dropwise with DIPEA (3.0 equiv) and stirred for 15 min at r.t. Then, a mixture of HBTU (1.5 equiv) and the corresponding amine (1.1 equiv) in anhydrous DMF (1 mL·mmol^–1^) was added dropwise. The reaction mixture was stirred for 2 h, after which EtOAc (5 mL·mmol^–1^) was added. If the final product did not precipitate, the organic phase was washed with H_2_O (3 × 5 mL·mmol^–1^), dried over anhydrous Na_2_SO_4_, filtered, and evaporated under reduced pressure. The compound was then purified by column chromatography as described in each specific case.

#### General Procedure C for the Synthesis of **2**, **11–13**, **27–39**, **99**

A mixture of TosMIC (1.1 equiv) and the corresponding olefin (1.0 equiv) was dissolved in anhydrous DMF (0.5 mL·mmol^–1^) under an argon atmosphere. This solution was added dropwise to a suspension of NaH (3.0 equiv, 60% dispersion) in anhydrous DMF (0.5 mL·mmol^–1^) at 0 °C. After the addition, the reaction mixture was stirred at r.t. for 1–2 h. Subsequently, 2 mL of H_2_O were added, and the mixture was diluted with EtOAc (5 mL·mmol^–1^) and washed vigorously with a 1:1 mixture of H_2_O and saturated NaCl solution (5 × 5 mL·mmol^–1^) to remove the DMF. The organic layer was dried over anhydrous Na_2_SO_4_, filtered, and evaporated under reduced pressure to yield the desired product. If needed, the compound was then purified by column chromatography as described in each specific case.

#### General Procedure D for the Synthesis of **81** and **100**

The corresponding 4-aryl-1*H*-pyrrole-3-carboxylate methyl ester (1.0 equiv) was dissolved in anhydrous DMF (1.0 mL·mmol^–1^) under an argon atmosphere. At 0 °C, NaH (60% dispersion, 1.3 equiv), dissolved in anhydrous DMF (1.0 mL·mmol^–1^), was added dropwise. Then, CH_3_I (2.0 equiv) was added dropwise, and the reaction mixture was stirred at r.t. for 2 h. Subsequently, 2 mL of H_2_O were added, the mixture was diluted with EtOAc (5 mL·mmol^–1^), and washed vigorously with a 1:1 mixture of H_2_O and saturated NaCl solution (5 × 5 mL·mmol^–1^) to remove DMF. The organic phase was dried over anhydrous Na_2_SO_4_, filtered, and evaporated under reduced pressure. The resulting compound was purified by column chromatography (Hexane/EtOAc, 7:3) to afford the corresponding 4-aryl-1-methyl-1*H*-pyrrole-3-carboxylate methyl ester.

#### General Procedure E for the Synthesis of **3**, **40–52**, **82**, and **101**

A mixture of the corresponding 4-aryl-1*H*-pyrrole-3-carboxylate methyl ester (1.0 equiv) and NaOH (10 equiv) was dissolved in a 1:1 MeOH/H_2_O solution (1.0 mL·mmol^–1^). The reaction mixture was heated to reflux for 1–2 h. After this time, the mixture was diluted with H_2_O (5 mL·mmol^–1^) and washed with EtOAc (1 × 1 mL·mmol^–1^). The aqueous layer was then acidified with 37% HCl to pH 2–3, or until precipitation occurred. The resulting solid was filtered to afford the corresponding 4-aryl-1*H*-pyrrole-3-carboxylic acid.

#### General Procedure F for the Synthesis of **71–75**

A mixture of the corresponding aryl aldehyde (1.0 equiv), diethyl malonate (1.5 equiv), pyrrolidine (30% mol) and triethylamine (1.0 equiv) was dissolved in anhydrous DCM containing 3Å molecular sieves in excess, previously activated by heating in an oven, under an argon atmosphere. The reaction mixture was heated at 130 °C under stirring and microwave irradiation for 2 h or until complete consumption of the limiting reagent, as monitored by TLC. Subsequently, the mixture was diluted with DCM (5 mL·mmol^–1^), filtered and washed vigorously with a saturated NH_4_ solution until acid pH (3 × 5 mL·mmol^–1^) to remove amines. The organic phase was evaporated under reduced pressure and the intermediate was used as obtained without further purification. Then, a mixture of TosMIC (1.1 equiv), the previous crude and NaOH (5 equiv) was dissolved in anhydrous ethanol (3 mL·mmol^–1^). The reaction mixture was stirred at r.t. for 1 h or until complete consumption of the limiting reagent, as monitored by TLC. Subsequently, the solvent is evaporated under reduced pressure, and the crude reaction mixture was dissolved with H_2_O and EtOAc (5 mL·mmol^–1^ each). The organic layer was washed vigorously with a saturated NaHCO_3_ solution (3 × 5 mL·mmol^–1^) to remove impurities. The organic layer was dried and evaporated under reduced pressure to yield the crude desired ester. Finally, a mixture of the former crude and NaOH (10 equiv) was dissolved in a 1:1 EtOH/H_2_O solution (1.0 mL·mmol^–1^). The reaction mixture was heated to reflux for 1–2 h. After this time, the mixture was diluted with H_2_O (5 mL·mmol^–1^) and washed with EtOAc (3 × 5 mL·mmol^–1^). The aqueous layer was then acidified with 37% HCl to pH 2–3, or until precipitation occurred. The resulting solid was filtered to afford the corresponding 4-aryl-1*H*-pyrrole-3-carboxylic acid.

#### General Procedure G for the Synthesis of **86–90**

To a mixture of the corresponding nitro derivative (1.0 equiv) and ethyl diazoacetate (4.0 equiv) at r.t., TEA (0.2 equiv) was added, and the reaction mixture was stirred for 24 h. The solvent and volatile components were then removed under reduced pressure. The resulting solid was washed with a DCM/Hexane mixture (8:2), dissolved in 37% HCl (3 mL·mmol^–1^), and heated under reflux for 4 h. After cooling, the mixture was concentrated by solvent evaporation. The solid was filtered and dried to obtain the corresponding acid.

#### General Procedure H for the Synthesis of **4** and **5**

A mixture of the corresponding 4-aryl-1*H*-pyrrole-3-carboxylic acid (1.0 equiv), the appropriate amine (1.2 equiv), EDC (1.0 equiv), DMAP (2.0 equiv), HOBt (0.1 equiv), and DIPEA (5 equiv) was dissolved in anhydrous CH_3_CN (2 mL·mmol^–1^) under an argon atmosphere. The reaction mixture was stirred at rt for 2 h or until complete consumption of the limiting reagent, as monitored by TLC. The solvent was removed under reduced pressure, and the crude product was dissolved in EtOAc (5 mL·mmol^–1^) and washed with H_2_O (3 × 5 mL·mmol^–1^). The organic phase was dried over anhydrous Na_2_SO_4_, filtered, and evaporated under reduced pressure. The crude product was purified by column chromatography (CH_2_Cl_2_/MeOH, 30:1).

#### General Procedure I for the Synthesis of **H3**, **53–68**, **76–80**, **83**, **84**, **91–97**, **102**

A mixture of the corresponding acid (1.0 equiv), the indicated amine (1.1 equiv), and BOP (1.3 equiv) was dissolved in anhydrous THF (5 mL·mmol^–1^) under an argon atmosphere. After complete dissolution, DIPEA (1.5 equiv) was added dropwise, and the reaction mixture was stirred at r.t. for 2 h or until complete consumption of the limiting reagent, as monitored by TLC. The THF was evaporated under reduced pressure, and the crude product was dissolved in EtOAc (5 mL·mmol^–1^) and washed with H_2_O (3 × 5 mL·mmol^–1^). The organic phase was then dried over anhydrous Na_2_SO_4_, filtered, and evaporated under reduced pressure. The crude product was purified by column chromatography (DCM/MeOH, 30:1).

##### *N*-(1*H*-Indazol-5-yl)-4-phenyl-1*H*-pyrrole-3-carboxamide (**H3**)

The title compound was prepared by reaction of 4-phenyl-1*H*-pyrrole-3-carboxylic acid (**3**) (365 mg, 1.95 mmol), 1*H*-indazol-5-amine (286 mg, 2.15 mmol), BOP (1121 mg, 2.54 mmol) and DIPEA (510 μL, 2.93 mmol) according to the procedure I. Compound **H3** was obtained as a white solid after chromatography column. Yield: 248 mg (42%). M.p.: 221–223 °C. ^1^H NMR (300 MHz, DMSO-*d*_6_) δ 12.92 (s, 1H), 11.37 (s, 1H), 9.68 (s, 1H), 8.15 (s, 1H), 8.00 (s, 1H), 7.56–7.42 (m, 5H), 7.11 (t, *J* = 9.0 Hz, 2H), 7.01 (t, *J* = 2.3 Hz, 1H). ^13^C NMR (75 MHz, DMSO-*d*_6_) δ 163.8, 160.6 (d, *J* = 241.6 Hz), 136.7, 133.3, 132.8, 132.0 (d, *J* = 3.1 Hz), 129.9 (d, *J* = 7.8 Hz), 123.0, 122.7, 121.9, 121.0, 118.2, 117.4, 114.4 (d, *J* = 21.0 Hz), 110.0, 109.8. HPLC-MS [M + H]^+^ = 321, Rt = 2.90 (99%). HRMS (ESI) *m*/*z* calcd. for C_18_H_13_FN_4_ONa [M + Na]^+^ 343.0966, found 343.0961.

##### *N*-(1*H*-Indol-5-yl)-4-phenyl-1*H*-pyrrole-3-carboxamide (**4**)

The title compound was prepared by reaction of 4-phenyl-1*H*-pyrrole-3-carboxylic acid (**3**) (150 mg, 0.80 mmol), 1*H*-indazol-5-amine (117 mg, 0.88 mmol), EDC (184 mg, 0.96 mmol), HOBt (10 mg, 0.08 mmol), DMAP (117 mg, 0.96 mmol) and DIPEA (689 μL, 4.00 mmol) according to the procedure H. Compound **4** was obtained as a white solid after chromatography column. Yield: 42 mg (17%). M.p.: 226–227 °C. ^1^H NMR (300 MHz, Acetone-*d*_6_) δ 10.61 (s, 1H), 10.15 (s, 1H), 8.21 (s, 1H), 7.93 (d, *J* = 1.9 Hz, 1H), 7.56 (dt, *J* = 8.2, 1.7 Hz, 2H), 7.48 (dd, *J* = 3.0, 2.2 Hz, 1H), 7.38 (t, *J* = 7.3 Hz, 2H), 7.33–7.24 (m, 3H), 7.12 (dd, *J* = 8.7, 2.0 Hz, 1H), 6.95 (t, *J* = 2.4 Hz, 1H), 6.39 (td, *J* = 2.1, 1.0 Hz, 1H). ^13^C NMR (75 MHz, Acetone-*d*_6_) δ 163.9, 136.7, 134.0, 132.8, 129.9, 129.1, 129.0, 127.1, 126.2, 124.7, 123.2, 119.8, 118.9, 116.0, 111.8, 111.7, 102.3. HPLC-MS [M + H]^+^ = 302, Rt = 2.96 (97%). HRMS (ESI) *m*/*z* calcd. for C_19_H_15_N_3_ONa [M + Na]^+^ 324.1113, found 324.1102.

##### *N*-(1*H*-Benzo­[*d*]­imidazol-5-yl)-4-phenyl-1H-pyrrole-3-carboxamide (**5**)

The title compound was prepared by reaction of 4-phenyl-1*H*-pyrrole-3-carboxylic acid (**3**) (150 mg, 0.80 mmol), 1*H*-indazol-5-amine (117 mg, 0.88 mmol), EDC (184 mg, 0.96 mmol), HOBt (10 mg, 0.08 mmol), DMAP (117 mg, 0.96 mmol) and DIPEA (689 μL, 4.00 mmol) according to the procedure H. Compound **5** was obtained as a yellow solid after chromatography column. Yield: 11 mg (5%). M.p.: 132–134 °C. ^1^H NMR (500 MHz, Methanol-*d*_4_) δ 7.98 (s, 1H), 7.52 (d, *J* = 1.8 Hz, 1H), 7.50 (d, *J* = 2.1 Hz, 1H), 7.32 (d, *J* = 8.5 Hz, 1H), 7.26 (d, *J* = 7.0 Hz, 2H), 7.17 (t, *J* = 7.4 Hz, 2H), 7.14–7.07 (m, 1H), 7.06 (d, *J* = 2.1 Hz, 1H), 6.78 (dd, *J* = 8.5, 2.2 Hz, 1H). ^13^C NMR (125 MHz, Methanol-*d*_4_) δ 165.7, 147.7, 143.0, 136.9, 135.7, 134.4, 129.5, 128.9, 127.4, 127.4, 127.2, 120.4, 119.9, 116.1, 115.3, 101.6. HPLC-MS [M + H]^+^ = 303, Rt = 2.35 (97%). HRMS (ESI) *m*/*z* calcd. for C_18_H_14_N_4_ONa [M + Na]^+^ 325.1060, found 325.1054.

##### 1*H*-Indazol-5-yl 4-phenyl-1*H*-pyrrole-3-carboxylate (**6**)

A mixture of 4-phenyl-1*H*-pyrrole-3-carboxylic acid (**3**) (150 mg, 0.80 mmol), 1*H*-indazol-5-ol (143 mg, 0.88 mmol), CDI (143 mg, 0.88 mmol), and DMAP (108 mg, 0.88) was dissolved in anhydrous CH_3_CN (3 mL·mmol^–1^) under an argon atmosphere. The reaction mixture was stirred at r.t. for 48 h. The solvent was evaporated under reduced pressure, and the crude product was dissolved in EtOAc (5 mL·mmol^–1^) and washed with H_2_O (3 × 5 mL·mmol^–1^). The organic layer was dried over anhydrous Na_2_SO_4_, filtered, and evaporated under reduced pressure. The final product was purified by column chromatography (CH_2_Cl_2_/MeOH, 30:1) as a white solid. Yield: 6 mg (3%). M.p.: 221–223 °C. ^1^H NMR (500 MHz, DMSO-*d*_6_) δ 11.75 (s, 1H), 8.05 (s, 1H), 7.77 (t, *J* = 2.7 Hz, 1H), 7.55–7.49 (m, 4H), 7.30 (t, *J* = 7.6 Hz, 2H), 7.20 (t, *J* = 7.4 Hz, 1H), 7.14 (dd, *J* = 8.9, 2.2 Hz, 1H), 7.04 (t, *J* = 2.4 Hz, 1H). ^13^C NMR (125 MHz, DMSO-*d*_6_) δ 163.1, 144.3, 137.7, 134.8, 133.3, 128.8, 127.6, 127.1, 126.0, 125.7, 122.7, 121.7, 119.5, 112.2, 110.9, 110.5. HPLC-MS [M + H]^+^ = 304, Rt = 3.03 (99%). HRMS (ESI) *m*/*z* calcd. for C_18_H_13_N_3_O_2_Na [M + Na]^+^ 326.0900, found 326.0893.

##### N-(1*H*-Indazol-5-yl)-4-(naphthalen-1-yl)-1*H*-pyrrole-3-carboxamide (**11**)

The title compound was prepared by reaction of (*E*)-*N*-(1*H*-indazol-5-yl)-3-(naphthalen-1-yl)­acrylamide (**8**) (400 mg, 1.28 mmol), TosMIC (275 mg, 1.41 mmol), and NaH (154 mg, 3.84 mmol) according to the procedure C. Compound **11** was obtained as a white solid after chromatography column (Hexane/EtOAc, 8:2). Yield: 30 mg (7%). M.p.: 197–199 °C. ^1^H NMR (300 MHz, Acetone-*d*_6_) δ 12.02 (s, 1H), 10.83 (s, 1H), 8.06–7.96 (m, 2H), 7.93 (d, *J* = 8.3 Hz, 1H), 7.85 (s, 1H), 7.80 (d, *J* = 1.3 Hz, 1H), 7.71 (dd, *J* = 3.2, 2.2 Hz, 1H), 7.69–7.60 (m, 3H), 7.55–7.40 (m, 2H), 7.26 (d, *J* = 8.9 Hz, 1H), 6.99 (t, *J* = 2.4 Hz, 1H), 6.60 (dd, *J* = 8.9, 2.0 Hz, 1H). ^13^C NMR (75 MHz, Acetone-*d*_6_) δ 163.2, 138.0, 134.8, 134.3, 134.3, 134.2, 133.3, 129.4, 129.1, 128.8, 127.1, 127.1, 126.9, 126.4, 124.1, 123.9, 121.5, 120.8, 120.8, 120.3, 110.6, 110.2. HPLC-MS [M + H]^+^ = 353, Rt = 3.02 (99%). HRMS (ESI) *m*/*z* calcd. for C_22_H_16_N_4_ONa [M + Na]^+^ 375.1216, found 375.1218.

##### 4-(3-Chlorophenyl)-*N*-(1*H*-indazol-5-yl)-1*H*-pyrrole-3-carboxamide (**12**)

The title compound was prepared by reaction of *tert*-butyl (*E*)-5-(3-(3-chlorophenyl)­acrylamido)-1*H*-indazole-1-carboxylate (**9**) (310 mg, 0.78 mmol), TosMIC (169 mg, 0.86 mmol), and NaH (94 mg, 2.34 mmol) according to the procedure C. Compound **12** was obtained as a brown solid after chromatography column (CH_2_Cl_2_/MeOH, 30:1). Yield: 7 mg (3%). M.p.: 196–198 °C. ^1^H NMR (500 MHz, DMSO-*d*_6_) δ 12.93 (s, 1H), 11.46 (s, 1H), 9.74 (s, 1H), 8.15 (s, 1H), 8.00 (s, 1H), 7.58 (t, *J* = 1.8 Hz, 1H), 7.52 (dd, *J* = 8.9, 1.5 Hz, 1H), 7.49 (t, *J* = 2.4 Hz, 1H), 7.48–7.43 (m, 2H), 7.31 (t, *J* = 7.8 Hz, 1H), 7.22 (ddd, *J* = 8.0, 2.0, 1.0 Hz, 1H), 7.13 (t, *J* = 2.3 Hz, 1H). ^13^C NMR (125 MHz, DMSO-*d*_6_) δ 163.7, 137.8, 136.8, 133.3, 132.7, 132.4, 129.5, 127.6, 126.6, 125.1, 122.7, 122.4, 122.2, 121.1, 118.9, 117.5, 110.1, 109.8. HPLC-MS [M + H]^+^ = 337, Rt = 2.86 (98%). HRMS (ESI) *m*/*z* calcd. for C_18_H_13_ClN_4_ONa [M + Na]^+^ 359.0670, found 359.0666.

##### 4-([1,1′-Biphenyl]-4-yl)-*N*-(1*H*-indazol-5-yl)-1*H*-pyrrole-3-carboxamide (**13**)

The title compound was prepared by reaction of *tert*-butyl (*E*)-5-(3-([1,1′-biphenyl]-4-yl)­acrylamido)-1*H*-indazole-1-carboxylate (**10**) (440 mg, 1.00 mmol), TosMIC (215 mg, 1.10 mmol), and NaH (120 mg, 3.00 mmol) according to the procedure C. Compound **13** was obtained as a brown solid after chromatography column (CH_2_Cl_2_/MeOH, 80:1). Yield: 26 mg (7%). M.p.: 213 °C d. ^1^H NMR (300 MHz, DMSO-*d*_6_) δ 12.93 (s, 1H), 11.39 (s, 1H), 9.74 (s, 1H), 8.18 (s, 1H), 8.00 (s, 1H), 7.70–7.64 (m, 2H), 7.62–7.57 (m, 4H), 7.53 (dd, *J* = 9.0, 1.9 Hz, 1H), 7.49–7.42 (m, 4H), 7.37–7.30 (m, 1H), 7.09 (t, *J* = 2.4 Hz, 1H). ^13^C NMR (125 MHz, DMSO-*d*_6_) δ 163.9, 140.1, 137.1, 136.7, 134.8, 133.3, 132.8, 128.9, 128.5, 127.1, 126.3, 126.0, 123.4, 122.7, 121.9, 121.0, 118.3, 117.7, 109.9, 109.8. HPLC-MS [M + H]^+^ = 379, Rt = 3.12 (97%). HRMS (ESI) *m*/*z* calcd. for C_24_H_18_ClN_4_ONa [M + Na]^+^ 401.1373, found 401.1369.

##### 4-(4-Fluorophenyl)-*N*-(1*H*-indazol-5-yl)-1*H*-pyrrole-3-carboxamide (**53**)

The title compound was prepared by reaction of 4-(4-fluorophenyl)-1*H*-pyrrole-3-carboxylic acid (**40**) (400 mg, 1.95 mmol), 1*H*-indazol-5-amine (286 mg, 2.15 mmol), BOP (1121 mg, 2.54 mmol) and DIPEA (510 μL, 2.93 mmol) according to the procedure I. Compound **53** was obtained as a gray solid after chromatography column. Yield: 475 mg (76%). M.p.: 221–223 °C. ^1^H NMR (300 MHz, DMSO-*d*_6_) δ 12.92 (s, 1H), 11.37 (s, 1H), 9.68 (s, 1H), 8.15 (s, 1H), 8.00 (s, 1H), 7.56–7.42 (m, 5H), 7.11 (t, *J* = 9.0 Hz, 2H), 7.01 (t, *J* = 2.3 Hz, 1H). ^13^C NMR (75 MHz, DMSO-*d*_6_) δ 163.8, 160.6 (d, *J* = 241.6 Hz), 136.7, 133.3, 132.8, 132.0 (d, *J* = 3.1 Hz), 129.9 (d, *J* = 7.8 Hz), 123.0, 122.7, 121.9, 121.0, 118.2, 117.4, 114.4 (d, *J* = 21.0 Hz), 110.0, 109.8. HPLC-MS [M + H]^+^ = 321, Rt = 2.90 (99%). HRMS (ESI) *m*/*z* calcd. for C_18_H_13_FN_4_ONa [M + Na]^+^ 343.0966, found 343.0961.

##### 4-(2-Chlorophenyl)-*N*-(1*H*-indazol-5-yl)-1*H*-pyrrole-3-carboxamide (**54**)

The title compound was prepared by reaction of 4-(2-chlorophenyl)-1*H*-pyrrole-3-carboxylic acid (**41**) (500 mg, 2.26 mmol), 1*H*-indazol-5-amine (331 mg, 2.49 mmol), BOP (1300 mg, 2.94 mmol) and DIPEA (609 μL, 3.40 mmol) according to the procedure I. Compound **54** was obtained as a white solid after chromatography column. Yield: 275 mg (36%). M.p.: 177–179 °C. ^1^H NMR (300 MHz, DMSO-*d*_6_) δ 12.90 (s, 1H), 11.39 (s, 1H), 9.50 (s, 1H), 8.08 (s, 1H), 7.96 (s, 1H), 7.55 (dd, *J* = 2.9, 2.1 Hz, 1H), 7.47 (dd, *J* = 9.0, 1.7 Hz, 1H), 7.43–7.39 (m, 2H), 7.38–7.33 (m, 1H), 7.32–7.22 (m, 2H), 6.88 (t, *J* = 2.3 Hz, 1H). ^13^C NMR (75 MHz, DMSO-*d*_6_) δ 163.0, 136.7, 135.2, 133.2, 133.2, 132.8, 132.1, 128.9, 127.7, 126.4, 122.7, 121.3, 121.0, 120.7, 119.2, 118.7, 109.9, 109.7. HPLC-MS [M + H]^+^ = 337, Rt = 2.48 (99%). HRMS (ESI) *m*/*z* calcd. for C_18_H_13_ClN_4_ONa [M + Na]^+^ 359.0670, found 359.0667.

##### 4-(4-Chlorophenyl)-*N*-(1*H*-indazol-5-yl)-1*H*-pyrrole-3-carboxamide (**55**)

The title compound was prepared by reaction of 4-(4-chlorophenyl)-1*H*-pyrrole-3-carboxylic acid (**42**) (500 mg, 2.26 mmol), 1*H*-indazol-5-amine (331 mg, 2.49 mmol), BOP (1300 mg, 2.94 mmol) and DIPEA (609 μL, 3.40 mmol) according to the procedure I. Compound **55** was obtained as a white solid after chromatography column. Yield: 210 mg (28%). M.p.: 219–221 °C. ^1^H NMR (300 MHz, DMSO-*d*_6_) δ 12.93 (s, 1H), 11.42 (s, 1H), 9.73 (s, 1H), 8.16 (s, 1H), 8.00 (s, 1H), 7.58–7.42 (m, 5H), 7.33 (d, *J* = 8.7 Hz, 2H), 7.07 (t, *J* = 2.3 Hz, 1H). ^13^C NMR (75 MHz, DMSO-*d*_6_) δ 163.7, 136.8, 134.5, 133.3, 132.7, 130.0, 129.7, 127.7, 122.7, 122.7, 122.1, 121.0, 118.5, 117.5, 110.0, 109.8. HPLC-MS [M + H]^+^ = 337, Rt = 3.02 (99%). HRMS (ESI) *m*/*z* calcd. for C_18_H_13_ClN_4_ONa [M + Na]^+^ 359.0670, found 359.0664.

##### 4-(2-Bromophenyl)-*N*-(1*H*-indazol-5-yl)-1*H*-pyrrole-3-carboxamide (**56**)

The title compound was prepared by reaction of 4-(2-bromophenyl)-1*H*-pyrrole-3-carboxylic acid (**43**) (200 mg, 0.75 mmol), 1*H*-indazol-5-amine (110 mg, 0.82 mmol), BOP (432 mg, 0.98 mmol) and DIPEA (197 μL, 1.13 mmol) according to the procedure I. Compound **56** was obtained as a brown solid after chromatography column. Yield: 43 mg (15%). M.p.: 124–126 °C. ^1^H NMR (500 MHz, DMSO-*d*_6_) δ 12.91 (s, 1H), 11.39 (s, 1H), 9.45 (s, 1H), 8.08 (s, 1H), 7.96 (s, 1H), 7.61 (d, *J* = 8.0 Hz, 1H), 7.58 (t, *J* = 2.4 Hz, 1H), 7.47 (dd, *J* = 8.8, 1.5 Hz, 1H), 7.43 (d, *J* = 8.8 Hz, 1H), 7.37–7.32 (m, 2H), 7.20 (ddd, *J* = 8.9, 6.3, 2.8 Hz, 1H), 6.87 (t, *J* = 2.2 Hz, 1H). ^13^C NMR (125 MHz, DMSO-*d*_6_) δ 162.8, 137.2, 136.6, 133.2, 132.7, 132.2, 132.0, 127.9, 126.8, 124.3, 123.2, 122.7, 121.0, 120.6, 119.0, 118.4, 109.9, 109.7. HPLC-MS [M + H]^+^ = 381, Rt = 2.78 (96%). HRMS (ESI) *m*/*z* calcd. for C_18_H_13_BrN_4_ONa [M + Na]^+^ 403.0165, found 403.0153.

##### 4-(3-Bromophenyl)-*N*-(1*H*-indazol-5-yl)-1*H*-pyrrole-3-carboxamide (**57**)

The title compound was prepared by reaction of 4-(3-bromophenyl)-1*H*-pyrrole-3-carboxylic acid (**44**) (200 mg, 0.75 mmol), 1*H*-indazol-5-amine (110 mg, 0.82 mmol), BOP (432 mg, 0.98 mmol) and DIPEA (197 μL, 1.13 mmol) according to the procedure I. Compound **57** was obtained as a brown solid after chromatography column. Yield: 55 mg (19%). M.p.: 99–101 °C. ^1^H NMR (300 MHz, Acetone-*d*_6_) δ 12.12 (s, 1H), 10.68 (s, 1H), 8.84 (s, 1H), 8.25 (s, 1H), 7.99 (d, *J* = 0.7 Hz, 1H), 7.79 (t, *J* = 1.8 Hz, 1H), 7.59–7.47 (m, 4H), 7.39 (ddd, *J* = 8.0, 2.0, 1.1 Hz, 1H), 7.27 (t, *J* = 7.8 Hz, 1H), 7.09 (t, *J* = 2.4 Hz, 1H). ^13^C NMR (75 MHz, Acetone-*d*_6_) δ 164.3, 139.2, 138.3, 134.5, 134.0, 132.2, 130.6, 129.5, 128.4, 124.3, 123.9, 123.1, 122.4, 121.7, 119.7, 119.4, 111.0, 110.6. HPLC-MS [M + H]^+^ = 381, Rt = 3.04 (95%). HRMS (ESI) *m*/*z* calcd. for C_18_H_13_BrN_4_ONa [M + Na]^+^ 403.0165, found 403.0158.

##### 4-(4-Bromophenyl)-*N*-(1*H*-indazol-5-yl)-1*H*-pyrrole-3-carboxamide (**58**)

The title compound was prepared by reaction of 4-(4-bromophenyl)-1*H*-pyrrole-3-carboxylic acid (**45**) (200 mg, 0.75 mmol), 1*H*-indazol-5-amine (110 mg, 0.82 mmol), BOP (432 mg, 0.98 mmol) and DIPEA (197 μL, 1.13 mmol) according to the procedure I. Compound **58** was obtained as a brown solid after chromatography column. Yield: 49 mg (17%). M.p.: 224–226 °C. ^1^H NMR (300 MHz, DMSO-*d*_6_) δ 12.93 (s, 1H), 11.42 (s, 1H), 9.74 (s, 1H), 8.15 (s, 1H), 8.00 (s, 1H), 7.52 (dd, *J* = 8.9, 1.8 Hz, 1H), 7.49–7.41 (m, 6H), 7.07 (t, *J* = 2.3 Hz, 1H). ^13^C NMR (125 MHz, DMSO-*d*_6_) δ 163.7, 136.7, 134.8, 133.3, 132.7, 130.6, 130.1, 122.7, 122.1, 122.1, 121.0, 118.5, 118.4, 117.5, 110.0, 109.8. HPLC-MS [M + H]^+^ = 381, Rt = 3.05 (95%). HRMS (ESI) *m*/*z* calcd. for C_18_H_13_BrN_4_ONa [M + Na]^+^ 403.0165, found 403.0156.

##### *N*-(1*H*-Indazol-5-yl)-4-(*p*-tolyl)-1*H*-pyrrole-3-carboxamide (**59**)

The title compound was prepared by reaction of 4-(*p*-tolyl)-1*H*-pyrrole-3-carboxylic acid (**46**) (400 mg, 1.99 mmol), 1*H*-indazol-5-amine (291 mg, 2.18 mmol), BOP (1142 mg, 2.58 mmol) and DIPEA (520 μL, 2.99 mmol) according to the procedure I. Compound **59** was obtained as a white solid after chromatography column. Yield: 144 mg (24%). M.p.: 229–231 °C. ^1^H NMR (300 MHz, DMSO-*d*_6_) δ 12.92 (s, 1H), 11.31 (s, 1H), 9.61 (s, 1H), 8.15 (s, 1H), 7.99 (s, 1H), 7.49 (dd, *J* = 9.0, 1.7 Hz, 1H), 7.45 (d, *J* = 8.6 Hz, 1H), 7.42 (t, *J* = 2.4 Hz, 1H), 7.37 (d, *J* = 8.1 Hz, 2H), 7.09 (d, *J* = 7.9 Hz, 2H), 6.97 (t, *J* = 2.3 Hz, 1H), 2.28 (s, 3H). ^13^C NMR (75 MHz, DMSO-*d*_6_) δ 163.9, 136.7, 134.4, 133.3, 132.8, 132.6, 128.4, 128.0, 123.8, 122.7, 121.7, 121.0, 117.8, 117.6, 109.8, 109.8, 20.7. HPLC-MS [M + H]^+^ = 317, Rt = 2.99 (99%). HRMS (ESI) *m*/*z* calcd. for C_19_H_16_N_4_ONa [M + Na]^+^ 339.1216, found 339.1214.

##### *N*-(1*H*-indazol-5-yl)-4-(4-isopropylphenyl)-1*H*-pyrrole-3-carboxamide (**60**)

The title compound was prepared by reaction of 4-(4-isopropylphenyl)-1*H*-pyrrole-3-carboxylic acid (**47**) (400 mg, 1.74 mmol), 1*H*-indazol-5-amine (254 mg, 1.91 mmol), BOP (1000 mg, 2.26 mmol) and DIPEA (455 μL, 2.61 mmol) according to the procedure I. Compound **60** was obtained as a red solid after chromatography column. Yield: 177 mg (30%). M.p.: 174 °C d. ^1^H NMR (300 MHz, DMSO-*d*_6_) δ 12.93 (s, 1H), 11.31 (s, 1H), 9.63 (s, 1H), 8.15 (s, 1H), 8.00 (s, 1H), 7.50 (dd, *J* = 9.0, 1.7 Hz, 1H), 7.45 (d, *J* = 9.0 Hz, 1H), 7.44 (t, *J* = 2.4 Hz, 1H), 7.39 (d, *J* = 8.2 Hz, 2H), 7.16 (d, *J* = 8.2 Hz, 2H), 6.96 (t, *J* = 2.3 Hz, 1H), 2.86 (hept, *J* = 6.7 Hz, 1H), 1.21 (d, *J* = 6.9 Hz, 6H). ^13^C NMR (75 MHz, DMSO-*d*_6_) δ 163.9, 145.5, 136.7, 133.3, 133.0, 132.8, 128.1, 125.7, 123.9, 122.7, 121.7, 121.0, 117.9, 117.6, 109.9, 109.8, 33.1, 24.0. HPLC-MS [M + H]^+^ = 345, Rt = 3.30 (95%). HRMS (ESI) *m*/*z* calcd. for C_21_H_20_N_4_ONa [M + Na]^+^ 367.1529, found 367.1523.

##### 4-(4-(*tert*-Butyl)­phenyl)-*N*-(1*H*-Indazol-5-yl)-1*H*-pyrrole-3-carboxamide (**61**)

The title compound was prepared by reaction 4-(4-(*tert*-butyl)­phenyl)-1*H*-pyrrole-3-carboxylic acid (**48**) (450 mg, 1.85 mmol), 1*H*-indazol-5-amine (271 mg, 2.03 mmol), BOP (1063 mg, 2.40 mmol) and DIPEA (482 μL, 2.77 mmol) according to the procedure I. Compound **61** was obtained as a white solid after chromatography column. Yield: 138 mg (21%). M.p.: 162–164 °C. ^1^H NMR (300 MHz, DMSO-*d*_6_) δ 12.92 (s, 1H), 11.31 (s, 1H), 9.63 (s, 1H), 8.15 (s, 1H), 7.99 (s, 1H), 7.50 (dd, *J* = 9.0, 1.8 Hz, 1H), 7.47–7.42 (m, 2H), 7.40 (d, *J* = 8.5 Hz, 2H), 7.30 (d, *J* = 8.6 Hz, 2H), 6.96 (t, *J* = 2.3 Hz, 1H), 1.29 (s, 9H). ^13^C NMR (75 MHz, DMSO-*d*_6_) δ 163.9, 147.7, 136.7, 133.3, 132.8, 132.6, 127.8, 124.5, 123.8, 122.7, 121.6, 121.0, 117.9, 117.6, 109.8, 109.8, 34.1, 31.2. HPLC-MS [M + H]^+^ = 359, Rt = 3.41 (99%). HRMS (ESI) *m*/*z* calcd. for C_22_H_22_N_4_ONa [M + Na]^+^ 381.1686, found 381.1677.

##### 4-(2,4-Dimethylphenyl)-*N*-(1*H*-indazol-5-yl)-1*H*-pyrrole-3-carboxamide (**62**)

The title compound was prepared by reaction 4-(2,4-dimethylphenyl)-1*H*-pyrrole-3-carboxylic acid (**49**) (400 mg, 1.86 mmol), 1*H*-indazol-5-amine (272 mg, 2.05 mmol), BOP (1010 mg, 2.42 mmol) and DIPEA (486 μL, 2.79 mmol) according to the procedure I. Compound **62** was obtained as a white solid after chromatography column. Yield: 105 mg (17%). M.p.: 183–185 °C. ^1^H NMR (300 MHz, DMSO-*d*_6_) δ 12.91 (s, 1H), 11.32 (s, 1H), 9.05 (s, 1H), 8.02 (s, 1H), 7.95 (s, 1H), 7.54 (t, *J* = 2.2 Hz, 1H), 7.41 (d, *J* = 8.9 Hz, 1H), 7.29 (dd, *J* = 8.9, 1.8 Hz, 1H), 7.07 (d, *J* = 7.6 Hz, 1H), 7.02 (s, 1H), 6.96 (d, *J* = 7.6 Hz, 1H), 6.72 (t, *J* = 2.2 Hz, 1H), 2.29 (s, 3H), 2.13 (s, 3H). ^13^C NMR (75 MHz, DMSO-*d*_6_) δ 163.1, 136.7, 136.5, 135.4, 133.2, 132.7, 132.6, 130.4, 130.1, 125.7, 123.0, 122.7, 121.2, 120.7, 118.3, 118.2, 109.8, 109.6, 20.7, 20.1. HPLC-MS [M + H]^+^ = 331, Rt = 3.15 (99%). HRMS (ESI) *m*/*z* calcd. for C_20_H_18_N_4_ONa [M + Na]^+^ 353.1373, found 353.1369.

##### *N*-(1*H*-Indazol-5-yl)-4-(4-nitrophenyl)-1*H*-pyrrole-3-carboxamide (**63**)

The title compound was prepared by reaction 4-(4-nitrophenyl)-1*H*-pyrrole-3-carboxylic acid (**50**) (128 mg, 0.55 mmol), 1*H*-indazol-5-amine (81 mg, 0.61 mmol), BOP (318 mg, 0.72 mmol) and DIPEA (145 μL, 0.83 mmol) according to the procedure I. Compound **63** was obtained as a yellow solid after chromatography column. Yield: 52 mg (27%). M.p.: 158–160 °C. ^1^H NMR (300 MHz, DMSO-*d*_6_) δ 12.94 (s, 1H), 11.64 (s, 1H), 9.89 (s, 1H), 8.18 (s, 1H), 8.15 (d, *J* = 9.1 Hz, 2H), 8.01 (s, 1H), 7.77 (d, *J* = 9.0 Hz, 2H), 7.58–7.52 (m, 2H), 7.47 (d, *J* = 8.9 Hz, 1H), 7.31 (t, *J* = 2.4 Hz, 1H). ^13^C NMR (75 MHz, DMSO-*d*_6_) δ 163.5, 144.9, 143.0, 136.8, 133.3, 132.6, 128.4, 123.1, 123.0, 122.7, 121.9, 121.1, 120.4, 117.9, 110.2, 109.8. HPLC-MS [M + H]^+^ = 348, Rt = 2.87 (99%). HRMS (ESI) *m*/*z* calcd. for C_18_H_13_N_5_O3Na [M + Na]^+^ 370.0911, found 370.0909.

##### *N*-(1*H*-Indazol-5-yl)-4-(pyridin-3-yl)-1*H*-pyrrole-3-carboxamide (**64**)

The title compound was prepared by reaction 4-(pyridine-3-yl)-1*H*-pyrrole-3-carboxylic acid (**51**) (113 mg, 0.60 mmol), 1*H*-indazol-5-amine (88 mg, 0.66 mmol), BOP (345 mg, 0.78 mmol) and DIPEA (157 μL, 0.90 mmol) according to the procedure I. Compound **64** was obtained as a brown solid after chromatography column. Yield: 71 mg (39%). M.p.: 170 °C d. ^1^H NMR (300 MHz, DMSO-*d*_6_) δ 12.94 (s, 1H), 11.52 (s, 1H), 9.76 (s, 1H), 8.68 (dd, *J* = 2.3, 0.9 Hz, 1H), 8.37 (dd, *J* = 4.8, 1.7 Hz, 1H), 8.15 (dd, *J* = 1.9, 0.8 Hz, 1H), 8.00 (d, *J* = 1.0 Hz, 1H), 7.88 (ddd, *J* = 7.9, 2.3, 1.7 Hz, 1H), 7.58 (dd, *J* = 3.0, 2.1 Hz, 1H), 7.53 (dd, *J* = 9.0, 1.9 Hz, 1H), 7.46 (d, *J* = 8.9 Hz, 1H), 7.31 (ddd, *J* = 7.9, 4.8, 0.9 Hz, 1H), 7.14 (t, *J* = 2.3 Hz, 1H). ^13^C NMR (75 MHz, DMSO-*d*_6_) δ 163.5, 148.7, 146.3, 136.8, 135.5, 133.3, 132.7, 131.4, 122.8, 122.7, 122.3, 121.1, 120.6, 118.9, 117.4, 110.2, 109.8. HPLC-MS [M + H]^+^ = 304, Rt = 1.91 (95%). HRMS (ESI) *m*/*z* calcd. for C_17_H_13_N_5_ONa [M + Na]^+^ 326.1012, found 326.1014.

##### *N*-(1*H*-Indazol-5-yl)-4-(pyridin-4-yl)-1*H*-pyrrole-3-carboxamide (**65**)

The title compound was prepared by reaction 4-(pyridine-4-yl)-1*H*-pyrrole-3-carboxylic acid (**52**) (113 mg, 0.60 mmol), 1*H*-indazol-5-amine (88 mg, 0.66 mmol), BOP (345 mg, 0.78 mmol) and DIPEA (157 μL, 0.90 mmol) according to the procedure I. Compound **65** was obtained as a brown solid after chromatography column. Yield: 56 mg (31%). M.p.: 267 °C d. ^1^H NMR (300 MHz, DMSO-*d*_6_) δ 13.07 (s, 1H), 11.88 (s, 1H), 9.98 (s, 1H), 8.46 (d, *J* = 6.3 Hz, 2H), 8.20 (s, 1H), 8.00 (s, 1H), 7.65–7.56 (m, 4H), 7.47 (d, *J* = 8.9 Hz, 1H), 7.37 (s, 1H). ^13^C NMR (75 MHz, DMSO-*d*_6_) δ 163.6, 147.7, 144.4, 136.9, 133.3, 132.7, 123.3, 122.7, 122.7, 121.2, 120.7, 120.7, 117.8, 110.2, 109.9. HPLC-MS [M + H]^+^ = 304, Rt = 1.87 (96%). HRMS (ESI) *m*/*z* calcd. for C_17_H_14_N_5_O [M + H]^+^ 304.1193, found 304.1196.

##### 4-(4-Chlorophenyl)-*N*-(6-methyl-1*H*-indazol-5-yl)-1*H*-pyrrole-3-carboxamide (**66**)

The title compound was prepared by reaction 4-(4-chlorophenyl)-1*H*-pyrrole-3-carboxylic acid (**42**) (133 mg, 0.60 mmol), 6-methyl1*H*-indazol-5-amine (97 mg, 0.66 mmol), BOP (345 mg, 0.78 mmol) and DIPEA (157 μL, 0.90 mmol) according to the procedure I. Compound **66** was obtained as a white solid after chromatography column. Yield: 46 mg (22%). M.p.: 210 °C d. ^1^H NMR (300 MHz, DMSO-*d*_6_) δ 12.87 (s, 1H), 11.41 (s, 1H), 9.10 (s, 1H), 7.97 (s, 1H), 7.72 (s, 1H), 7.59–7.43 (m, 3H), 7.43–7.26 (m, 3H), 7.04 (t, *J* = 2.4 Hz, 1H), 2.28 (s, 3H). ^13^C NMR (75 MHz, DMSO-*d*_6_) δ 164.1, 138.3, 134.5, 133.2, 133.0, 130.5, 130.1, 130.0, 127.6, 122.7, 122.2, 121.4, 118.6, 117.2, 117.0, 110.0, 18.7. HPLC-MS [M + H]^+^ = 351, Rt = 3.07 (99%). HRMS (ESI) *m*/*z* calcd. for C_19_H_16_ClN_4_O [M + H]^+^ 351.1007, found 351.1007.

##### *N*-(6-Chloro-1*H*-indazol-5-yl)-4-(4-chlorophenyl)-1*H*-pyrrole-3-carboxamide (**67**)

The title compound was prepared by reaction 4-(4-chlorophenyl)-1*H*-pyrrole-3-carboxylic acid (**42**) (133 mg, 0.60 mmol), 6-chloro-1*H*-indazol-5-amine (111 mg, 0.66 mmol), BOP (345 mg, 0.78 mmol) and DIPEA (157 μL, 0.90 mmol) according to the procedure I. Compound **67** was obtained as a white solid after chromatography column. Yield: 51 mg (23%). M.p.: 230 °C d. ^1^H NMR (500 MHz, DMSO-*d*_6_) δ 13.14 (s, 1H), 11.47 (s, 1H), 9.14 (s, 1H), 8.10 (s, 1H), 8.04 (s, 1H), 7.70 (s, 1H), 7.56 (t, *J* = 2.6 Hz, 1H), 7.52 (d, *J* = 8.5 Hz, 2H), 7.34 (d, *J* = 8.5 Hz, 2H), 7.05 (t, *J* = 2.4 Hz, 1H). ^13^C NMR (125 MHz, DMSO-*d*_6_) δ 163.8, 137.7, 134.2, 133.8, 130.3, 130.2, 128.4, 128.0, 127.7, 122.8, 122.7, 121.8, 118.9, 118.3, 116.6, 110.2. HPLC-MS [M + H]^+^ = 371, Rt = 3.27 (99%). HRMS (ESI) *m*/*z* calcd. for C_18_H_13_Cl_2_N_4_O [M + H]^+^ 371.0461, found 371.0465.

##### *N*-(4-Chloro-1*H*-indazol-5-yl)-4-(4-chlorophenyl)-1*H*-pyrrole-3-carboxamide (**68**)

The title compound was prepared by reaction 4-(4-chlorophenyl)-1*H*-pyrrole-3-carboxylic acid (**42**) (133 mg, 0.60 mmol), 4-chloro-1*H*-indazol-5-amine (111 mg, 0.66 mmol), BOP (345 mg, 0.78 mmol) and DIPEA (157 μL, 0.90 mmol) according to the procedure I. Compound **68** was obtained as a white solid after chromatography column. Yield: 97 mg (44%). M.p.: 286 °C d. ^1^H NMR (300 MHz, DMSO-*d*_6_) δ 13.38 (s, 1H), 11.47 (s, 1H), 9.29 (s, 1H), 8.10 (s, 1H), 7.70–7.44 (m, 5H), 7.33 (d, *J* = 8.2 Hz, 2H), 7.05 (t, *J* = 2.4 Hz, 1H). ^13^C NMR (75 MHz, DMSO-*d*_6_) δ 163.7, 138.6, 134.3, 132.0, 130.2, 130.1, 128.2, 127.7, 126.8, 122.9, 122.8, 122.2, 119.2, 118.9, 116.4, 108.9. HPLC-MS [M + H]^+^ = 371, Rt = 3.29 (95%). HRMS (ESI) *m*/*z* calcd. for C_18_H_12_Cl_2_N_4_ONa [M + Na]^+^ 393.0286, found 393.0281.

##### 4-(4-Chlorophenyl)-*N*-(4-methyl-1*H*-indazol-5-yl)-1H-pyrrole-3-carboxamide (**69**)

The title compound was prepared by reaction 4-(4-chlorophenyl)-1*H*-pyrrole-3-carboxylic acid (**42**) (133 mg, 0.60 mmol), 4-methyl-1*H*-indazol-5-amine (106 mg, 0.66 mmol), BOP (345 mg, 0.78 mmol) and DIPEA (157 μL, 0.90 mmol) according to the procedure I. Compound **69** was obtained as a white solid after chromatography column. Yield: 70 mg (33%). M.p.: 260 °C d. ^1^H NMR (300 MHz, DMSO-*d*_6_) δ 12.98 (s, 1H), 11.41 (s, 1H), 9.27 (s, 1H), 8.12 (d, J = 0.9 Hz, 1H), 7.57–7.50 (m, 3H), 7.39–7.20 (m, 4H), 7.06 (t, J = 2.4 Hz, 1H), 2.41 (s, 3H). 13C NMR (75 MHz, DMSO-*d*_6_) δ 164.4, 138.4, 135.0, 133.1, 130.4, 130.3, 128.9, 128.0, 126.8, 125.7, 124.4, 123.2, 122.6, 119.0, 117.5, 107.5, 14.8. HPLC-MS [M + H]^+^ = 371, Rt = 3.05 (97%). HRMS (ESI) *m*/*z* calcd. for C_19_H_15_ClN_4_ONa [M + Na]^+^ 373.0827, found 373.0828.

##### 4-(4-Chlorophenyl)-*N*-(6-hydroxy-1*H*-indazol-5-yl)-1H-pyrrole-3-carboxamide (**70**)

The title compound was prepared by reaction 4-(4-chlorophenyl)-1*H*-pyrrole-3-carboxylic acid (**42**) (133 mg, 0.60 mmol), 6-hidroxy-1*H*-indazol-5-amine (107 mg, 0.66 mmol), BOP (345 mg, 0.78 mmol) and DIPEA (157 μL, 0.90 mmol) according to the procedure I. Compound **70** was obtained as a white solid after chromatography column. Yield: 55 mg (26%). M.p.: 230 °C d. ^1^H NMR (300 MHz, DMSO-*d*_6_) δ 12.52 (s, 1H), 11.50 (s, 1H), 10.13 (s, 1H), 8.47 (s, 1H), 8.31 (s, 1H), 7.87 (s, 1H), 7.64 (s, 1H), 7.58–7.46 (m, 3H), 7.44–7.32 (m, 2H), 7.02 (t, J = 2.4 Hz, 1H). ^13^C NMR (75 MHz, DMSO-*d*_6_) δ 163.53, 148.32, 147.46, 134.47, 131.97, 131.09, 130.73, 128.49, 123.71, 123.21, 122.52, 119.48, 117.89, 117.15, 116.53. HPLC-MS [M + H]^+^ = 371, Rt = 2.92 (95%). HRMS (ESI) *m*/*z* calcd. for C_18_H_13_ClN_4_O2Na [M + Na]^+^ 375.0619, found 375.0623.

##### 4-(4-Methoxyphenyl)-*N*-(1*H*-indazol-5-yl)-1*H*-pyrrole-3-carboxamide (**76**)

The title compound was prepared by reaction of 4-(4-methoxyphenyl)-1*H*-pyrrole-3-carboxylic acid (**71**) (240 mg, 1.10 mmol), 1*H*-indazol-5-amine (162 mg, 1.22 mmol), BOP (637 mg, 1.44 mmol) and DIPEA (288 μL, 1.66 mmol) according to the procedure I. Compound **76** was obtained as a brown solid after chromatography column. Yield: 27 mg (12%). M.p.: 124 °C d. ^1^H NMR (300 MHz, DMSO) δ 12.93 (s, 1H), 11.29 (s, 1H), 9.58 (s, 1H), 8.19–8.12 (m, 1H), 8.00 (s, 1H), 7.52–7.38 (m, 5H), 6.93 (t, *J* = 2.3 Hz, 1H), 6.90–6.84 (m, 2H), 3.74 (s, 3H). ^13^C NMR (75 MHz, DMSO) δ 163.9, 157.5, 136.7, 133.3, 132.8, 129.3, 128.0, 123.6, 122.7, 121.7, 121.0, 117.5, 117.5, 113.3, 109.9, 109.8, 55.0. HPLC-MS [M + H]^+^ = 333, Rt = 3.07 (98%). HRMS (ESI) *m*/*z* calcd. for C_19_H_16_N_4_O_2_Na [M + Na]^+^ 355.1165, found 355.1165.

##### 4-(4-(Dimethylamino)­phenyl)-*N*-(1*H*-indazol-5-yl)-1*H*-pyrrole-3-carboxamide (**77**)

The title compound was prepared by reaction of 4-(4-(dimethylamino)­phenyl)-1*H*-pyrrole-3-carboxylic acid (**72**) (200 mg, 0.87 mmol), 1*H*-indazol-5-amine (128 mg, 0.96 mmol), BOP (500 mg, 1.13 mmol) and DIPEA (226 μL, 1.30 mmol) according to the procedure I. Compound **77** was obtained as a gray solid after chromatography column. Yield: 97 mg (32%). M.p.: 147 °C d. ^1^H NMR (300 MHz, DMSO-*d*_6_) δ 12.92 (s, 1H), 11.21 (s, 1H), 9.46 (s, 1H), 8.14 (d, *J* = 1.4 Hz, 1H), 7.99 (d, *J* = 1.4 Hz, 1H), 7.45 (d, *J* = 1.4 Hz, 2H), 7.38 (dd, *J* = 3.0, 2.2 Hz, 1H), 7.36–7.29 (m, 2H), 6.86 (t, *J* = 2.3 Hz, 1H), 6.72–6.66 (m, 2H), 2.87 (s, 6H). ^13^C NMR (75 MHz, DMSO-*d*_6_) δ 164.0, 148.8, 136.7, 133.3, 132.8, 128.9, 124.0, 123.7, 122.7, 121.5, 120.9, 117.5, 116.9, 112.2, 109.8, 109.7, 40.4. HPLC-MS [M + H]^+^ = 346, Rt = 2.21 (97%). HRMS (ESI) *m*/*z* calcd. for C_20_H_19_N_5_ONa [M + Na]^+^ 368.1482, found 368.1479.

##### 4-(4-Morpholinophenyl)-*N*-(1*H*-indazol-5-yl)-1*H*-pyrrole-3-carboxamide (**78**)

The title compound was prepared by reaction of 4-(4-morpholinophenyl)-1*H*-pyrrole-3-carboxylic acid (**73**) (250 mg, 0.92 mmol), 1*H*-indazol-5-amine (160 mg, 1.20 mmol), BOP (628 mg, 1.42 mmol) and DIPEA (285 μL, 1.64 mmol) according to the procedure I. Compound **78** was obtained as a gray solid after chromatography column. Yield: 161 mg (45%). M.p.: 249 °C d. ^1^H NMR (300 MHz, DMSO-*d*_6_) δ 12.92 (s, 1H), 11.25 (s, 1H), 9.55 (s, 1H), 8.15 (t, *J* = 1.3 Hz, 1H), 7.99 (s, 1H), 7.51–7.42 (m, 2H), 7.41–7.33 (m, 3H), 6.94–6.84 (m, 3H), 3.73 (dd, *J* = 6.1, 3.5 Hz, 4H), 3.08 (dd, *J* = 5.8, 3.9 Hz, 4H). ^13^C NMR (75 MHz, DMSO-*d*_6_) δ 164.0, 149.1, 136.7, 133.3, 132.8, 128.8, 126.6, 123.8, 122.7, 121.6, 120.9, 117.5, 117.3, 114.8, 109.8, 66.1, 48.7. HPLC-MS [M + H]^+^ = 388, Rt = 2.63 (97%). HRMS (ESI) *m*/*z* calcd. for C_22_H_21_N_5_O_2_Na [M + Na]^+^ 410.1587, found 410.1591.

##### *N*-(1*H*-indazol-5-yl)-4-(quinolin-4-yl)-1*H*-pyrrole-3-carboxamide (**79**)

The title compound was prepared by reaction of 4-(quinoline-4-yl)-1*H*-pyrrole-3-carboxylic acid (**74**) (275 mg, 1.15 mmol), 1*H*-indazol-5-amine (169 mg, 1.27 mmol), BOP (663 mg, 1.50 mmol) and DIPEA (301 μL, 1.73 mmol) according to the procedure I. Compound **79** was obtained as a beige solid after chromatography column. Yield: 82 mg (20%). M.p.: 309 °C d. ^1^H NMR (300 MHz, DMSO-*d*_6_) δ 13.01 (s, 1H), 11.67 (s, 1H), 9.87 (s, 1H), 9.09 (d, *J* = 2.2 Hz, 1H), 8.43 (dd, *J* = 2.3, 0.8 Hz, 1H), 8.26–8.19 (m, 1H), 8.09–8.02 (m, 2H), 8.02–7.94 (m, 1H), 7.79–7.74 (m, 1H), 7.73 (q, *J* = 1.9 Hz, 1H), 7.67–7.51 (m, 3H), 7.34 (t, *J* = 2.3 Hz, 1H). ^13^C NMR (75 MHz, DMSO-*d*_6_) δ 163.5, 151.7, 145.8, 136.8, 133.3, 132.7, 132.6, 129.0, 128.5, 128.4, 127.9, 127.7, 126.4, 122.7, 122.4, 121.2, 120.9, 119.5, 117.5, 110.3, 109.8. HPLC-MS [M + H]^+^ = 354, Rt = 2.23 (96%). HRMS (ESI) *m*/*z* calcd. for C_21_H_15_N_5_ONa [M + Na]^+^ 376.1169, found 376.1168.

##### *N*-(1*H*-indazol-5-yl)-4-(quinolin-3-yl)-1*H*-pyrrole-3-carboxamide (**80**)

The title compound was prepared by reaction of 4-(quinoline-3-yl)-1*H*-pyrrole-3-carboxylic acid (**75**) (125 mg, 0.52 mmol), 1*H*-indazol-5-amine (76 mg, 0.57 mmol), BOP (301 mg, 0.68 mmol) and DIPEA (136 μL, 0.78 mmol) according to the procedure I. Compound **80** was obtained as a white solid after chromatography column. Yield: 47 mg (23%). M.p.: 248 °C d. ^1^H NMR (300 MHz, DMSO-*d*_6_) δ 12.89 (s, 1H), 11.67 (s, 1H), 9.69 (s, 1H), 8.82 (d, *J* = 4.5 Hz, 1H), 8.06–7.89 (m, 4H), 7.77 (dd, *J* = 3.0, 2.1 Hz, 1H), 7.68 (m, 1H), 7.47 (m, 1H), 7.41 (d, *J* = 1.7 Hz, 2H), 7.37 (d, *J* = 4.5 Hz, 1H), 7.08 (t, *J* = 2.3 Hz, 1H). ^13^C NMR (75 MHz, DMSO-*d*_6_) δ 162.6, 149.7, 147.9, 143.1, 136.7, 133.2, 132.5, 129.1, 128.7, 127.8, 126.4, 125.8, 122.7, 122.0, 121.7, 121.1, 120.2, 119.7, 119.1, 110.1, 109.7. HPLC-MS [M + H]^+^ = 354, Rt = 2.04 (95%). HRMS (ESI) *m*/*z* calcd. for C_21_H_15_N_5_ONa [M + Na]^+^ 376.1169, found 376.1171.

##### 4-(4-Chlorophenyl)-*N*-(1*H*-indazol-5-yl)-1-methyl-1*H*-pyrrole-3-carboxamide (**83**)

The title compound was prepared by reaction 4-(4-chlorophenyl)-1-methyl-1*H*-pyrrole-3-carboxylic acid **(82)** (141 mg, 0.60 mmol), 1*H*-indazol-5-amine (88 mg, 0.66 mmol), BOP (345 mg, 0.78 mmol) and DIPEA (157 μL, 0.90 mmol) according to the procedure I. Compound **83** was obtained as a brown solid after chromatography column. Yield: 107 mg (51%). M.p.: 211–213 °C. ^1^H NMR (300 MHz, DMSO-*d*_6_) δ 12.94 (s, 1H), 9.75 (s, 1H), 8.15 (s, 1H), 8.00 (s, 1H), 7.54–7.41 (m, 5H), 7.37–7.30 (m, 2H), 7.04 (d, *J* = 2.3 Hz, 1H), 3.70 (s, 3H). ^13^C NMR (75 MHz, DMSO-*d*_6_) δ 163.3, 136.8, 134.1, 133.3, 132.7, 130.1, 129.6, 127.7, 125.6, 122.9, 122.7, 122.3, 120.9, 117.5, 109.9, 109.8, 36.1. HPLC-MS [M + H]^+^ = 351, Rt = 3.26 (95%). HRMS (ESI) *m*/*z* calcd. for C_19_H_15_ClN_4_ONa [M + Na]^+^ 373.0832 found, 373.0832.

##### *tert*-Butyl 5-(4-(4-Chlorophenyl)-1*H*-pyrrole-3-carboxamido)-1*H*-indazole-1-carboxylate (**84**)

The title compound was prepared by reaction 4-(4-chlorophenyl)-1*H*-pyrrole-3-carboxylic acid (**42**) (173 mg, 0.78 mmol), *tert*-butyl 5-amino-1*H*-indazole-1-carboxylate (200 mg, 0.86 mmol), BOP (448 mg, 1.01 mmol) and DIPEA (204 μL, 1.17 mmol) according to the procedure I. Compound **84** was obtained as a white solid after chromatography column. Yield: 151 mg (44%). ^1^H NMR (300 MHz, DMSO-*d*_6_) δ 11.48 (s, 1H), 9.96 (s, 1H), 8.38 (d, *J* = 0.8 Hz, 1H), 8.32 (d, *J* = 1.3 Hz, 1H), 7.99 (d, *J* = 9.0 Hz, 1H), 7.76 (dd, *J* = 9.1, 2.0 Hz, 1H), 7.53 (dd, *J* = 3.0, 2.1 Hz, 1H), 7.50 (d, *J* = 8.8 Hz, 2H), 7.33 (d, *J* = 8.8 Hz, 2H), 7.08 (t, *J* = 2.4 Hz, 1H), 1.65 (s, 9H). ^13^C NMR (75 MHz, DMSO-*d*_6_) δ 163.9, 148.5, 139.9, 135.9, 135.2, 134.4, 130.1, 129.8, 127.7, 125.9, 122.9, 122.6, 122.5, 118.7, 117.1, 113.9, 110.7, 84.3, 27.7. HPLC-MS [M + H]^+^ = 473, Rt = 3.76 (99%).

##### 4-(4-Chlorophenyl)-1-(3-(dimethylamino)­propyl)-*N*-(1H-indazol-5-yl)-1*H*-pyrrole-3-carboxamide **85**

A mixture of *tert*-butyl 5-(4-(4-chlorophenyl)-1*H*-pyrrole-3-carboxamido)-1*H*-indazole-1-carboxylate (**84**) (140 mg, 0.32 mmol), Cs_2_CO_3_ (135 mg, 0.42 mmol), 3-chloro-*N*,*N*-dimethylpropan-1-amine (55 μL, 0.42 mmol), and KI (10 mg, 0.06 mmol) was dissolved in anhydrous DMF (1 mL·mmol^–1^) and stirred at 80 °C for 18 h. EtOAc (5 mL·mmol^–1^) was added, and the mixture was washed with a 1:1 mixture of H_2_O and saturated NaHCO_3_ solution (5 × 5 mL·mmol^–1^) to remove the DMF. The organic phase was dried over anhydrous Na_2_SO_4_, filtered, and evaporated. The crude product was then dissolved in a mixture of DCM and TFA (4:1, 3 mL·mmol^–1^) and stirred at r.t. for 6 h. Saturated NaHCO_3_ solution was added until basic pH. The aqueous phase was extracted with EtOAc (3 × 5 mL·mmol^–1^), dried over anhydrous Na_2_SO_4_, and evaporated. The crude product was purified by column chromatography (DCM:MeOH, 9:1) to afford the desired compound **85** as a white solid. Yield: 21 mg (16%). M.p.: 224–226 °C. ^1^H NMR (300 MHz, DMSO-*d*_6_) δ 12.94 (s, 1H), 9.72 (s, 1H), 8.14 (s, 1H), 8.00 (s, 1H), 7.55–7.41 (m, 5H), 7.33 (d, *J* = 8.7 Hz, 2H), 7.10 (d, *J* = 2.2 Hz, 1H), 3.97 (t, *J* = 7.0 Hz, 2H), 2.33 (t, *J* = 6.8 Hz, 2H), 2.23 (s, 6H), 2.04–1.89 (m, 2H). ^13^C NMR (75 MHz, DMSO-*d*_6_) δ 163.3, 136.8, 134.1, 133.3, 132.7, 130.1, 129.6, 127.7, 124.7, 122.8, 122.7, 121.4, 121.0, 117.3, 110.0, 109.8, 55.6, 47.1, 44.8, 28.2. HPLC-MS [M + H]^+^ = 422, Rt = 2.45 (95%). HRMS (ESI) *m*/*z* calcd. for C_23_H_24_ClN_5_O [M + H]^+^ 422.1742, found 422.1735.

##### *N*-(1*H*-indazol-5-yl)-4-phenyl-1*H*-pyrazole-3-carboxamide (**91**)

The title compound was prepared by reaction 4-phenyl-1*H*-pyrazole-3-carboxylic acid (**86**) (113 mg, 0.60 mmol), 1*H*-indazol-5-amine (88 mg, 0.66 mmol), BOP (345 mg, 0.78 mmol) and DIPEA (157 μL, 0.90 mmol) according to the procedure I. Compound **91** was obtained as a white solid after chromatography column. Yield: 69 mg (38%). M.p.: 262–264 °C. ^1^H NMR (300 MHz, DMSO-*d*_6_) δ 13.45 (s, 1H), 12.98 (s, 1H), 10.20 (s, 1H), 8.27 (d, *J* = 1.8 Hz, 1H), 8.15 (s, 1H), 8.03 (s, 1H), 7.76–7.54 (m, 3H), 7.49 (d, *J* = 8.8 Hz, 1H), 7.35 (t, *J* = 7.6 Hz, 2H), 7.30–7.16 (m, 1H). ^13^C NMR (75 MHz, DMSO-*d*_6_) δ 161.6, 143.1, 137.0, 133.4, 132.3, 132.0, 129.4, 128.4, 128.0, 126.4, 122.7, 121.8, 121.1, 110.5, 109.9. HPLC-MS [M + H]^+^ = 304, Rt = 2.83 (95%). HRMS (ESI) *m*/*z* calcd. for C_17_H_13_N_5_ONa [M + Na]^+^ 326.1018, found 326.1011.

##### 4-(4-Fluorophenyl)-*N*-(1*H*-indazol-5-yl)-1*H*-pyrazole-3-carboxamide (**92**)

The title compound was prepared by reaction 4-(4-fluorophenyl)-1*H*-pyrazole-3-carboxylic acid (**87**) (124 mg, 0.60 mmol), 1*H*-indazol-5-amine (88 mg, 0.66 mmol), BOP (345 mg, 0.78 mmol) and DIPEA (157 μL, 0.90 mmol) according to the procedure I. Compound **92** was obtained as a white solid after chromatography column. Yield: 77 mg (40%). M.p.: 288–290 °C. ^1^H NMR (300 MHz, DMSO-*d*_6_) δ 13.48 (s, 1H), 12.98 (s, 1H), 10.19 (s, 1H), 8.27 (s, 1H), 8.15 (s, 1H), 8.03 (s, 1H), 7.75–7.58 (m, 3H), 7.49 (d, *J* = 8.8 Hz, 1H), 7.19 (t, *J* = 8.9 Hz, 2H). ^13^C NMR (75 MHz, DMSO-*d*_6_) δ 161.5, 161.1 (d, *J* = 243.1 Hz), 142.8, 137.0, 133.4, 131.9, 130.4 (d, *J* = 8.0 Hz), 129.6, 128.8 (d, *J* = 2.3 Hz), 122.7, 121.1, 120.9, 114.7 (d, *J* = 21.2 Hz), 110.6, 109.9. HPLC-MS [M + H]^+^ = 322, Rt = 2.91 (95%). HRMS (ESI) *m*/*z* calcd. for C_17_H_12_FN_5_ONa [M + Na]^+^ 344.0924, found 344.0921.

##### *N*-(1*H*-Indazol-5-yl)-4-(*p*-tolyl)-1*H*-pyrazole-3-carboxamide (**93**)

The title compound was prepared by reaction 4-(*p*-tolyl)-1*H*-pyrazole-3-carboxylic acid (**88**) (121 mg, 0.60 mmol), 1*H*-indazol-5-amine (88 mg, 0.66 mmol), BOP (345 mg, 0.78 mmol) and DIPEA (157 μL, 0.90 mmol) according to the procedure I. Compound **93** was obtained as a white solid after chromatography column. Yield: 70 mg (37%). M.p.: 276–278 °C. ^1^H NMR (300 MHz, DMSO-*d*_6_) δ 13.40 (s, 1H), 12.98 (s, 1H), 10.17 (s, 1H), 8.26 (s, 1H), 8.10 (s, 1H), 8.03 (s, 1H), 7.64 (d, *J* = 9.2 Hz, 1H), 7.55–7.46 (m, 3H), 7.15 (d, *J* = 7.9 Hz, 2H), 2.30 (s, 3H). ^13^C NMR (75 MHz, DMSO-*d*_6_) δ 161.7, 143.1, 137.0, 135.5, 133.4, 132.0, 129.4, 129.1, 128.6, 128.3, 122.7, 121.7, 121.1, 110.4, 109.9, 20.7. HPLC-MS [M + H]^+^ = 319, Rt = 3.00 (99%). HRMS (ESI) *m*/*z* calcd. for C_18_H_16_N_5_O [M + H]^+^ 318.1349, found 318.1353.

##### *N*-(1*H*-Indazol-5-yl)-4-(4-methoxyphenyl)-1*H*-pyrazole-3-carboxamide (**94**)

The title compound was prepared by reaction 4-(4-methoxyphenyl)-1*H*-pyrazole-3-carboxylic acid (**89**) (131 mg, 0.60 mmol), 1*H*-indazol-5-amine (88 mg, 0.66 mmol), BOP (345 mg, 0.78 mmol) and DIPEA (157 μL, 0.90 mmol) according to the procedure I. Compound **94** was obtained as a white solid after chromatography column. Yield: 70 mg (35%). M.p.: 275–277 °C. ^1^H NMR (300 MHz, DMSO-*d*_6_) δ 13.38 (s, 1H), 12.97 (s, 1H), 10.15 (s, 1H), 8.27 (s, 1H), 8.07 (s, 1H), 8.03 (s, 1H), 7.65 (dd, *J* = 9.0, 1.9 Hz, 1H), 7.56 (d, *J* = 8.7 Hz, 2H), 7.48 (d, *J* = 8.8 Hz, 1H), 6.92 (d, *J* = 8.8 Hz, 2H), 3.76 (s, 3H). ^13^C NMR (75 MHz, DMSO-*d*_6_) δ 161.7, 158.0, 142.8, 136.9, 133.4, 132.0, 129.6, 128.9, 124.7, 122.7, 121.6, 121.1, 113.4, 110.4, 109.9, 55.1. HPLC-MS [M + H]^+^ = 334, Rt = 2.84 (99%). HRMS (ESI) *m*/*z* calcd. for C_18_H_15_N_5_O_2_Na [M + Na]^+^ 356.1118, found 356.1117.

##### 4-(Furan-2-yl)-*N*-(1*H*-indazol-5-yl)-1*H*-pyrazole-3-carboxamide (**95**)

The title compound was prepared by reaction 4-(furan-2-yl)-1*H*-pyrazole-3-carboxylic acid (**90**) (107 mg, 0.60 mmol), 1*H*-indazol-5-amine (88 mg, 0.66 mmol), BOP (345 mg, 0.78 mmol) and DIPEA (157 μL, 0.90 mmol) according to the procedure I. Compound **95** was obtained as a white solid after chromatography column. Yield: 58 mg (33%). M.p.: 264–267 °C. ^1^H NMR (300 MHz, DMSO-*d*_6_) δ 13.61 (s, 1H), 12.99 (s, 1H), 10.16 (s, 1H), 8.29 (d, *J* = 1.8 Hz, 1H), 8.22 (s, 1H), 8.06 (s, 1H), 7.74–7.60 (m, 2H), 7.50 (d, *J* = 8.9 Hz, 1H), 7.12 (d, *J* = 3.3 Hz, 1H), 6.51 (dd, *J* = 3.3, 1.9 Hz, 1H). ^13^C NMR (75 MHz, DMSO-*d*_6_) δ 160.8, 146.8, 141.5, 141.4, 137.0, 133.5, 131.8, 128.2, 122.7, 121.3, 113.2, 111.4, 110.8, 109.9, 108.3. HPLC-MS [M + H]^+^ = 294, Rt = 2.79 (95%). HRMS (ESI) *m*/*z* calcd. for C_15_H_11_N_5_O_2_Na [M + Na]^+^ 316.0805, found 316.0803.

##### *N*-(1*H*-Indazol-5-yl)-5-methyl-3-phenylisoxazole-4-carboxamide (**96**)

The title compound was prepared by reaction 5-methyl-3-phenylisoxazole-4-carboxylic acid (300 mg, 1.48 mmol), 1*H*-indazol-5-amine (216 mg, 1.62 mmol), BOP (849 mg, 1.92 mmol) and DIPEA (387 μL, 2.22 mmol) according to the procedure I. Compound **96** was obtained as a white solid after chromatography column. Yield: 222 mg (47%). M.p.: 220–222 °C. ^1^H NMR (300 MHz, DMSO-*d*_6_) δ 13.03 (s, 1H), 10.44 (s, 1H), 8.18 (s, 1H), 8.06 (s, 1H), 7.76–7.69 (m, 2H), 7.54–7.41 (m, 5H), 2.60 (s, 3H). ^13^C NMR (75 MHz, DMSO-*d*_6_) δ 169.7, 160.2, 159.8, 137.2, 133.5, 131.5, 130.1, 128.9, 128.1, 127.7, 122.6, 120.6, 113.5, 110.6, 110.3, 11.9. HPLC-MS [M + H]^+^ = 319, Rt = 3.05 (99%). HRMS (ESI) *m*/*z* calcd. for C_18_H_14_N_4_O_2_ [M + H]^+^ 319.1190, found 319.1178.

##### *N*-(1*H*-Indazol-5-yl)-5-phenyloxazole-4-carboxamide (**97**)

The title compound was prepared by reaction 5-phenyloxazole-4-carboxylic acid (300 mg, 1.59 mmol), 1*H*-indazol-5-amine (232 mg, 1.74 mmol), BOP (912 mg, 1.06 mmol) and DIPEA (413 μL, 2.37 mmol) according to the procedure I. Compound **97** was obtained as a white solid after chromatography column. Yield: 154 mg (32%). M.p.: 228–230 °C. ^1^H NMR (300 MHz, DMSO-*d*_6_) δ 13.02 (s, 1H), 10.25 (s, 1H), 8.70 (s, 1H), 8.30 (d, *J* = 1.3 Hz, 1H), 8.20 (dd, *J* = 8.1, 1.6 Hz, 2H), 8.06 (s, 1H), 7.69 (dd, *J* = 9.0, 1.9 Hz, 1H), 7.57–7.46 (m, 4H). ^13^C NMR (75 MHz, DMSO-*d*_6_) δ 159.4, 151.6, 150.3, 137.2, 133.6, 131.3, 130.0, 129.2, 128.5, 127.8, 126.9, 122.7, 121.5, 111.4, 110.0. HPLC-MS [M + H]^+^ = 305, Rt = 3.27 (99%). HRMS (ESI) *m*/*z* calcd. for C_17_H_12_N_4_O_2_Na [M + Na]^+^ 327.0852, found 327.0848.

##### 4-(3,4-Dichlorophenyl)-*N*-(1*H*-indazol-5-yl)-1-methyl-1*H*-pyrrole-3-carboxamide (**102**)

The title compound was prepared by reaction 4-(3,4-dichlorophenyl)-1-methyl-1*H*-pyrrole-3-carboxylic acid (**101**) (200 mg, 0.74 mmol), 1*H*-indazol-5-amine (108 mg, 0.81 mmol), BOP (425 mg, 0.96 mmol) and DIPEA (193 μL, 1.11 mmol) according to the procedure I. Compound **102** was obtained as a white solid after chromatography column. Yield: 84 mg (30%). M.p.: 249–251 °C. ^1^H NMR (300 MHz, DMSO-*d*_6_) δ 12.94 (s, 1H), 9.80 (s, 1H), 8.14 (s, 1H), 8.01 (s, 1H), 7.75 (d, *J* = 2.0 Hz, 1H), 7.57–7.42 (m, 5H), 7.16 (d, *J* = 2.3 Hz, 1H), 3.70 (s, 3H). ^13^C NMR (75 MHz, DMSO-*d*_6_) δ 163.2, 136.8, 136.0, 133.3, 132.6, 130.3, 129.9, 129.4, 128.1, 127.8, 125.9, 123.0, 122.7, 121.6, 121.0, 117.4, 110.1, 109.9, 36.1. HPLC-MS [M + H]^+^ = 385, Rt = 3.43 (95%). HRMS (ESI) *m*/*z* calcd. for C_19_H_14_Cl_2_N_4_ONa [M + Na]^+^ 407.0437, found 407.0432.

### Biology

#### *In Vitro* Inhibition of Human Recombinant Kinases

Commercial SGK1 inhibitors (**EMD638683** and **SGK1-IN4**) were obtained from MedChemExpress and Merck (**GSK650394**). The inhibition of the compounds was evaluated using the Kinase-Glo luminescence assay.^30,41^ The luciferin-luciferase system required for the assay, as well as the recombinant human kinases SGK1 (V2911) and GSK3β (V1991) and the corresponding substrates, were obtained from Promega (Promega Biotech Ibérica). ATP was purchased from ThermoFisher Scientific. The buffer solution used contained 40 mM Tris (pH 7.5), 20 mM MgCl_2_, 0.1 mg·mL^–1^ BSA, and 50 μM DTT. The inhibition assays were performed in 96-well plates in a total volume of 40 μL. To calculate the activity of the compounds, they were evaluated at a concentration of 10 μM starting from a 10 mM solution of the compound in DMSO. The necessary dilutions were made so that the final percentage of DMSO did not exceed 1%. The amount of enzyme used per well was 50 ng and 25 ng for SGK1 and GSK3β, respectively. The substrate was used at a final concentration of 25 μM. The ATP concentration used was 1 μM. The reaction was incubated for 1 h (SGK1) or 30 min (GSK3β) at 30 °C, ending the reaction with the addition of 40 μL of Kinase-Glo reagent. After incubating this reaction for 10 min, the luminescence signal generated was measured using the GloMax Discover Microplate Reader (Promega Biotech Iberica). Maximum enzyme activity (in the absence of the inhibitor) was calculated as the difference between total ATP and ATP consumed. The inhibition of the compounds was calculated based on this maximum activity. The IC_50_ was defined as the concentration of each compound that reduces enzyme activity by 50% relative to maximum activity. IC_50_ values are expressed as the mean of two duplicate experiments ± the standard deviation of the mean.

For selectivity study, the kinase profiling studies were carried out by the MRC Phosphorylation Unit (University of Dundee) using the appropriate protocol in any case.

#### Parallel Artificial Membrane Permeability Assay (PAMPA)

Commercial drugs with known BBB permeability were used as positive (atenolol, ofloxacin, enoxacin, caffeine, piroxicam, and hydrocortisone) and negative (promazine, desipramine, testosterone, and verapamil) controls to validate the experiment and obtain a linear regression model.^32,53^ Controls and SGK1 inhibitors were dissolved in 5 mL of buffer (70:30, PBS pH 7.4:EtOH). 180 μL of each condition were added to the 96-well donor plate (Millipore, catalog no. MAIPS4510) after being covered with 4 μL of porcine brain lipid dissolved in dodecane (20 mg·mL^–1^, Avanti Polar Lipids, catalog no. 141101). The acceptor 96-well plate (Millipore, catalog no. 141101) was filled with 180 μL of the experimental buffer. Then, the donor plate was carefully put on the acceptor plate to form a “sandwich” for 2.5 h at rt. After the incubation time, the passive diffusion of the compounds was determined by UV (Thermoscientific, Multiskan spectrum) in the donor and acceptor plates. Samples were analyzed at 3–5 wavelenghts in three technical replicates in two independent experiments. Results are given as the mean ± standard deviation of the mean of the two independent experiments.

#### Cell Viability

SH-SY5Y cells were incubated in DMEM (Gibco, ThermoFisher Scientific) supplemented with 10% FBS and 100 μg·mL^–1^ penicillin/streptomycin at 37 °C and 5% CO_2_ atmosphere. The cells were seeded in 96-well plates at a concentration of 2·10^5^ cells·mL^–1^. The following day, the cells were incubated with the compounds to be evaluated at the indicated concentration for 1 h. In case of neuroprotection evaluation, OA was then added at a final concentration of 30 nM and incubated for 24 h. Subsequently, MTT was added at a final concentration of 0.5 mg·mL^–1^ and the cells were incubated at 37 °C and 5% CO_2_ for 3 h. Finally, the medium was carefully aspirated, and the formazan crystals were dissolved in 150 μL of DMSO. The UV absorbance was quantified at 560 nM (GloMax Discover Microplate Reader, Promega Biotech Ibérica).

#### Quantification of TAU Phosphorylation

SH-SY5Y cells were incubated as indicated and seeded at a concentration of 5·10^5^ cells·mL^–1^ in 6-well plates. Total protein extracts were obtained by lysing the cells and collecting them by centrifugation, as previously described.^54^ Protein quantification was carried out using the Pierce BCA protein assay kit (ThermoFisher, Madrid Spain).

P-Ser396 TAU (15 μg of total protein extract) and total TAU (5 μg total protein extract) were quantified using two ELISA kits (ThermoFisher Scientific, KHB7031, KHB0041) following the manufacturer’s instructions. Briefly, 50 μL of sample were diluted in 50 μL of dilution buffer and added to a 96-well plate coated with the specific antibody. After incubation and washing, the antibody that recognizes the corresponding epitope was added and incubated. After washing, the horseradish peroxidase (HRP)-conjugated antibody was added. After incubation and washing, the chromogen was added. After 30 min, the reaction was stopped with the stop solution and the absorbance was quantified at 450 nm (GloMax Discover Microplate Reader, Promega Biotech Ibérica, SL). Incubation and washing times, as well as reagent quantities, can be found in the supplier’s protocols.

P-Ser214 TAU was quantified by immunoblotting analysis. Thirty μg of protein were resolved by SDS–polyacrylamide gel electrophoresis (4–15% gradient, Bio-Rad). The transfer was performed on a PDVF membrane (Trans-Blot Turbo Transfer System, Bio-Rad) and blocked with 5% BSA in TBS-T (50 mM Tris-HCl (pH 7.4), 150 mM NaCl, and 0.1% Tween-20) for 1 h. Subsequently, the membrane was incubated with the p-Ser214 TAU antibody (ab170892, Abcam, 1:1000 dilution) overnight at 4 °C. The following day, the membrane was incubated using species-specific antisera antibodies conjugated with HRP (1706515, Bio-Rad, 1:5000 dilution) and detected with a chemiluminescent substrate detection system ECL (Bio-Rad, Alcobendas, Madrid, Spain). Relative band intensities were quantified using a ChemiDoc station with Quantity One 1D analysis software (Bio-Rad Laboratories, Madrid, Spain) and normalized using the intensities of GAPDH (1706515, Bio-Rad, 1:5000 dilution).

#### Pharmacokinetic Studies

The study was conducted according to the guidelines of the Institutional Animal Ethics Committee (IAEC) and approved by Sai Life Sciences (Hinjewadi, Pune, India) (nos. SAIDMPK/PK-23–05–0640 and 13–05–0691) (June 2023), SAIDMPK/PK-23–11–1461 (November 2023), SAIDMPK/PK-24–07–0837 (July 2024). For each compound and administration, 24 male BALB/c mice were used (three for each sampling time point: 0.083, 0.25, 0.5, 1, 2, 4, 8, and 24 h). The mice were 8–12 weeks old and weighed between 19 and 23 g. For the i.p. and i.v. routes, the vehicle was 5% DMSO, 5% Solutol HS-15, and 90% saline. For the p.o. route, 0.5% Tween 80 and 99.5% sodium carboxymethylcellulose (0.5% w/v in water) were used. All formulations were within the analytical acceptability range. All animals were observed to be normal, with no clinical signs during the study period. For each sample, the presence of the compound under study was quantified by LC-MS.

#### Bidirectional Permeability Assay

The study was conducted by Sai Life Sciences. MDR1-transfected MDCKII cells (obtained from The Netherlands Cancer Institute, NKI) were seeded at a density of 1.2 × 10^5^ cells/well onto polyester (PET) Transwell inserts (1.0 μm pore size, Millipore #PSRP010R5) and cultured for 8 days in DMEM supplemented with 10% fetal bovine serum (FBS) and antibiotics. On day 8, cells were preincubated for 30 min at 37 °C, 5% CO_2_ in HBSS buffer (10 mM HEPES, pH 7.4), with or without zosuquidar (5 μM). Transepithelial electrical resistance (TEER) was measured, and only monolayers with TEER > 83 Ω·cm^2^ were used. Bidirectional transport was evaluated in duplicate by adding test compounds (10 μM, 0.1% DMSO) to either the apical or basolateral compartment, while the opposite side contained HBSS buffer (±zosuquidar). Final volumes were 400 μL (apical) and 800 μL (basolateral). Plates were incubated for 120 min at 37 °C, 5% CO_2_. At the end of incubation, samples were collected from both compartments into 96-well deep-well plates. Monolayer integrity was assessed by Lucifer Yellow (100 μM) permeability, and wells with >2% passage to the basolateral side were excluded. Samples (100 μL) were quenched with 200 μL of acetonitrile containing internal standard, vortexed for 5 min, and centrifuged at 4000 rpm for 10 min. Supernatants (100 μL) were transferred to a fresh plate and analyzed by LC-MS/MS.

#### Preclinical Safety Evaluation

Ames test and ion channel inhibition were conducted by Medina Foundation, details of the methodology can be consulted elsewhere.^14^

The cardiac safety profile was evaluated through functional assays targeting three key ion channels: hERG (K^+^), Nav1.5 (Na^+^), and Cav1.2 (Ca^2+^), using HEK293 cell lines stably expressing each respective channel. All assays were performed using a FLIPR TETRA High-Throughput Screening System (Molecular Devices), and IC_50_ values were calculated from 10-point, 1:2 serial dilutions (maximum concentration: 100 μM, in 1% DMSO), tested in triplicate. hERG inhibition was assessed using the FluxOR Potassium Ion Channel Assay (Invitrogen). Cells were loaded with 50 μL of dye-containing loading buffer and incubated at rt for 60 min. After removal of the dye and a single wash with assay buffer, cells were exposed to test compounds for 30 min. The assay was initiated by automated injection of stimulation buffer and fluorescence changes were recorded for 120 s. Nav1.5 channel activity was measured using the FLIPR Membrane Potential Assay. After dye loading, cells were incubated with test compounds, and changes in membrane potential were recorded. Cav1.2 inhibition was evaluated in Cav1.2-HEK293 cells loaded with Fluo-4 dye in assay buffer. After a 30 min incubation, cells were washed with depolarization buffer and treated with test compounds for 30 min. The stimulation buffer was injected to initiate fluorescence recording over 90 s. In all cases, IC_50_ is represented as the mean of three independent experiments.

The mutagenic potential of the test compound was evaluated using the Ames microplate format with *Salmonella typhimurium* strains TA98 and TA100. Bacterial cultures were exposed to four concentrations of the compound dissolved in DMSO (50, 25, 12.5, and 6.25 μM), along with appropriate positive and negative controls, for 90 min in a histidine-enriched medium allowing approximately two cell divisions. Following exposure, cultures were diluted into a histidine-deficient, pH-sensitive medium. After 48 h of incubation, revertant colonies were quantified based on the acidification-induced color change of the medium (yellow/turbid wells considered positive; purple, negative). The assay was conducted both in the absence and presence of liver microsomal fraction (S9 mix) to assess metabolic activation.

#### Liver Microsome Stability Assay

Metabolic stability studies were conducted by Medina Foundation. Assays were conducted in a final incubation volume of 400 μL containing the test compound at 1 μM, microsomal protein at 1 mg/mL, and NADPH at a final concentration of 1.3 mM. The test compound **102** was prepared as a 10 mM stock solution in DMSO and diluted to the desired final concentration (1 μM). NADPH was prepared as a 2.66 mM stock solution in 100 mM potassium phosphate buffer. Reactions were initiated by the addition of mouse, human, or minipig liver microsomes to a prewarmed buffer solution containing the test compound and cofactors. Control incubations lacking NADPH were included to assess non–NADPH-dependent metabolism or chemical instability. Verapamil was used as a positive control to monitor microsomal activity across species. Reactions were terminated at 0, 5, 15, 30, 45, and 60 min by the addition of 60 μL of ice-cold acetonitrile, followed by centrifugation at 3500 rpm for 15 min. Samples were monitored for parent compound disappearance by LC-MS. All experiments were performed in triplicate.

### Computational Studies

#### Docking Experiments

Molecular dynamics simulations of the complex SGK1-**H3** obtained from our previous work^14^ were clustered using affinity propagation algorithm (Schrödinger, v.2024–4). Clustering was based on the RMSD matrix calculated from the binding site residues (within 5 Å of the ligand). The centroid of the most populated cluster was selected as the representative structure for subsequent docking experiments. Ligand were prepared using LigPrep,^55^ as described elsewhere.^13^ Briefly, hydrogen atoms were added where necessary, ionization states were predicted using Epik,^56^ and possible tautomers were generated at pH 7.4 ± 2.0. The most probable state, based on the state penalty, was considered the correct one. Geometry optimization was then performed using the OPLS4 force field.^57^ Docking grid was generated using the ligand as the center, leaving the remaining options as default. An explicit water molecule was modeled to ensure a potential hydrogen bond, and its orientation was assessed using OPLS4 minimization. Ligands were docked using Glide^58,59^ using XP precision. No constraints were applied to the ligand’s conformational search within the binding site, and all other settings were left as default.

Ligand strain energy was calculated using MacroModel, with water as the solvent model.^60^ A restrained minimization of the docked conformation was performed using Cartesian restraints, with a half-width of 0.3 Å and a force constant of 120.00 kcal·mol^–1^·Å^–2^. The energy minimum conformation was obtained using the Monte Carlo Multiple Minimum (MCMM) method, applying a flat-bottomed potential with a half-width of 1.0 Å.^61^ Finally, the strain energy was defined as the energy difference between the docked conformation and the corresponding energy minimum. The cutoff value for adjusted strain docking was set at 4.0 kcal/mol, and the scale factor was set at 0.25.

Induced-fit docking (IFD)^62^ calculations were performed using the Glide and Prime^63^ modules from Schrödinger. The crystallographic structure of P-glycoprotein (P-gp) (PDB ID: 6QEX, chain A) was prepared using the Protein Preparation Workflow.^56^ Briefly, hydrogen atoms were added, ionization states were assigned for a pH of 7.4 ± 2.0, and unresolved loops and side chains were modeled using Prime.^64^ Ions and cosolvents were removed, the hydrogen bond network was optimized with PROPKA at pH 7.4, and the system was gently minimized (RMSD of all atoms <0.3 Å) using the OPLS4 force field.^57^ Finally, water molecules were removed. A maximum of 20 poses of the P-gp–**55** complex were generated, applying van der Waals scaling factors of 0.70 for the receptor and 0.50 for the ligand. Subsequently, the conformations of residues within 5 Å of the ligand were optimized. After protein refinement, the ligand was redocked using XP precision, and the pose with the best IFD score was selected as the most representative.

#### Solvation Energy

Single-point energies for the obtained conformation in docking experiments were carried out with Jaguar in the unbound state, using B3LYP-D3 theory and 6–31G** basis set.^65^ Maximum iterations were set at 200 for convergence criteria. Water was used as the solvent with PBF model, and gas-phase was used as input for reference energy. Solvation energy was calculated as the difference between the solution and gas-phases, leaving the remaining settings as default.

## Supplementary Material





## Notes

Dose–response curve for **GSK650394**, **EMD638683**, and **SGK1-IN4**, the reference compounds for Kinase-Glo methodology; binding mode of SGK1 inhibitors; solvation analysis of derivatives **H3**, **64**, and **65**; docking analysis of the synthesized molecules; *in silico* metabolic study of compound **102**; *P*_*e*_ values from the PAMPA assay for the SGK1 inhibitors; results and descriptors obtained from the Pgp rules prediction; pharmacokinetic profile of compound **83**; kinase panel profiling for compound **102**; chemical procedures to obtain compounds **1**, **14**-**26**, **98** following the general procedure A; chemical procedures to obtain compounds **7**-**10** following the general procedure B; chemical procedures to obtain compounds **2**, **27**-**39**, **99** following the general procedure C; chemical procedures to obtain compounds **81** and **100** following the general procedure D; chemical procedures to obtain compounds **3**, **40**-**52**, **82** and **101** following the general procedure E; chemical procedures to obtain compounds **86**-**90** following the general procedure F; ^1^H and ^13^C NMR spectra and HPLC chromatograms of compounds **53**, **55**, **83**, and **102** (PDF)
Molecular formula strings (CSV)

## Abbreviations Used

AD: Alzheimer’s disease
ATP: adenosine triphosphate
ALS: amyotrophic lateral sclerosis
BBB: blood-brain-barrier
BOP: benzotriazole-1-yl-oxy-tris­(dimethylamino)­phosphonium hexafluorophosphate
BSA: bovine serum albumin
CNS: central nervous system
DCM: dichloromethane
DIPEA: *N*,*N*-diisopropylethylamine
DMEM: Dulbecco’s modified Eagle medium
DMSO: dimethyl sulfoxide
ECBL: European Chemical Biology Library
EDC: 1-ethyl-3-(3-dimethylaminopropyl)­carbodiimide
EtOAc: ethyl acetate
FBS: fetal bovine serum
FDA: Food and Drug Administration
HBTU: hexafluorophosphate benzotriazole tetramethyl uranium
HOBt: 1-hydroxybenzotriazole
HPLC-MS: high performance liquid chromatography coupled to mass spectrometry
HRMS-ESI: high resolution mass spectra
HRP: horseradish peroxidase
IC_50_: half-maximal inhibitory concentration
MW: microwave-assisted synthesis
NMR: nuclear magnetic resonance
OA: okadaic acid
PAMPA: parallel artificial membrane permeability assay
PD: Parkinson’s disease
Pe: effective permeability
P-gp: P-glycoprotein
PP: phosphatase
r.t.: room temperature
SGK1: serum and glucocorticoid regulated kinase 1
TEA: triethylamine
TFA: trifluoroacetic acid
TMSCl: trimethylsilyl chloride
TosMIC: toluenesulfonylmethyl isocyanide
UV: ultraviolet
