# Supplementary material for: Brain Permeable SGK1 Inhibitors: A Promising Therapeutic Strategy for Neurodegenerative Diseases
Source: J Med Chem. 2026 Mar 16;69(6):6790–815. doi: 10.1021/acs.jmedchem.5c03050 (PMC13036784; doi:10.1021/acs.jmedchem.5c03050)
Supplement: Supplementary file 1 [file jm5c03050_si_001.pdf]

Supporting Information

Brain permeable SGK1 inhibitors, A Promising Therapeutic Strategy for Neurodegenerative Diseases

Enrique Madruga,[1,2] Alfonso Garcia-Rubia,[1] Carlos Sanchez-Nuñez,[1] Loreto Martinez-Gonzalez,[1,2] Ana María Fernandez-Escamilla,[3] Isabel Lastres-Becker,[2,4,5] Carmen Gil,[1,2] Ana Martinez[1,2,*]

[1] Centro de Investigaciones Biológicas “Margarita Salas”-CSIC, Ramiro de Maeztu 9, 28040 Madrid, Spain

[2] Centro de Investigación Biomédica en Red en Enfermedades Neurodegeneartivas (CIBERNED), Instituto de Salud Carlos III, 28029 Madrid, Spain

[3] Instituto de Investigación, Desarrollo e Innovación en Biotecnología Sanitaria de Elche (IDiBE), Universitas Miguel Hernández, 03202 Elche, Alicante, Spain.

[4] Instituto de Investigaciones Biomédica " Sols-Morreale”-CSIC/UAM, Arturo Duperier 4, 28039 Madrid, Spain

[5] Instituto de Investigación Sanitaria La Paz (IdiPaz), Paseo de la Castellana, 261 28046 Madrid, Spain.

Correspondence:
Prof. Ana Martinez
e-mail: ana.martinez@csic.es

Index

Figure S1. Dose-response curve for **GSK650394, EMD638683, and SGK1-IN-4** determined following the Kinase-Glo methodology ……………………………………………S3

Figure S2. Binding mode of SGK1 inhibitors……………………………………………………S4

Figure S3. ***In silico*** **metabolic study of compound 102**……………………………………S5

Table S1. Solvation energy of compounds **H3, 64** and **65**……………………………………S6

Table S2. Docking analysis of SGK1 inhibitors…………………………………………………S7

Table S3. P_e values from the PAMPA assay for the SGK1 inhibitors…………………………S8

Table S4. Results and descriptors obtained from the PgpRules prediction for compounds **55** and **83**……………………………………………………………………………S9

Table S5. Pharmacokinetic profile of compound **83** after single dose administration in male BALB/c mice……………………………………………………………………………S10

Table S6. Kinase panel profiling for compound **102** at 10 μM concentration………… S11

Table S7. Intrinsic clearance and t1/2 in liver microsomes…………………………………S12

Compounds prepared following general procedure A (derivatives 1, 14-26, 98).…S13

Compounds prepared following general procedure B (derivatives 7-10)………………S18

Compounds prepared following the General procedure C (derivatives 2,27-39, 99)S20

Compounds prepared following general procedure D (derivatives 81 and 100)….. S25

Compounds prepared by general procedure E (derivatives 3, 40-52, 82 and 101)..S26

Compounds prepared by general procedure F (derivatives 71-75)………………………S31

Compounds prepared following the general procedure G (derivatives 86-90)………S33

^{1}H and ^{13}C NMR spectra and HPLC chromatogram of compound 53………………………S35

^{1}H and ^{13}C NMR spectra and HPLC chromatogram of compound 55………………………S37

^{1}H and ^{13}C NMR spectra and HPLC chromatogram of compound 83………………………S39

^{1}H and ^{13}C NMR spectra and HPLC chromatogram of compound 102……………………S41

Figure S1. Dose-response curve for GSK650394[15], EMD638683, and SGK1-IN4 determined following the Kinase-Glo methodology.

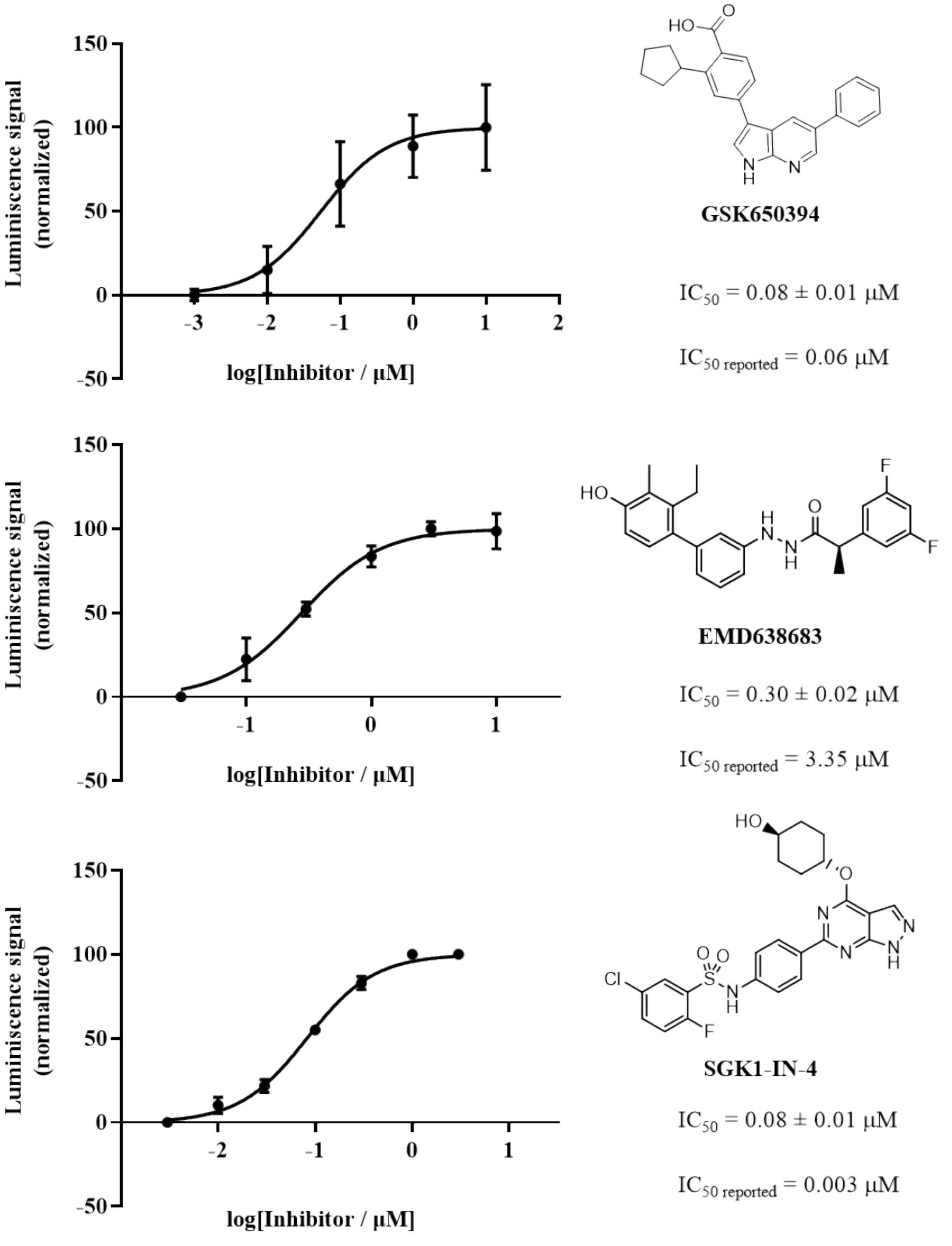

Figure S2. Binding mode of SGK1 (PDB: 3HDM) inhibitors. A) Compounds **59** and **62**, showing steric clashes with the Phe109 (C–H···C distance, 2.1 Å; H + C sum of the van der Waals radius, 2.7 Å). **B)** Compound **11** with the naphthalene moiety, showing a more coplanar and strained conformation. **C)** Pyrazole-derivative compounds **93**-**95**, maintaining the non-coplanar conformation and the interaction pattern. **D)** Compounds **96** and **97**, with isoxazole and oxazole rings, respectively, being not able to interact Glu226.

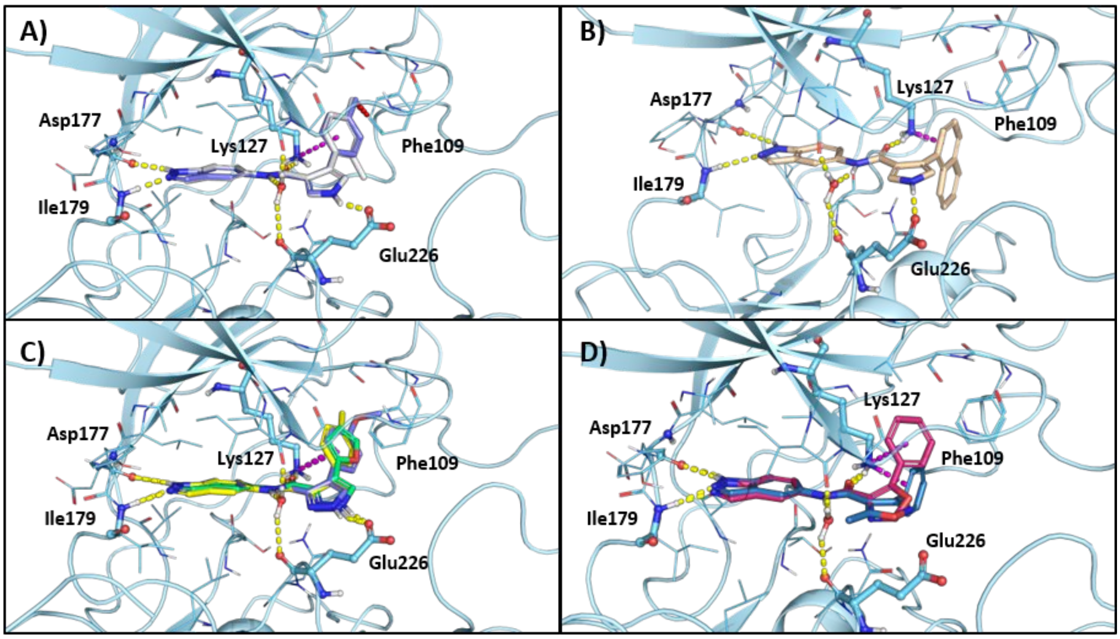

Figure S3. ***In silico*** **metabolic study of compound 102. A)** Molecular metabolic sites predicted by SMARTCyp. The first (red), second (brown), and third (yellow) most probable metabolic sites are shown together with their corresponding scores. **B)** Molecular metabolic sites predicted using the Structure-Based P450 Site of Metabolism module of the Schrödinger Suite. For CYP3A4, the intrinsic reactivity of each metabolic site is shown. For CYP2D6 and CYP2C9, the overall score (linear combination of accessibility and intrinsic reactivity) of the three most probable metabolic sites (from red to yellow) is reported. **C)** Most representative CYP2D6–**102** complex obtained from the induced-fit docking protocol. **D)** Most representative CYP2C9–**102** complex obtained from the induced-fit docking protocol.

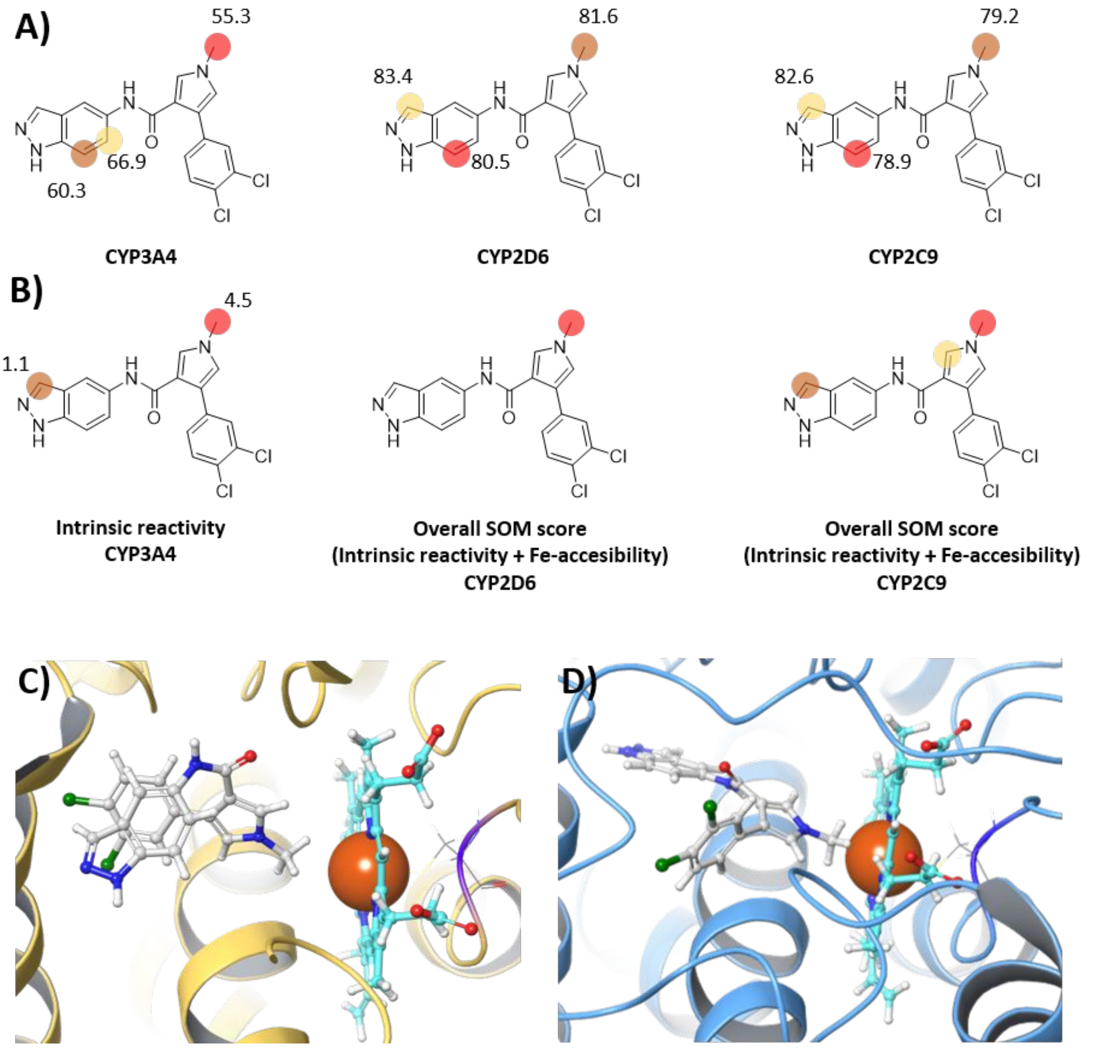

Table S1. Solvation energy of compounds H3, 64 and 65.

Nº	Δ E gas phase [a]	Δ E solution phase [a]	Δ Solvation energy [a]
**H3**	0	0	0
**64**	-10061.837	-10064.887	-3.05
**65**	-10062.584	-10067.369	-4.785

[a] Expressed in kcal·mol^{-1} with respect to **H3.**

Table S2. Docking analysis of SGK1 inhibitors.

nº	IC_{50} (μM) or % inh @ 10 μM	Docking score / $kcal \cdot mol^{-1}$	Strain energy / $kcal \cdot mol^{-1}$	Strain docking score / $kcal \cdot mol^{-1}$
**H3**	0.63 ± 0.01	-10.828	1.736	-10.828
**4**	13.7	-7.192	1.655	-7.192
**5**	11.7	-7.734	1.660	-7.734
**6**	5.25 ± 0.25	-9.856	2.789	-9.856
**11**	2.21 ± 0.18	-7.821	3.082	-7.821
**12**	0.42 ± 0.01	-11.192	1.439	-11.192
**13**[a]	29.2	-	-	-
**53**	0.33 ± 0.07	-10.938	1.554	-10.938
**54**	0.89 ± 0.13	-11.548	1.866	-11.548
**55**	0.11 ± 0.02	-10.923	1.420	-10.923
**56**	0.45 ± 0.11	-11.167	1.981	-11.167
**57**	0.62 ± 0.04	-10.264	1.436	-10.264
**58**	0.57 ± 0.12	-10.557	1.479	-10.557
**59**	1.39 ± 0.34	-11.594	1.872	-11.594
**60**	2.99 ± 0.01	-9.967	2.444	-9.967
**61**[a]	6.68 ± 1.30	-	-	-
**62**	1.22 ± 0.45	-11.240	0.126	-11.240
**63**	0.22 ± 0.02	-11.225	0.803	-11.225
**64**	0.85 ± 0.05	-10.693	0.125	-10.693
**65**	5.87 ± 0.26	-10.365	0.098	-10.365
**66**	8.98 ± 1.59	-10.620	9.889	-9.148
**67**	8.23 ± 0.42	-10.822	11.112	-9.044
**68**	1.67 ± 0.14	-11.137	2.011	-11.137
**69**	1.30 ± 0.20	-8.757	2.320	-8.757
**70**	2.59 ± 0.01	-8.904	6.966	-8.162
**76**	1.55 ± 0.09	-10.072	1.447	-10.072
**77**	8.79 ± 0.85	-8.847	1.147	-8.847
**78**	44.1	-	-	-
**79**	1.73 ± 0.85	-9.978	2.481	-9.978
**80**[a]	1.77 ± 0.09	-	-	-
**83**[a]	2.85 ± 0.37	-	-	-
**85**[a]	2.10 ± 0.67	-	-	-
**91**	0.40 ± 0.02	-10.273	1.649	-10.273
**92**	0.35 ± 0.04	-10.658	1.413	-10.658
**93**	3.85 ± 0.12	-9.894	1.866	-9.894
**94**	6.64 ± 0.07	-9.901	4.198	-9.852
**95**	2.76 ± 0.29	-9.421	0.951	-9.421
**96**[b]	4.87 ± 0.01	-8.561	2.940	-8.561
**97**	6.51 ± 0.54	-10.255	0.307	-10.255

[a] For these compounds, the pose is completely lost. [b] Explicit water molecule needed to be removed to obtain a consistent pose.

Table S3. P_e values from the PAMPA assay for the SGK1 inhibitors.

Nº	P_e / 10^{-6} cm·s^{-1} [a]	CNS categorization	Nº	P_e / 10^{-6} cm·s^{-1}	CNS categorization
**GSK650394**	1.9 ± 0.9	CNS + / CNS -	**64**	0.1 ± 0.1	CNS -
**EMD638683**	1.1 ± 0.7	CNS -	**68**	7.8 ± 0.3	CNS +
**SGK1-IN-4**[b]	-	-	**69**	3.0 ± 0.6	CNS + / CNS -
**12**	9.1 ± 0.4	CNS +	**70**	1.0 ± 0.3	CNS -
**53**	2.4 ± 0.3	CNS + / CNS -	**76**	4.3 ± 0.2	CNS +
**54**	2.5 ± 0.3	CNS + / CNS -	**77**	6.1 ± 0.1	CNS +
**55**	5.1 ± 0.1	CNS +	**79**	0.3 ± 0.1	CNS -
**56**	10.9 ± 0.2	CNS +	**80**	1.1 ± 0.3	CNS -
**57**	7.3 ± 0.1	CNS +	**83**	4.9 ± 0.6	CNS +
**58**	6.3 ± 0.1	CNS +	**85**	4.5 ± 0.1	CNS +
**59**	3.6 ± 0.5	CNS + / CNS -	**91**	2.6 ± 0.2	CNS + / CNS -
**60**	7.6 ± 1.0	CNS +	**92**	4.8 ± 0.8	CNS +
**62**	4.2 ± 0.2	CNS +	**95**	2.5 ± 0.7	CNS + / CNS -
**63**	2.4 ± 0.2	CNS + / CNS -			

[a] Expressed as the mean ± the standard deviation of two independent experiments. [b] Not soluble in assay conditions.

Table S4. Results and descriptors obtained from the Pgp Rules prediction for compounds 55 and 83.

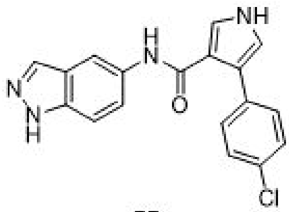

55

P-gp substrate

Descriptor	Result
> = 16 C	V
[#1]-N-C-[#1]	V
> = 1 five-membered rings of carbons, aromatic or saturated	F
> = 32 H	F
C(~C)(~Cl)(~H)	F
Sc1ccc(N)cc1	F
C-C-C-C-C-C-C-C	F
> = 2 five-membered rings	V

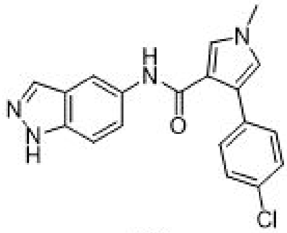

83

No P-gp substrate

Descriptor	Res.
> = 16 C	V
[#1]-N-C-[#1]	F
O=C-C:C-O	F
N#C-C=C	F
> = 5 six-membered rings of carbons, insaturared not aromatic	F
CC1CC(O)CCC1	F
[#1]-C-O-[#1]	F
Cc1ccc(N)cc1	F
C(~C)(~C)(~C)(~O)	F
C-N-C-[#1]	V
O=C-C-C-N	V
C(-N)(=N)	F

Table S5. Pharmacokinetic profile of compound 83 after single dose administration in male BALB/c mice.

Nº	Route	Dose / $mg \cdot kg^{-1}$	Matrix	T_{max} / h	C_{max} / $ng \cdot mL^{-1}$	AUC_{last} / $h \cdot ng \cdot mL^{-1}$	$T_{1/2}$ / h	Brain-Kp (AUC_{last})
**83**	**i.p**	5	Plasma	0.08	1204.18	547.41	0.25	-
			Brain	0.25	199.51	105.94	-	0.19
	**p.o**	10	Plasma	0.25	59.35	53.30	0.97	-
			Brain	0.25	11.08	5.56	-	0.10

Brain C_{max} and AUC_{last} are expressed as $ng \cdot g^{-1}$ and $h \cdot ng \cdot g^{-1}$, respectively. Density of brain tissue was considered as 1 which is equivalent to plasma density.

Table S6. Kinase panel profiling for compound 102 at a fixed concentration of 10 μM.

Target	% inh.	Target	% inh.	Target	% inh.	Target	% inh.	Target	% inh.
**ABL**	1.6	**EPH-A2**	-2.7	**Lck**	45.9	**OSR1**	-3.3	**RSK2**	83.1
**ROCK 2**	98.9	**EPH-A4**	7.0	**LKB1**	-8.6	**p38a MAPK**	39.8	**S6K1**	-1.9
**AMPK**	16.7	**EPH-B1**	22.1	**MAP4K3**	14.7	**p38b MAPK**	24.5	**SGK1**	79.6
**ASK1**	5.4	**EPH-B2**	17.4	**MAP4K5**	6.7	**p38d MAPK**	-5.9	**SIK2**	24.6
**Aurora A**	21.0	**EPH-B3**	2.4	**MAPKAP-K2**	23.1	**p38g MAPK**	5.6	**SIK3**	17.5
**Aurora B**	44.4	**EPH-B4**	12.3	**MAPKAP-K3**	4.6	**PAK2**	27.7	**SmMLCK**	28.0
**BRK**	17.1	**ERK1**	91.2	**MARK1**	12.2	**PAK4**	11.8	**Src**	15.5
**BRSK1**	59.3	**ERK2**	93.3	**MARK2**	2.2	**PAK5**	-8.6	**SRPK1**	2.3
**BRSK2**	59.1	**ERK5**	19.8	**MARK3**	8.5	**PAK6**	8.2	**STK33**	25.4
**BTK**	37.2	**ERK8**	81.8	**MARK4**	3.6	**PDGFRA**	-17.2	**SYK**	15.1
**CAMK1**	47.2	**FGF-R1**	37.4	**MEKK1**	25.2	**PDK1**	20.3	**TAK1**	2.5
**CAMKKb**	13.1	**GCK**	30.0	**MELK**	39.9	**PHK**	-6.6	**TAO1**	17.9
**CDK2-Cyclin A**	24.1	**GSK3b**	64.6	**MINK1**	9.3	**PIM1**	34.7	**TBK1**	7.6
**T1**	-5.4	**HER4**	35.9	**MKK1**	10.7	**PIM2**	-3.5	**TESK1**	12.8
**CHK1**	7.5	**HIPK1**	31.5	**MKK2**	-9.3	**PIM3**	34.1	**TGFBR1**	-26.0
**CHK2**	77.7	**HIPK2**	-4.4	**MKK6**	17.4	**PINK**	20.5	**TIE2**	6.5
**CK1γ2**	6.9	**HIPK3**	50.4	**MLK1**	32.7	**PKA**	75.8	**TLK1**	2.3
**CK1δ**	-12.7	**IGF-1R**	-10.4	**MLK3**	24.0	**PKBa**	14.6	**TrkA**	10.0
**CK2**	2.4	**IKKb**	-3.7	**MNK1**	13.3	**PKBb**	26.0	**TSSK1**	28.7
**CLK2**	94.5	**IKKe**	7.6	**MNK2**	39.0	**PKCa**	21.9	**TTBK1**	16.6
**CSK**	4.4	**IR**	-8.7	**MPSK1**	12.3	**PKCz**	-15.4	**TTBK2**	10.4
**DAPK1**	10.2	**IRAK1**	20.9	**MSK1**	83.3	**PKCγ**	15.2	**TTK**	59.2
**DDR2**	15.5	**IRAK4**	21.8	**MST2**	47.9	**PKD1**	77.1	**ULK1**	-3.1
**DYRK1A**	49.0	**IRR**	40.2	**MST3**	37.5	**PLK1**	28.8	**ULK2**	14.8
**DYRK2**	40.6	**JAK3**	19.2	**MST4**	43.2	**PRAK**	35.9	**VEG-FR**	15.6
**DYRK3**	37.5	**JNK1**	18.8	**NEK2a**	23.6	**PRK2**	35.7	**WNK1**	17.4
**EF2K**	7.3	**JNK2**	-12.8	**NEK6**	26.4	**RIPK2**	42.5	**YES1**	46.7
**EIF 2AK3**	5.2	**JNK3**	12.6	**NUAK1**	62.8	**RSK1**	31.3	**ZAP70**	11.2

Table S7. Intrinsic clearance and t1/2 in liver microsomes. Half-life expressed in min. Intrinsic clearance expressed in mL/min/mg protein. Verapamil was used as standard control.

	**Mouse liver microsomes**		**Human liver microsomes**		**Minipig liver microsomes**	
**Compound**	**Cl_{int} (mL/min/mg protein)**	**$t_{1/2}$ (min)**	**Cl_{int} (mL/min/mg protein)**	**$t_{1/2}$ (min)**	**Cl_{int} (mL/min/mg protein)**	**$t_{1/2}$ (min)**
**102**	311.85	8	38.06	17.56	24.87	20.94
**Verapamil**	1292.64	1.93	226.53	2.95	159.26	3.27

Compounds prepared following general procedure A (compounds 1, 14-26 and 98)

Methyl (*E*)-3-phenylacrylate (1)

The title compound was prepared by reaction of cinnamic acid (1000 mg, 6.75 mmol) and TMSCl (1.9 mL, 14.85 mmol) according to the procedure A. Compound **1** was obtained as a white solid without further purification. Yield: 1012 mg (92%). ^{1}H NMR (300 MHz, DMSO-d_6) δ 7.77 – 7.61 (m, 3H), 7.48 – 7.37 (m, 3H), 6.65 (d, *J* = 16.0 Hz, 1H), 3.73 (s, 3H). ^{13}C NMR (75 MHz, DMSO-d_6) δ 166.7, 144.5, 134.0, 130.5, 128.9, 128.4, 117.8, 51.5. HPLC-MS $[M+H]^+$ = 163, Rt = 3.61 min (99%).

Methyl (*E*)-3-(4-fluorophenyl)acrylate (14)

The title compound was prepared by reaction of (*E*)-3-(4-fluorophenyl)acrylic acid (1520 mg, 9.15 mmol) and TMSCl (2.6 mL, 20.13 mmol) according to the procedure A. Compound **14** was obtained as a white solid without further purification. Yield: 1450 mg (88%). ^{1}H NMR (300 MHz, DMSO-d_6) δ 7.81 (dd, *J* = 8.5, 5.5 Hz, 2H), 7.67 (d, *J* = 16.1 Hz, 1H), 7.26 (t, *J* = 8.8 Hz, 2H), 6.62 (d, *J* = 16.1 Hz, 1H), 3.72 (s, 3H). ^{13}C NMR (75 MHz, DMSO-d_6) δ 166.6, 163.3 (d, J = 250.3 Hz), 143.3, 130.9, 130.7 (d, *J* = 7.1 Hz), 117.7, 115.9 (d, *J* = 21.6 Hz), 51.5. HPLC-MS $[M+H]^+$ = 181, Rt = 3.60 min (99%).

Methyl (*E*)-3-(2-chlorophenyl)acrylate (15)

The title compound was prepared by reaction of (*E*)-3-(2-chlorophenyl)acrylic acid (3000 mg, 16.43 mmol) and TMSCl (4.6 mL, 36.15 mmol) according to the procedure A. Compound **15** was obtained as a white solid without further purification. Yield: 3001 mg (92%). ^{1}H NMR (300 MHz, DMSO-d_6) δ 7.95 (dd, *J* = 7.5, 1.9 Hz, 1H), 7.92 (d, *J* = 16.0 Hz, 1H), 7.55 (dd, *J* = 7.9, 1.5 Hz, 1H), 7.45 (td, *J* = 7.6, 2.0 Hz, 1H), 7.40 (td, *J* = 7.4, 1.4 Hz, 1H), 6.69 (d, *J* = 16.0 Hz, 1H), 3.75 (s, 3H). ^{13}C NMR (75 MHz, DMSO-d_6) δ 166.3, 139.2, 133.7, 131.9, 131.6, 130.0, 128.3, 127.8, 120.9, 51.7. HPLC-MS $[M+H]^+$ = 197, Rt = 3.84 min (99%).

Methyl (*E*)-3-(4-chlorophenyl)acrylate (16)

The title compound was prepared by reaction of (*E*)-3-(4-chlorophenyl)acrylic acid (5000 mg, 27.38 mmol) and TMSCl (7.6 mL, 60.24 mmol) according to the procedure A. Compound **16** was obtained as a white solid without further purification. Yield: 4600

mg (85%). ^{1}H NMR (300 MHz, DMSO-d_6) δ 7.76 (d, *J* = 8.4 Hz, 2H), 7.66 (d, *J* = 16.0 Hz, 1H), 7.48 (d, *J* = 8.4 Hz, 2H), 6.67 (d, *J* = 16.0 Hz, 1H), 3.73 (s, 3H). ^{13}C NMR (75 MHz, DMSO-d_6) δ 166.5, 143.1, 135.0, 133.0, 130.1, 128.9, 118.7, 51.5. HPLC-MS $[M+H]^+$ = 197, Rt = 3.90 min (99%).

Methyl (*E*)-3-(2-bromophenyl)acrylate (17)

The title compound was prepared by reaction of (*E*)-3-(2-bromophenyl)acrylic acid (3000 mg, 13.21 mmol) and TMSCl (3.7 mL, 29.07 mmol) according to the procedure A. Compound **17** was obtained as a yellow oil without further purification. Yield: 2973 mg (93%). ^{1}H NMR (300 MHz, DMSO-d_6) δ 7.94 (dd, *J* = 7.5, 1.8 Hz, 1H), 7.89 (d, *J* = 15.7 Hz, 1H), 7.72 (dd, *J* = 8.0, 1.3 Hz, 1H), 7.44 (tdd, *J* = 7.9, 1.4, 0.6 Hz, 1H), 7.37 (td, *J* = 7.7, 1.9 Hz, 1H), 6.69 (d, *J* = 15.9 Hz, 1H), 3.75 (s, 3H). ^{13}C NMR (75 MHz, DMSO-d_6) δ 166.2, 141.9, 133.3, 133.2, 132.1, 128.5, 128.3, 124.6, 121.0, 51.7. HPLC-MS $[M+H]^+$ = 241, Rt = 3.90 min (95%).

Methyl (*E*)-3-(3-bromophenyl)acrylate (18)

The title compound was prepared by reaction of (*E*)-3-(3-bromophenyl)acrylic acid (3000 mg, 13.21 mmol) and TMSCl (3.7 mL, 29.07 mmol) according to the procedure A. Compound **18** was obtained as a yellow oil without further purification. Yield: 2611 mg (82%). ^{1}H NMR (300 MHz, DMSO-d_6) δ 7.98 (t, *J* = 1.8, 1H), 7.74 (dt, *J* = 7.7, 1.1 Hz, 1H), 7.64 (d, *J* = 16.0 Hz, 1H), 7.61 (ddd, *J* = 8.0, 2.0, 1.0 Hz, 1H), 7.38 (t, *J* = 7.9 Hz, 1H), 6.73 (d, *J* = 16.1 Hz, 1H), 3.73 (s, 3H). ^{13}C NMR (75 MHz, DMSO-d_6) δ 166.4, 142.9, 136.5, 133.0, 130.9, 130.8, 127.3, 122.3, 119.5, 51.6. HPLC-MS $[M+H]^+$ = 241, Rt = 3.90 min (99%).

Methyl (*E*)-3-(4-bromophenyl)acrylate (19)

The title compound was prepared by reaction of (*E*)-3-(4-bromophenyl)acrylic acid (3000 mg, 13.21 mmol) and TMSCl (3.7 mL, 29.07 mmol) according to the procedure A. Compound **19** was obtained as a white solid without further purification. Yield: 2456 mg (77%). ^{1}H NMR (300 MHz, DMSO-d_6) δ 7.69 (d, *J* = 8.5 Hz, 2H), 7.64 (d, *J* = 16.4 Hz, 1H), 7.62 (d, *J* = 8.6 Hz, 2H), 6.69 (d, *J* = 16.1 Hz, 1H), 3.73 (s, 3H). ^{13}C NMR (75 MHz, DMSO-d_6) δ 166.5, 143.2, 133.3, 131.9, 130.3, 123.8, 118.7, 51.5. HPLC-MS $[M+H]^+$ = 241, Rt = 3.92 min (99%).

Methyl (*E*)-3-(*p*-tolyl)acrylate (20)

The title compound was prepared by reaction of (*E*)-3-(*p*-tolyl)acrylic acid (2000 mg, 12.33 mmol) and TMSCl (3.4 mL, 27.13 mmol) according to the procedure A. Compound **20** was obtained as a white solid without further purification. Yield: 2033 mg (94%). ^{1}H NMR (300 MHz, DMSO-d$_{6}$) δ 7.63 (d, *J* = 16.3 Hz, 1H), 7.60 (d, *J* = 8.2 Hz, 2H), 7.23 (d, *J* = 8.0 Hz, 2H), 6.57 (d, *J* = 16.1 Hz, 1H), 3.71 (s, 3H), 2.33 (s, 3H). ^{13}C NMR (75 MHz, DMSO-d$_{6}$) δ 166.8, 144.5, 140.5, 131.3, 129.5, 128.4, 116.7, 51.4, 21.0. HPLC-MS [M+H]$^{+}$ = 177, Rt = 3.81 min (99%).

Methyl (*E*)-3-(4-isopropylphenyl)acrylate (21)

The title compound was prepared by reaction of (*E*)-3-(4-isopropylphenyl)acrylic acid (1000 mg, 5.26 mmol) and TMSCl (1.5 mL, 11.56 mmol) according to the procedure A. Compound **21** was obtained as a white solid without further purification. Yield: 1040 mg (97%). ^{1}H NMR (300 MHz, DMSO-d$_{6}$) δ 7.63 (d, *J* = 16.0 Hz, 1H), 7.63 (d, *J* = 8.3 Hz, 2H), 7.29 (d, *J* = 8.3 Hz, 2H), 6.58 (d, *J* = 16.0 Hz, 1H), 3.72 (s, 3H), 2.91 (hept, *J* = 6.9 Hz, 1H), 1.20 (d, *J* = 6.9 Hz, 6H). ^{13}C NMR (75 MHz, DMSO-d$_{6}$) δ 166.8, 151.2, 144.5, 131.7, 128.5, 126.9, 116.8, 51.4, 33.4, 23.6. HPLC-MS [M+H]$^{+}$ = 205, RT = 4.18 min (99%).

Methyl (*E*)-3-(4-(*tert*-butyl)phenyl)acrylate (22)

The title compound was prepared by reaction of (*E*)-3-(4-(tert-butyl)phenyl)acrylic acid (1000 mg, 4.90 mmol) and TMSCl (1.4 mL, 11.78 mmol) according to the procedure A. Compound **22** was obtained as a white solid without further purification. Yield: 1029 mg (96%). ^{1}H NMR (300 MHz, DMSO-d$_{6}$) δ 7.64 (d, *J* = 8.4 Hz, 2H), 7.64 (d, *J* = 15.9 Hz, 1H), 7.44 (d, *J* = 8.3 Hz, 2H), 6.59 (d, *J* = 16.1 Hz, 1H), 3.72 (s, 3H), 1.28 (s, 9H). ^{13}C NMR (75 MHz, DMSO-d$_{6}$) δ 166.8, 153.4, 144.4, 131.3, 128.2, 125.7, 116.9, 51.4, 34.6, 30.9. HPLC-MS [M+H]$^{+}$ = 219, Rt = 4.23 min (96%).

Methyl (*E*)-3-(2,4-dimethylphenyl)acrylate (23)

The title compound was prepared by reaction of (*E*)-3-(2,4-dimethylphenyl)acrylic acid (1000 mg, 5.67 mmol) and TMSCl (1.6 mL, 12.47 mmol) according to the procedure A. Compound **23** was obtained as a yellow oil without further purification. Yield: 1014 mg (94%). ^{1}H NMR (300 MHz, DMSO-*d*$_{6}$) δ 7.83 (d, *J* = 16.0 Hz, 1H), 7.62 (d, *J* = 7.8 Hz, 1H),

7.08 (s, 1H), 7.05 (d, *J* = 8.1 Hz, 1H), 6.47 (d, *J* = 15.9 Hz, 1H), 3.72 (s, 3H), 2.35 (s, 3H), 2.28 (s, 3H). ^{13}C NMR (75 MHz, DMSO-d_6) δ 167.2, 142.1, 140.6, 137.8, 131.9, 130.3, 127.6, 127.0, 118.1, 51.9, 21.3, 19.7. HPLC-MS [M+H]$^+$ = 191, Rt = 4.00 min (88%).

Methyl (*E*)-3-(4-nitrophenyl)acrylate (24)

The title compound was prepared by reaction of (*E*)-3-(4-nitrophenyl)acrylic acid (1000 mg, 5.18 mmol) and TMSCl (1.4 mL, 11.38 mmol) according to the procedure A. Compound **24** was obtained as a yellow solid without further purification. Yield: 520 mg (49%). ^{1}H NMR (300 MHz, DMSO-d_6) δ 8.24 (d, *J* = 8.9 Hz, 2H), 8.01 (d, *J* = 8.7 Hz, 2H, H-1), 7.78 (d, *J* = 16.1 Hz, 1H), 6.87 (d, *J* = 16.1 Hz, 1H), 3.76 (s, 3H). ^{13}C NMR (75 MHz, DMSO-d_6) δ 166.2, 148.1, 142.0, 140.4, 129.5, 123.9, 122.1, 51.8. HPLC-MS [M+H]$^+$ = 208, Rt = 3.51 min (97%).

Methyl (*E*)-3-(pyridin-3-yl)acrylate (25)

The title compound was prepared by reaction of (*E*)-3-(pyridine-3-yl)acrylic acid (1000 mg, 6.70 mmol) and TMSCl (1.9 mL, 14.74 mmol) according to the procedure A. Compound **25** was obtained as a white solid without further purification. Yield: 1005 mg (92%). ^{1}H NMR (300 MHz, DMSO-d_6) δ 8.88 (d, *J* = 2.3 Hz, 1H), 8.59 (dd, *J* = 4.8, 1.6 Hz, 1H), 8.18 (ddd, *J* = 8.0, 2.2, 1.6 Hz, 1H), 7.70 (dd, *J* = 16.2, 0.6 Hz, 1H), 7.45 (dddd, *J* = 8.0, 4.8, 0.9, 0.4 Hz, 1H), 6.80 (d, *J* = 16.2 Hz, 1H), 3.74 (s, 3H). ^{13}C NMR (75 MHz, DMSO-d_6) δ 166.3, 151.0, 149.9, 141.3, 134.7, 129.8, 123.9, 119.8, 51.6. HPLC-MS [M+H]$^+$ = 164, Rt = 2.21 min (99%).

Methyl (*E*)-3-(pyridin-4-yl)acrylate (26)

The title compound was prepared by reaction of (*E*)-3-(pyridine-4-yl)acrylic acid (1000 mg, 6.70 mmol) and TMSCl (1.9 mL, 14.74 mmol) according to the procedure A. Compound **26** was obtained as a white solid without further purification. Yield: 960 mg (88%). ^{1}H NMR (300 MHz, DMSO-d_6) δ 8.63 (d, *J* = 6.1 Hz, 2H), 7.68 (d, *J* = 6.3 Hz, 2H), 7.64 (d, *J* = 16.1 Hz, 1H), 6.90 (d, *J* = 16.1 Hz, 1H), 3.75 (s, 3H). ^{13}C NMR (75 MHz, DMSO-d_6) δ 166.1, 150.4, 141.9, 141.1, 122.5, 122.2, 51.8. HPLC-MS [M+H]$^+$ = 164, Rt = 1.88 min (99%).

Methyl (*E*)-3-(3,4-dichlorophenyl)acrylate (98)

The title compound was prepared by reaction of (*E*)-3-(3,4-dichlorophenyl)acrylic acid (2000 mg, 9.21 mmol) and TMSCl (2.57 mL, 20.26 mmol) according to the procedure A. Compound **98** was obtained as a white solid without further purification. Yield: 1824 mg (86%). ^{1}H NMR (300 MHz, DMSO-d_6) δ 8.07 (d, J = 2.0 Hz, 1H), 7.74 (dd, J = 8.5, 1.9 Hz, 1H), 7.68 (d, J = 8.4 Hz, 1H), 7.64 (d, J = 16.1 Hz, 1H), 6.77 (d, J = 16.2 Hz, 1H, 3.73 (s, 3H). ^{13}C NMR (75 MHz, DMSO-d_6) δ 166.3, 141.9, 134.9, 132.7, 131.8, 131.0, 130.1, 128.3, 120.1, 51.6. HPLC-MS $[M+H]^+$ = 231, Rt = 4.08 min (99%).

Compounds prepared following general procedure B (derivatives 7-10)

***N*-(1*H*-indazol-5-yl)cinnamamide (7)**

The title compound was prepared by reaction of cinnamic acid (400 mg, 2.70 mmol), DIPEA (1.4 mL, 8.10 mmol), 1*H*-indazol-5-amine (395 mg, 2.97 mmol) and HBTU (1536 mg, 4.05 mmol) according to the procedure B. Compound **7** was obtained as a precipitated white solid after column chromatography (Hexane/AcOEt, 1:1). Yield: 455 mg (64%). ^{1}H NMR (300 MHz, DMSO-d_6) δ 13.00 (s, 1H), 10.22 (s, 1H), 8.29 (s, 1H), 8.05 (d, *J* = 1.3 Hz, 1H), 7.66 – 7.56 (m, 3H), 7.53 – 7.37 (m, 5H), 6.87 (d, *J* = 15.7 Hz, 1H). ^{13}C NMR (75 MHz, DMSO-d_6) δ 163.4, 139.7, 136.9, 134.8, 133.5, 132.3, 129.7, 129.0, 127.7, 122.8, 122.5, 120.2, 110.3, 109.7. HPLC-MS $[M+H]^+$ = 264, Rt = 3.02 min (99%).

(*E*)-*N*-(1*H*-indazol-5-yl)-3-(naphthalen-1-yl)acrylamide (8)

The title compound was prepared by reaction of (*E*)-3-(naphtalen-1-yl)acrylic acid (500 mg, 2.52 mmol), DIPEA (1.3 mL, 7.56 mmol), 1*H*-indazol-5-amine (369 mg, 2.77 mmol) and HBTU (1435 mg, 3.78 mmol) according to the procedure B. Compound **8** was obtained as a precipitated white solid without further purification. Yield: 412 mg (52%). ^{1}H NMR (300 MHz, DMSO-d_6) δ 13.02 (s, 1H), 10.32 (s, 1H), 8.37 (d, *J* = 15.5 Hz, 1H), 8.33 (s, 1H), 8.26 (d, *J* = 8.8 Hz, 1H), 8.07 (s, 1H), 8.05 – 7.96 (m, 2H), 7.86 (d, *J* = 7.2 Hz, 1H), 7.69 – 7.51 (m, 5H), 6.95 (d, *J* = 15.5 Hz, 1H). ^{13}C NMR (75 MHz, DMSO-d_6) δ 163.3, 137.0, 136.2, 133.5, 133.4, 132.3, 132.0, 130.8, 129.8, 128.7, 127.0, 126.3, 125.8, 125.5, 124.7, 123.3, 122.8, 120.3, 110.3, 109.8. HPLC-MS $[M+H]^+$ = 314, Rt = 3.26 min (98%).

***Tert*-butyl (*E*)-5-(3-(3-chlorophenyl)acrylamido)-1*H*-indazole-1-carboxylate (9)**

The title compound was prepared by reaction of (*E*)-3-(3-chlorophenyl)acrylic acid (390 mg, 2.14 mmol), DIPEA (1.1 mL, 6.44 mmol), *tert*-butyl 5-amino-1*H*-indazole-1-carboxylate (549 mg, 2.35 mmol) and HBTU (1219 mg, 3.22 mmol) according to the procedure B. Compound **9** was obtained as a white solid after column chromatography (CH_2Cl_2/MeOH, 9:1). Yield: 532 mg (60%). ^{1}H NMR (300 MHz, DMSO-d_6) δ 10.45 (s, 1H), 8.43 (d, *J* = 0.7 Hz, 1H), 8.40 (d, *J* = 1.5 Hz, 1H), 8.04 (d, *J* = 9.0 Hz, 1H), 7.78 – 7.70 (m, 2H), 7.65 – 7.57 (m, 2H), 7.52 – 7.44 (m, 2H), 6.91 (d, *J* = 15.8 Hz, 1H), 1.65 (s, 9H). ^{13}C NMR (75 MHz, DMSO-d_6) δ 163.3, 148.5, 140.0, 138.6, 137.0, 135.5, 135.2, 133.7,

130.8, 129.4, 127.4, 126.2, 125.9, 123.9, 122.0, 114.3, 110.6, 84.4, 27.7. HPLC-MS [M + H - *tert*]$^+$ = 342, Rt = 3.05 (90%).

***Tert*-butyl (*E*)-5-(3-([1,1'-biphenyl]-4-yl)acrylamido)-1*H*-indazole-1-carboxylate (10)**

The title compound was prepared by reaction of (*E*)- 3-([1,1'-biphenyl]-4-yl)acrylic acid (224 mg, 1.07 mmol), DIPEA (559 µL, 3.21 mmol), *tert*-butyl 5-amino-1*H*-indazole-1-carboxylate (275 mg, 1.18 mmol) and HBTU (610 mg, 1.61 mmol) according to the procedure B. Compound **10** was obtained as a precipitated white solid without further purification. Yield: 250 mg (53%). ^{1}H NMR (300 MHz, DMSO-d_6) δ 10.46 (s, 1H), 8.43 (s, 1H), 8.42 (s, 1H), 8.05 (d, *J* = 8.9 Hz, 1H), 7.82 – 7.62 (m, 8H), 7.49 (t, *J* = 7.4 Hz, 2H), 7.39 (t, *J* = 7.3 Hz, 1H), 6.91 (d, *J* = 15.7 Hz, 1H), 1.65 (s, 9H). ^{13}C NMR (75 MHz, DMSO-d_6) δ 163.7, 148.5, 141.3, 140.0, 139.8, 139.3, 135.5, 135.4, 133.8, 129.0, 128.4, 127.9, 127.2, 126.6, 126.0, 122.1, 122.0, 114.3, 110.5, 84.4, 27.7. HPLC-MS [M + H]$^+$ = 440, Rt = 4.02 (99%).

Compounds prepared following the General procedure C (derivatives 2, 27-39 and 99)

Methyl 4-phenyl-1H-pyrrole-3-carboxylate (2)

The title compound was prepared by reaction of methyl (*E*)-3-phenylacrylate **(1)** (1500 mg, 9.25 mmol), TosMIC (1986 mg, 10.17 mmol), and NaH (1100 mg, 27.75 mmol) according to the procedure C. Compound **2** was obtained as a brown solid without further purification. Yield: 1369 mg (74%). ^{1}H NMR (300 MHz, DMSO-d_6) δ 11.55 (s, 1H), 7.49 (dd, *J* = 3.1, 2.2 Hz, 1H), 7.44 (m, 2H), 7.30 (t, *J* = 7.3 Hz, 2H), 7.24 – 7.18 (m, 1H), 6.94 (t, *J* = 2.4 Hz, 1H), 3.63 (s, 3H). ^{13}C NMR (75 MHz, DMSO-d_6) δ 164.5, 135.0, 128.8, 127.6, 125.9, 125.8, 125.2, 119.1, 111.6, 50.4. HPLC-MS $[M + H]^+$ = 202, Rt = 3.23 (99%).

Methyl 4-(4-fluorophenyl)-1*H*-pyrrole-3-carboxylate (27)

The title compound was prepared by reaction of methyl (*E*)-3-(4-fluorophenyl)acrylate **(14)** (1427 mg, 7.92 mmol), TosMIC (1700 mg, 8.71 mmol), and NaH (950 mg, 27.76 mmol) according to the procedure C. Compound **27** was obtained as a brown solid without further purification. Yield: 841 mg (63%). ^{1}H NMR (300 MHz, DMSO-d_6) δ 11.57 (s, 1H), 7.51 – 7.42 (m, 3H), 7.12 (t, *J* = 9.0 Hz, 2H), 6.94 (t, *J* = 2.4 Hz, 1H), 3.63 (s, 3H). ^{13}C NMR (75 MHz, DMSO-d_6) δ 164.5, 160.9 (d, *J* = 242.0 Hz), 131.4 (d, *J* = 3.1 Hz), 130.6 (d, *J* = 7.9 Hz), 125.9, 124.1, 119.2, 114.3 (d, *J* = 21.1 Hz), 111.5, 50.4. HPLC-MS $[M + H]^+$ = 220, Rt = 3.27 (95%).

Methyl 4-(2-chlorophenyl)-1*H*-pyrrole-3-carboxylate (28)

The title compound was prepared by reaction of methyl (*E*)-3-(2-chlorophenyl)acrylate **(15)** (3000 mg, 15.25 mmol), TosMIC (3276 mg, 16.78 mmol), and NaH (1829 mg, 45.75 mmol) according to the procedure C. Compound **28** was obtained as a brown solid without further purification. Yield: 2500 mg (70%). ^{1}H NMR (300 MHz, DMSO-d_6) δ 11.57 (s, 1H), 7.47 (dd, *J* = 3.1, 2.1 Hz, 1H), 7.45 – 7.40 (m, 1H), 7.31 – 7.27 (m, 3H), 6.85 (t, *J* = 2.4 Hz, 1H), 3.55 (s, 3H). ^{13}C NMR (75 MHz, DMSO-d_6) δ 164.2, 134.7, 133.4, 132.1, 128.8, 128.1, 126.3, 124.4, 121.8, 119.6, 113.5, 50.4. HPLC-MS $[M + H]^+$ = 236, Rt = 3.31 (93%).

Methyl 4-(4-chlorophenyl)-1*H*-pyrrole-3-carboxylate (29)

The title compound was prepared by reaction of methyl (*E*)-3-(4-chlorophenyl)acrylate **(16)** (2000 mg, 10.17 mmol), TosMIC (2184 mg, 11.19 mmol), and NaH (1220 mg, 30.51 mmol) according to the procedure C. Compound **29** was obtained as a pale yellow solid without further purification. Yield: 1830 mg (76%). ^{1}H NMR (300 MHz, DMSO-d_6) δ 11.61 (s, 1H), 7.50 (dd, *J* = 3.1, 2.2 Hz, 1H), 7.46 (d, *J* = 8.7 Hz, 2H), 7.35 (d, *J* = 8.8 Hz, 2H, 6.99 (t, *J* = 2.4 Hz, 1H), 3.64 (s, 3H). ^{13}C NMR (75 MHz, DMSO-d_6) δ 164.4, 133.9, 130.5, 130.5, 127.5, 126.2, 123.8, 119.5, 111.5, 50.5. HPLC-MS [M + H]$^+$ = 236, Rt = 3.49 (99%).

Methyl 4-(2-bromophenyl)-1*H*-pyrrole-3-carboxylate (30)

The title compound was prepared by reaction of methyl (*E*)-3-(2-bromophenyl)acrylate **(17)** (1000 mg, 4.15 mmol), TosMIC (981 mg, 4.56 mmol), and NaH (498 mg, 12.44 mmol) according to the procedure C. Compound **30** was obtained as a brown solid without further purification. Yield: 846 mg (73%). ^{1}H NMR (300 MHz, DMSO-d_6) δ 11.55 (s, 1H), 7.61 (dd, *J* = 7.9, 0.9 Hz, 1H), 7.46 (dd, *J* = 3.1, 2.2 Hz, 1H), 7.36 – 7.25 (m, 2H), 7.20 (ddd, *J* = 8.0, 7.0, 2.3 Hz, 1H), 6.83 (t, *J* = 2.4 Hz, 1H), 3.54 (s, 3H). ^{13}C NMR (75 MHz, DMSO-d_6) δ 164.1, 136.8, 132.1, 131.9, 128.3, 126.8, 124.5, 124.3, 123.7, 119.4, 113.4, 50.3. HPLC-MS [M + H]$^+$ = 280, Rt = 3.34 (99%).

Methyl 4-(3-bromophenyl)-1*H*-pyrrole-3-carboxylate (31)

The title compound was prepared by reaction of methyl (*E*)-3-(3-bromophenyl)acrylate **(18)** (1000 mg, 4.15 mmol), TosMIC (981 mg, 4.56 mmol), and NaH (498 mg, 12.44 mmol) according to the procedure C. Compound **31** was obtained as a brown solid without further purification. Yield: 836 mg (72%). ^{1}H NMR (300 MHz, DMSO-d_6) δ 11.66 (s, 1H), 7.67 (t, *J* = 1.8 Hz, 1H), 7.51 (t, *J* = 2.6 Hz, 1H), 7.45 (dt, *J* = 7.6, 1.3 Hz, 1H), 7.41 (ddd, *J* = 8.0, 2.0, 1.1 Hz, 1H), 7.27 (t, *J* = 7.9 Hz, 1H), 7.05 (t, *J* = 2.3 Hz, 1H), 3.65 (s, 3H). ^{13}C NMR (75 MHz, DMSO-d_6) δ 164.4, 137.5, 131.2, 129.7, 128.5, 127.7, 126.3, 123.5, 120.9, 119.9, 111.5, 50.5. HPLC-MS [M + H]$^+$ = 280, Rt = 3.53 (98%).

Methyl 4-(4-bromophenyl)-1*H*-pyrrole-3-carboxylate (32)

The title compound was prepared by reaction of methyl (*E*)-3-(4-bromophenyl)acrylate **(19)** (1000 mg, 4.15 mmol), TosMIC (981 mg, 4.56 mmol), and NaH (498 mg, 12.44 mmol) according to the procedure C. Compound **32** was obtained as a brown solid without further purification. Yield: 783 mg (67%). ^{1} NMR (300 MHz, DMSO-d_6) δ 11.64 (s, 1H), 7.52 – 7.46 (m, 3H), 7.40 (d, *J* = 8.7 Hz, 2H), 7.00 (t, *J* = 2.0 Hz, 1H), 3.64 (s, 3H). ^{13}C NMR (75 MHz, DMSO-d_6) δ 164.4, 134.3, 130.8, 130.4, 126.2, 123.8, 119.5, 119.0, 111.5, 50.5. HPLC-MS $[M + H]^+$ = 280, Rt = 3.55 (97%).

Methyl 4-(p-tolyl)-1*H*-pyrrole-3-carboxylate (33)

The title compound was prepared by reaction of methyl (*E*)-3-(*p*-tolyl)phenyl)acrylate **(20)** (2000 mg, 11.35 mmol), TosMIC (2437 mg, 12.48 mmol), and NaH (1361 mg, 34.05 mmol) according to the procedure C. Compound **33** was obtained as a brown solid without further purification. Yield: 2156 mg (88%). ^{1}H NMR (300 MHz, DMSO-d_6) δ 11.50 (s, 1H), 7.46 (dd, *J* = 3.1, 2.2 Hz, 1H), 7.32 (d, *J* = 8.0 Hz, 2H), 7.10 (d, *J* = 7.9 Hz, 2H), 6.89 (t, *J* = 2.4 Hz, 1H), 3.62 (s, 3H), 2.30 (s, 3H). ^{13}C NMR (75 MHz, DMSO-d_6) δ 164.5, 134.8, 132.1, 128.7, 128.2, 125.8, 125.1, 118.8, 111.6, 50.4, 20.7. HPLC-MS $[M + H]^+$ = 216, Rt = 3.39 (90%).

Methyl 4-(4-isopropylphenyl)-1*H*-pyrrole-3-carboxylate (34)

The title compound was prepared by reaction of methyl (*E*)-3-(4-isopropylphenyl)acrylate **(21)** (1040 mg, 5.09 mmol), TosMIC (1093 mg, 5.60 mmol), and NaH (610 mg, 15.27 mmol) according to the procedure C. Compound **34** was obtained as a brown solid without further purification. Yield: 910 mg (74%). ^{1}H NMR (300 MHz, DMSO-d_6) δ 11.51 (s, 1H), 7.46 (dd, *J* = 3.1, 2.2 Hz, 1H), 7.35 (d, *J* = 8.3 Hz, 2H), 7.17 (d, *J* = 8.1 Hz, 2H), 6.90 (t, *J* = 2.4 Hz, 1H), 3.63 (s, 3H), 2.88 (hept, *J* = 6.9 Hz, 1H), 1.22 (d, *J* = 7.0 Hz, 6H). ^{13}C NMR (75 MHz, DMSO-d_6) δ 164.5, 145.9, 132.5, 128.7, 125.7, 125.5, 125.2, 118.9, 111.5, 50.4, 33.1, 24.0. HPLC-MS $[M + H]^+$ = 244, Rt = 3.74 (99%).

Methyl 4-(4-(*tert*-butyl)phenyl)-1*H*-pyrrole-3-carboxylate (35)

The title compound was prepared by reaction of methyl (*E*)-3-(4-(*tert*-butyl)phenyl)acrylate **(22)** (1000 mg, 4.58 mmol), TosMIC (984 mg, 5.03 mmol), and NaH (549 mg, 13.74 mmol) according to the procedure C. Compound **35** was obtained

as a brown solid without further purification. Yield: 1040 mg (80%). ^{1}H NMR (300 MHz, DMSO-d_6) δ 11.53 (s, 1H), 7.47 (dd, *J* = 3.0, 2.2 Hz, 1H), 7.37 (d, *J* = 8.6 Hz, 2H), 7.31 (d, *J* = 8.6 Hz, 2H), 6.90 (t, *J* = 2.3 Hz, 1H), 3.64 (s, 3H), 1.30 (s, 9H). ^{13}C NMR (75 MHz, DMSO-d_6) δ 164.5, 148.1, 132.1, 128.5, 125.8, 125.1, 124.3, 118.9, 111.5, 50.4, 34.1, 31.2. HPLC-MS $[M + H]^+$ = 258, Rt = 3.85 (98%).

Methyl 4-(2,4-dimethylphenyl)-1*H*-pyrrole-3-carboxylate (36)

The title compound was prepared by reaction of methyl (*E*)-3-(2,4-dimethylphenyl)acrylate **(23)** (1000 mg, 5.26 mmol), TosMIC (1127 mg, 5.78 mmol), and NaH (630 mg, 15.78 mmol) according to the procedure C. Compound **36** was obtained as a brown solid without further purification. Yield: 810 mg (67%). ^{1}H NMR (300 MHz, DMSO-d_6) δ 11.50 (s, 1H), 7.47 (dd, *J* = 3.1, 2.2 Hz, 1H), 7.02 – 6.94 (m, 2H), 6.91 (d, *J* = 7.7 Hz, 1H), 6.69 (t, *J* = 2.3 Hz, 1H), 3.55 (s, 3H), 2.27 (s, 3H), 2.07 (s, 3H). ^{13}C NMR (75 MHz, DMSO-d_6) δ 164.4, 136.4, 135.3, 132.5, 130.3, 129.9, 125.4, 124.6, 124.2, 118.8, 113.2, 50.2, 20.7, 20.0. HPLC-MS $[M + H]^+$ = 230, Rt = 3.53 (83%).

Methyl 4-(4-nitrophenyl)-1*H*-pyrrole-3-carboxylate (37)

The title compound was prepared by reaction of methyl (*E*)-3-(4-nitrophenyl)acrylate **(24)** (520 mg, 2.51 mmol), TosMIC (539 mg, 2.76 mmol), and NaH (301 mg, 7.53 mmol) according to the procedure C. Compound **37** was obtained as a brown solid without further purification. Yield: 502 mg (81%). ^{1}H NMR (300 MHz, DMSO-d_6) δ 11.82 (s, 1H), 8.17 (d, *J* = 9.0 Hz, 2H), 7.75 (d, *J* = 9.0 Hz, 2H), 7.58 (dd, *J* = 3.0, 2.2 Hz, 1H), 7.22 (t, *J* = 2.4 Hz, 1H), 3.67 (s, 3H). ^{13}C NMR (75 MHz, DMSO-d_6) δ 164.4, 145.3, 142.3, 129.4, 127.0, 123.0, 122.9, 121.1, 111.8, 50.7. HPLC-MS $[M + H]^+$ = 247, Rt = 3.26 (94%).

Methyl 4-(pyridin-3-yl)-1*H*-pyrrole-3-carboxylate (38)

The title compound was prepared by reaction of methyl (*E*)-3-(pyridine-3-yl)acrylate **(25)** (500 mg, 3.06 mmol), TosMIC (658 mg, 3.37 mmol), and NaH (367 mg, 9.18 mmol) according to the procedure C. Compound **38** was obtained as a yellow solid without further purification. Yield: 440 mg (71%). ^{1}H NMR (300 MHz, DMSO-d_6) δ 11.70 (s, 1H), 8.63 (dd, *J* = 2.3, 0.8 Hz, 1H), 8.41 (dd, *J* = 4.8, 1.7 Hz, 1H), 7.84 (ddd, *J* = 7.9, 2.2, 1.7 Hz, 1H), 7.55 (dd, *J* = 3.1, 2.2 Hz, 1H), 7.33 (ddd, *J* = 7.9, 4.8, 0.8 Hz, 1H), 7.08 (t, *J* = 2.4 Hz,

1H), 3.65 (s, 3H). ^{13}C NMR (75 MHz, DMSO-d_6) δ 164.4, 149.2, 146.9, 136.1, 130.8, 126.3, 122.7, 121.5, 119.8, 111.7, 50.5. HPLC-MS $[M + H]^+$ = 203, Rt = 1.86 (95%).

Methyl 4-(pyridin-4-yl)-1*H*-pyrrole-3-carboxylate (39)

The title compound was prepared by reaction of methyl (*E*)-3-(pyridine-4-yl)acrylate **(26)** (500 mg, 3.06 mmol), TosMIC (658 mg, 3.37 mmol), and NaH (367 mg, 9.18 mmol) according to the procedure C. Compound **39** was obtained as a brown solid without further purification. Yield: 367 mg (62%). ^{1}H NMR (300 MHz, DMSO-d_6) δ 11.78 (s, 1H), 8.47 (d, *J* = 6.1 Hz, 2H), 7.56 (dd, *J* = 3.1, 2.2 Hz, 1H), 7.49 (d, *J* = 6.1 Hz, 2H), 7.21 (dd, *J* = 2.7, 2.2 Hz, 1H), 3.67 (s, 3H). ^{13}C NMR (75 MHz, DMSO-d_6) δ 164.4, 148.9, 142.5, 126.9, 123.3, 122.3, 120.8, 111.8, 50.7. HPLC-MS $[M + H]^+$ = 203, Rt = 1.85 (95%).

Methyl 4-(3,4-dichlorophenyl)-1*H*-pyrrole-3-carboxylate (99)

The title compound was prepared by reaction of methyl (*E*)-3-(3,4-dichlorophenyl)acrylate **(98)** (1500 mg, 6.49 mmol), TosMIC (1395 mg, 7.15 mmol), and NaH (778 mg, 19.47 mmol) according to the procedure C. Compound **99** was obtained as a brown solid without further purification. Yield: 994 mg (57%). ^{1}H NMR (300 MHz, DMSO-d_6) δ 11.73 (s, 1H), 7.74 (d, *J* = 2.1 Hz, 1H), 7.55 (d, *J* = 8.4 Hz, 1H), 7.52 (dd, *J* = 3.0, 2.3 Hz, 1H), 7.45 (dd, *J* = 8.4, 2.1 Hz, 1H), 7.11 (t, *J* = 2.4 Hz, 1H), 3.66 (s, 3H). ^{13}C NMR (75 MHz, DMSO-d_6) δ 164.4, 135.8, 130.2, 130.2, 129.6, 128.9, 128.3, 126.5, 122.5, 120.2, 111.5, 50.6. HPLC-MS $[M + H]^+$ = 270, Rt = 3.72 (99 %).

Compounds prepared following general procedure D (derivatives 81 and 100)

Methyl 4-(4-chlorophenyl)-1-methyl-1*H*-pyrrole-3-carboxylate (81)

The title compound was prepared by reaction of methyl 4-(4-chlorophenyl)acrylate **(29)** (400 mg, 1.70 mmol), NaH (88 mg, 2.21 mmol) and CH_3I (212 µL, 3.40 mmol) according to the procedure D. Compound **81** was obtained as a white solid after chromatography column. Yield: 242 mg (57%). ^1H NMR (300 MHz, DMSO-d_6) δ 7.50 (d, *J* = 2.4 Hz, 1H), 7.44 (d, *J* = 8.7 Hz, 2H), 7.35 (d, *J* = 8.7 Hz, 2H), 6.97 (d, *J* = 2.4 Hz, 1H), 3.67 (s, 3H), 3.63 (s, 3H). ^{13}C NMR (75 MHz, DMSO-d_6) δ 164.0, 133.5, 130.6, 130.3, 129.6, 127.6, 124.2, 123.3, 111.2, 50.5, 36.1. HPLC-MS $[M + H]^+$ = 250, Rt = 3.78 (99%).

Methyl 4-(3,4-dichlorophenyl)-1-methyl-1*H*-pyrrole-3-carboxylate (100)

The title compound was prepared by reaction of methyl 4-(3,4-dichlorophenyl)acrylate **(99)** (500 mg, 1.76 mmol), NaH (91 mg, 2.29 mmol) and CH_3I (219 µL, 3.52 mmol) according to the procedure D. Compound **100** was obtained as a white solid after chromatography column. Yield: 332 mg (66%). ^1H NMR (300 MHz, DMSO-d_6) δ 7.71 (d, *J* = 2.0 Hz, 1H), 7.56 (d, *J* = 8.4 Hz, 1H), 7.53 (d, *J* = 2.4 Hz, 1H), 7.42 (dd, *J* = 8.4, 2.1 Hz, 1H), 7.09 (d, *J* = 2.4 Hz, 1H), 3.67 (s, 3H), 3.65 (s, 3H). ^{13}C NMR (75 MHz, DMSO-d_6) δ 164.0, 135.4, 130.2, 130.2, 129.9, 129.7, 128.8, 128.4, 123.9, 122.8, 111.2, 50.6, 36.2. HPLC-MS $[M + H]^+$ = 284, Rt = 4.01 (95%).

Compounds prepared by general procedure E (derivatives 3, 40-52, 70 and 89)

4-Phenyl-1*H*-pyrrole-3-carboxylic acid (3)

The title compound was prepared by reaction of methyl 4-phenyl-1*H*-pyrrole-3-carboxylate **(2)** (1000 mg, 4.97 mmol), and NaOH (1988 mg, 49.70 mmol) according to the procedure E. Compound **3** was obtained as a white solid after precipitation. Yield: 640 mg (69%). ^{1}H NMR (300 MHz, DMSO-d_6) δ 11.54 (s, 1H), 11.45 (s, 1H), 7.51 – 7.41 (m, 3H), 7.29 (t, *J* = 7.4 Hz, 2H), 7.23 – 7.16 (m, 1H), 6.91 (t, *J* = 2.3 Hz, 1H). ^{13}C NMR (75 MHz, DMSO-d_6) δ 165.7, 135.3, 128.8, 127.5, 126.0, 125.7, 125.2, 119.0, 112.6. HPLC-MS $[M + H]^+$ = 188, Rt = 2.74 (99%).

4-(4-Fluorophenyl)-1*H*-pyrrole-3-carboxylic acid (40)

The title compound was prepared by reaction of methyl 4-(4-fluorophenyl)-1*H*-pyrrole-3-carboxylate (**27**) (1413 mg, 6.45 mmol), and NaOH (2578 mg, 64.50 mmol) according to the procedure E. Compound **40** was obtained as a brown solid after precipitation. Yield: 841 mg (63%). ^{1}H NMR (300 MHz, DMSO-d_6) δ 11.59 (s, 1H), 11.46 (s, 1H), 7.48 (dd, *J* = 8.8, 5.7 Hz, 2H), 7.43 (t, *J* = 2.6 Hz, 1H), 7.11 (t, *J* = 9.0 Hz, 2H), 6.91 (t, *J* = 2.3 Hz, 1H). ^{13}C NMR (75 MHz, DMSO-d_6) δ 165.6, 160.8 (d, *J* = 241.8 Hz), 131.7 (d, *J* = 3.1 Hz), 130.6 (d, *J* = 7.9 Hz), 126.0, 124.1, 119.0, 114.2 (d, *J* = 21.0 Hz), 112.5. HPLC-MS $[M + H]^+$ = 206, Rt = 2.82 (97%).

4-(2-Chlorophenyl)-1*H*-pyrrole-3-carboxylic acid (41)

The title compound was prepared by reaction of methyl 4-(2-chlorophenyl)-1*H*-pyrrole-3-carboxylate (**28**) (2500 mg, 10.61 mmol), and NaOH (4243 mg, 64.10 mmol) according to the procedure E. Compound **41** was obtained as a pale yellow solid after precipitation. Yield: 1700 mg (72%). ^{1}H NMR (300 MHz, DMSO-d_6) δ 11.49 (s, 1H), 7.45 – 7.36 (m, 2H), 7.33 – 7.22 (m, 3H), 6.79 (t, *J* = 2.2 Hz, 1H). ^{13}C NMR (75 MHz, DMSO-d_6) δ 165.5, 135.2, 133.5, 132.2, 128.7, 127.8, 126.2, 124.3, 121.8, 119.3, 115.0. HPLC-MS $[M + H]^+$ = 222, Rt = 2.84 (97%).

4-(4-Chlorophenyl)-1*H*-pyrrole-3-carboxylic acid (42)

The title compound was prepared by reaction of methyl 4-(4-chlorophenyl)-1*H*-pyrrole-3-carboxylate (**29**) (1825 mg, 7.74 mmol), and NaOH (3098 mg, 77.40 mmol)

according to the procedure E. Compound **42** was obtained as a white solid after precipitation. Yield: 840 mg (49%). ^{1}H NMR (300 MHz, DMSO-d_6) δ 11.64 (s, 1H), 11.54 (s, 1H), 7.49 (d, *J* = 8.7 Hz, 2H), 7.44 (dd, *J* = 3.1, 2.2 Hz, 1H), 7.34 (d, *J* = 8.7 Hz, 2H), 6.96 (t, *J* = 2.4 Hz, 1H). ^{13}C NMR (75 MHz, DMSO-d_6) δ 165.6, 134.2, 130.5, 130.3, 127.4, 126.2, 123.9, 119.3, 112.6. HPLC-MS [M + H]$^+$ = 222, Rt = 3.00 (99 %).

4-(2-Bromophenyl)-1*H*-pyrrole-3-carboxylic acid (43)

The title compound was prepared by reaction of methyl 4-(2-bromophenyl)-1*H*-pyrrole-3-carboxylate (**30**) (500 mg, 1.79 mmol), and NaOH (713 mg, 17.90 mmol) according to the procedure E. Compound **43** was obtained as a white solid after precipitation. Yield: 269 mg (57%). ^{1}H NMR (300 MHz, DMSO-d_6) δ 11.43 (s, 2H), 7.59 (dd, *J* = 7.9, 0.8 Hz, 1H), 7.40 (dd, *J* = 3.0, 2.2 Hz, 1H), 7.34 – 7.25 (m, 2H), 7.18 (ddd, *J* = 7.9, 6.6, 2.6 Hz, 1H), 6.78 (t, *J* = 2.3 Hz, 1H). ^{13}C NMR (75 MHz, DMSO-d_6) δ 165.2, 137.2, 132.1, 131.8, 128.1, 126.7, 124.6, 124.3, 123.8, 119.2, 114.5. HPLC-MS [M + H]$^+$ = 266, Rt = 2.89 (99%).

4-(3-Bromophenyl)-1*H*-pyrrole-3-carboxylic acid (44)

The title compound was prepared by reaction of methyl 4-(3-bromophenyl)-1*H*-pyrrole-3-carboxylate (**31**) (500 mg, 1.79 mmol), and NaOH (713 mg, 17.90 mmol) according to the procedure E. Compound **44** was obtained as a pale yellow solid after precipitation. Yield: 302 mg (64%). ^{1}H NMR (300 MHz, DMSO-d_6) δ 11.67 (s, 1H), 11.55 (s, 1H), 7.68 (t, *J* = 1.7 Hz, 1H), 7.50 – 7.43 (m, 2H), 7.38 (ddd, *J* = 8.0, 2.0, 1.1 Hz, 1H), 7.25 (t, *J* = 7.9 Hz, 1H), 7.02 (t, *J* = 2.4 Hz, 1H). ^{13}C NMR (75 MHz, DMSO-d_6) δ 165.5, 137.8, 131.2, 129.6, 128.3, 127.8, 126.3, 123.5, 120.9, 119.7, 112.6. HPLC-MS [M + H]$^+$ = 266, Rt = 3.04 (99%).

4-(4-Bromophenyl)-1*H*-pyrrole-3-carboxylic acid (45)

The title compound was prepared by reaction of methyl 4-(4-bromophenyl)-1*H*-pyrrole-3-carboxylate (**32**) (500 mg, 1.79 mmol), and NaOH (713 mg, 17.90 mmol) according to the procedure E. Compound **45** was obtained as a pale yellow solid after precipitation. Yield: 343 mg (72%). ^{1}H NMR (300 MHz, DMSO-d_6) δ 11.65 (s, 1H), 11.51 (s, 1H), 7.51 – 7.37 (m, 5H), 6.96 (t, *J* = 2.3 Hz, 1H). ^{13}C NMR (75 MHz, DMSO-d_6) δ 165.6,

134.6, 130.9, 130.4, 126.3, 123.9, 119.3, 118.8, 112.6. HPLC-MS $[M + H]^+$ = 266, Rt = 3.05 (99%).

4-(*p*-Tolyl)-1*H*-pyrrole-3-carboxylic acid (46)

The title compound was prepared by reaction of methyl 4-(*p*-tolyl)-1*H*-pyrrole-3-carboxylate (**33**) (2000 mg, 9.94 mmol), and NaOH (3976 mg, 99.40 mmol) according to the procedure E. Compound **46** was obtained as a pale yellow solid after precipitation. Yield: 1044 mg (52%). ^{1}H NMR (300 MHz, DMSO-d_6) δ 11.51 (s, 1H), 11.40 (s, 1H), 7.41 (dd, *J* = 3.1, 2.2 Hz, 1H), 7.34 (d, *J* = 8.0 Hz, 2H), 7.09 (d, *J* = 7.9 Hz, 2H), 6.85 (t, *J* = 2.4 Hz, 1H), 2.29 (s, 3H). ^{13}C NMR (75 MHz, DMSO-d_6) δ 165.7, 134.6, 132.4, 128.7, 128.1, 125.9, 125.2, 118.7, 112.6, 20.7. HPLC-MS $[M + H]^+$ = 202, Rt = 2.91 (98%).

4-(4-Isopropylphenyl)-1*H*-pyrrole-3-carboxylic acid (47)

The title compound was prepared by reaction of methyl 4-(4-isopropylphenyl)-1*H*-pyrrole-3-carboxylate (**34**) (910 mg, 3.74 mmol), and NaOH (1496 mg, 37.40 mmol) according to the procedure E. Compound **47** was obtained as a white solid after precipitation. Yield: 552 mg (64%). ^{1}H NMR (300 MHz, DMSO-d_6) δ 11.52 (s, 1H), 11.43 (s, 1H), 7.41 (t, *J* = 2.4 Hz, 1H), 7.37 (d, *J* = 8.2 Hz, 2H), 7.15 (d, *J* = 8.1 Hz, 2H), 6.86 (t, *J* = 2.3 Hz, 1H), 2.87 (hept, *J* = 6.9 Hz, 1H), 1.21 (d, *J* = 6.9 Hz, 6H). ^{13}C NMR (75 MHz, DMSO-d_6) δ 165.7, 145.7, 132.8, 128.8, 125.9, 125.4, 125.2, 118.8, 112.5, 33.1, 24.0. HPLC-MS $[M + H]^+$ = 230, Rt = 3.25 (98%).

4-(4-(*Tert*-butyl)phenyl)-1*H*-pyrrole-3-carboxylic acid (48)

The title compound was prepared by reaction of methyl 4-(4-(*tert*-butyl)phenyl)-1*H*-pyrrole-3-carboxylate (**35**) (1000 mg, 3.89 mmol), and NaOH (1554 mg, 38.90 mmol) according to the procedure E. Compound **48** was obtained as a white solid after precipitation. Yield: 550 mg (58%). ^{1}H NMR (300 MHz, DMSO-d_6) δ 11.52 (s, 1H), 11.41 (s, 1H), 7.42 (t, *J* = 2.5 Hz, 1H), 7.39 (d, *J* = 8.5 Hz, 2H), 7.30 (d, *J* = 8.5 Hz, 2H), 6.86 (t, *J* = 2.3 Hz, 1H), 1.30 (s, 9H). ^{13}C NMR (75 MHz, DMSO-d_6) δ 165.7, 147.9, 132.4, 128.5, 125.9, 125.1, 124.2, 118.8, 112.6, 34.1, 31.2. HPLC-MS $[M + H]^+$ = 244, Rt = 3.37 (99%).

4-(2,4-Dimethylphenyl)-1*H*-pyrrole-3-carboxylic acid (49)

The title compound was prepared by reaction of methyl 4-(2,4-dimethylphenyl)-1*H*-pyrrole-3-carboxylate (**36**) (790 mg, 3.45 mmol), and NaOH (1378 mg, 34.50 mmol) according to the procedure E. Compound **49** was obtained as a yellow solid after precipitation. Yield: 452 mg (61%). ^{1}H NMR (300 MHz, DMSO-d_6) δ 11.35 (s, 1H), 7.40 (t, *J* = 2.5 Hz, 1H), 7.00 – 6.94 (m, 2H), 6.89 (d, *J* = 7.8 Hz, 1H), 6.64 (t, *J* = 2.3 Hz, 1H), 2.27 (s, 3H), 2.08 (s, 3H). ^{13}C NMR (75 MHz, DMSO-d_6) δ 165.5, 136.5, 135.1, 132.9, 130.3, 129.8, 125.3, 124.6, 124.2, 118.6, 114.2, 20.7, 20.0. HPLC-MS $[M + H]^+$ = 216, Rt = 3.04 (78%).

4-(4-Nitrophenyl)-1*H*-pyrrole-3-carboxylic acid (50)

The title compound was prepared by reaction of methyl 4-(4-nitrophenyl)-1*H*-pyrrole-3-carboxylate (**37**) (500 mg, 2.03 mmol), and NaOH (812 mg, 20.30 mmol) according to the procedure E. Compound **50** was obtained as a yellow solid after precipitation. Yield: 128 mg (27%). ^{1}H NMR (300 MHz, DMSO-d_6) δ 11.84 (s, 1H), 11.71 (s, 1H), 8.16 (d, *J* = 9.1 Hz, 2H), 7.77 (d, *J* = 9.1 Hz, 2H), 7.52 (dd, *J* = 3.0, 2.2 Hz, 1H), 7.19 (t, *J* = 2.4 Hz, 1H). ^{13}C NMR (75 MHz, DMSO-d_6) δ 165.5, 145.2, 142.6, 129.4, 127.1, 123.0, 122.9, 121.0, 112.9. HPLC-MS $[M + H]^+$ = 233, Rt = 2.82 (99%).

4-(Pyridin-3-yl)-1*H*-pyrrole-3-carboxylic acid (51)

The title compound was prepared by reaction of methyl 4-(pyridin-3-yl)-1*H*-pyrrole-3-carboxylate (**38**) (260 mg, 1.29 mmol), and NaOH (516 mg, 12.90 mmol) according to the procedure E. Compound **51** was obtained as a brown solid after precipitation. Yield: 113 mg (47%). ^{1}H NMR (300 MHz, DMSO-d_6) δ 12.17 (s, 1H), 9.06 (d, *J* = 2.0 Hz, 1H), 8.76 – 8.64 (m, 2H), 7.99 (dd, *J* = 8.1, 5.6 Hz, 1H), 7.57 (dd, *J* = 3.0, 2.2 Hz, 1H), 7.38 (t, *J* = 2.4 Hz, 1H). ^{13}C NMR (75 MHz, DMSO-d_6) δ 165.5, 144.5, 140.4, 138.3, 134.5, 127.3, 126.2, 121.8, 118.1, 112.7. HPLC-MS $[M + H]^+$ = 189, Rt = 1.04 (96%).

4-(Pyridin-4-yl)-1*H*-pyrrole-3-carboxylic acid (52)

The title compound was prepared by reaction of methyl 4-(pyridin-4-yl)-1*H*-pyrrole-3-carboxylate (**39**) (635 mg, 3.14 mmol), and NaOH (1256 mg, 31.40 mmol) according to the procedure E. Compound **52** was obtained as a brown solid after precipitation. Yield: 112 mg (19%). ^{1}H NMR (300 MHz, DMSO-d_6) δ 12.44 (s, 1H), 8.74 (d, *J* = 7.0 Hz, 2H), 8.29 (d, *J* = 6.9 Hz, 2H), 7.73 (dd, *J* = 2.9, 2.2 Hz, 1H), 7.63 (dd, *J* = 2.9, 2.1 Hz, 1H). ^{13}C

NMR (75 MHz, DMSO-d_6) δ 165.4, 152.0, 140.2, 129.0, 125.2, 124.5, 119.6, 113.6. HPLC-MS $[M + H]^+$ = 189, Rt = 1.09 (97%).

4-(4-Chlorophenyl)-1-methyl-1*H*-pyrrole-3-carboxylic acid (82)

The title compound was prepared by reaction of methyl 4-(4-chlorophenyl)-1-methyl-1*H*-pyrrole-3-carboxylate (**81**) (200 mg, 0.80 mmol), and NaOH (320 mg, 8.00 mmol) according to the procedure E. Compound **82** was obtained as a white solid after precipitation. Yield: 170 mg (92%). ^{1}H NMR (300 MHz, DMSO-d_6) δ 11.65 (s, 1H), 7.46 (d, *J* = 8.7 Hz, 2H), 7.43 (d, *J* = 2.5 Hz, 1H), 7.34 (d, *J* = 8.7 Hz, 2H), 6.93 (d, *J* = 2.4 Hz, 1H), 3.66 (s, 3H). ^{13}C NMR (75 MHz, DMSO-d_6) δ 165.2, 133.8, 130.5, 130.4, 129.7, 127.5, 124.3, 123.1, 112.3, 36.1. HPLC-MS $[M + H]^+$ = 236, Rt = 3.25 (99%).

4-(3,4-Dichlorophenyl)-1-methyl-1*H*-pyrrole-3-carboxylic acid (101)

The title compound was prepared by reaction of methyl 4-(3,4-dichlorophenyl)-1-methyl-1*H*-pyrrole-3-carboxylate (**100**) (300 mg, 1.06 mmol), and NaOH (422 mg, 10.60 mmol) according to the procedure E. Compound **101** was obtained as a white solid after precipitation. Yield: 266 mg (93%). ^{1}H NMR (300 MHz, DMSO-d_6) δ 11.76 (s, 1H), 7.72 (d, *J* = 2.0 Hz, 1H), 7.54 (d, *J* = 8.3 Hz, 1H), 7.48 – 7.42 (m, 2H), 7.05 (d, *J* = 2.5 Hz, 1H), 3.66 (s, 3H). ^{13}C NMR (75 MHz, DMSO-d_6) δ 165.1, 135.7, 130.2, 130.1, 130.0, 129.7, 128.8, 128.2, 123.8, 122.8, 112.3, 36.1. HPLC-MS $[M + H]^+$ = 270, Rt = 3.46 (95%).

Compounds prepared following the general procedure F (derivatives 71-75)

4-(4-Methoxyphenyl)-1*H*-pyrrole-3-carboxylic acid (71)

The title compound was prepared by reaction of 4-methoxybenzaldehyde (349 µL, 2.87 mmol), diethyl malonate (654 µL, 4.30 mmol), triethylamine (400 µL, 2.87 mmol), pyrrolidine (72 µL, 0.86 mmol), TosMIC (605 mg, 3.16 mmol) and NaOH (574 mg, 14,35 mmol/ 1148 mg, 28.70 mmol) according to the procedure F. Compound **71** was obtained as a beige solid after precipitation. Yield: 160 mg (26%). ^{1}H NMR (300 MHz, DMSO-d_6) δ 11.49 (s, 1H), 11.37 (s, 1H), 7.44 – 7.32 (m, 3H), 6.89 – 6.79 (m, 3H), 3.75 (s, 3H).^{13}C NMR (75 MHz, DMSO-d_6) δ 166.2, 158.0, 130.4, 128.2, 126.3, 125.4, 118.9, 113.4, 113.0, 55.5. HPLC-MS [M + H]$^+$ = 218, Rt = 2.92 (99%).

4-(4-(Dimethylamino)phenyl)-1*H*-pyrrole-3-carboxylic acid (72)

The title compound was prepared by reaction of 4-(dimethylamino)benzaldehyde (325 mg, 2.18 mmol), diethyl malonate (497 µL, 3.27 mmol), triethylamine (304 µL, 2.18 mmol), pyrrolidine (55 µL, 0.66 mmol), TosMIC (468 mg, 2.40 mmol) and NaOH (436 mg, 10.90 mmol (872 mg, 21.80 mmol) according to the procedure F. Compound **72** was obtained as a grey solid after precipitation. Yield: 126 mg (25%). ^{1}H NMR (300 MHz, DMSO-d_6) δ 11.42 (s, 1H), 11.32 (s, 1H), 7.37 (dd, J = 3.1, 2.2 Hz, 1H), 7.33 – 7.27 (m, 2H), 6.77 (t, J = 2.4 Hz, 1H), 6.73 – 6.62 (m, 2H), 2.88 (s, 6H). ^{13}C NMR (75 MHz, DMSO-d_6) δ 165.8, 148.8, 129.4, 125.6, 125.5, 123.6, 117.8, 112.4, 111.9, 40.4. HPLC-MS [M + H]$^+$ = 231, Rt = 1.93 (96%).

4-(4-Morpholinophenyl)-1*H*-pyrrole-3-carboxylic acid (73)

The title compound was prepared by reaction of 4-morpholinobenzaldehyde (400 mg, 2.09 mmol), diethyl malonate (476 µL, 3.14 mmol), triethylamine (291 µL, 2.09 mmol) , pyrrolidine (53 µL, 0.63 mmol), TosMIC (449 mg, 2.30 mmol) and NaOH (418 mg, 10.45 mmol (836 mg, 20.90 mmol) according to the procedure F. Compound **73** was obtained as a beige solid after precipitation. Yield: 153 mg (27%). ^{1}H NMR (300 MHz, DMSO-d_6) δ 11.37 (s, 1H), 7.39 (dd, J = 3.1, 2.2 Hz, 1H), 7.37 – 7.30 (m, 2H), 6.92 – 6.84 (m, 2H), 6.82 (t, J = 2.3 Hz, 1H), 3.80 – 3.71 (m, 4H), 3.16 – 3.07 (m, 4H). ^{13}C NMR (75 MHz, DMSO-d_6) δ 166.3, 149.7, 129.9, 126.9, 126.2, 125.6, 118.6, 114.9, 113.0, 66.6, 49.2. HPLC-MS [M + H]$^+$ = 273, Rt = 2.49 (97%).

4-(Quinolin-4-yl)-1*H*-pyrrole-3-carboxylic acid (74)

The title compound was prepared by reaction of quinoline-4-carbaldehyde (500 mg, 3.18 mmol), diethyl malonate (579 µL, 3.82 mmol), triethylamine (443 µL, 3.18 mmol) , pyrrolidine (80 µL, 0.96 mmol), TosMIC (683 mg, 3.50 mmol) and NaOH (636 mg, 15.9 mmol (1272 mg, 31.80 mmol) according to the procedure F. Compound **74** was obtained as a dark brown solid after precipitation. Yield: 344 mg (45%). ^{1}H NMR (300 MHz, DMSO-d_6) δ 11.68 (s, 1H), 8.99 (d, J = 2.2 Hz, 1H), 8.34 (d, J = 2.2 Hz, 1H), 8.03 – 7.88 (m, 2H), 7.70 (ddd, J = 8.4, 6.8, 1.5 Hz, 1H), 7.61 – 7.50 (m, 2H), 7.17 (t, J = 2.3 Hz, 1H). ^{13}C NMR (75 MHz, DMSO-d_6) δ 166.2, 152.5, 146.3, 133.9, 129.2, 129.0, 129.0, 128.4, 128.1, 126.9, 126.8, 122.1, 120.6, 113.6. HPLC-MS [M + H]$^+$ = 239, Rt = 2.21 (96%).

4-(Quinolin-3-yl)-1*H*-pyrrole-3-carboxylic acid (75)

The title compound was prepared by reaction of quinoline-3-carbaldehyde (500 mg, 3.18 mmol), diethyl malonate (579 µL, 3.82 mmol), triethylamine (443 µL, 3.18 mmol) , pyrrolidine (80 µL, 0.96 mmol), TosMIC (683 mg, 3.50 mmol) and NaOH (636 mg, 15.9 mmol (1272 mg, 31.80 mmol) according to the procedure F. Compound **75** was obtained as a dark brown solid after precipitation. Yield: 185 mg (24%). ^{1}H NMR (300 MHz, DMSO-d_6) δ 12.23 – 12.17 (m, 1H), 9.17 (d, J = 5.6 Hz, 1H), 8.39 (dt, J = 8.4, 1.0 Hz, 1H), 8.18 (d, J = 1.3 Hz, 1H), 8.09 (ddd, J = 8.5, 6.9, 1.4 Hz, 1H), 7.92 – 7.81 (m, 2H), 7.70 (dd, J = 3.0, 2.1 Hz, 1H), 7.31 (dd, J = 2.7, 2.1 Hz, 1H). ^{13}C NMR (75 MHz, DMSO-d_6) δ 164.8, 152.6, 143.5, 138.6, 133.4, 128.9, 127.8, 127.6, 126.8, 123.0, 122.6, 121.5, 118.5, 115.0. HPLC-MS [M + H]$^+$ = 239, Rt = 1.91 (99%).

Compounds prepared following the general procedure G (derivatives 86-90)

4-Phenyl-1*H*-pyrazole-3-carboxylic acid (86)

The title compound was prepared by reaction of (*E*)-(2-nitrovinyl)benzene (150 mg, 1.00 mmol), ethyl diazoacetate (0.5 mL, 4.00 mmol), and TEA (28 µL, 0.20 mmol) according to the procedure G. Compound **86** was obtained as a brown solid after precipitation. Yield: 124 mg (66%). ^{1}H NMR (300 MHz, DMSO-d_6) δ 7.87 (s, 1H), 7.56 – 7.50 (m, 2H), 7.36 (t, *J* = 7.3 Hz, 2H), 7.28 (t, *J* = 7.2 Hz, 1H). ^{13}C NMR (75 MHz, DMSO-d_6) δ 162.3, 132.1, 129.0, 127.9, 126.8, 124.2. HPLC-MS $[M + H]^+$ = 189, Rt = 2.59 (98%).

4-(4-Fluorophenyl)-1*H*-pyrazole-3-carboxylic acid (87)

The title compound was prepared by reaction of (*E*)-1-fluoro-4-(2-nitrovinyl)benzene (1003 mg, 6.00 mmol), ethyl diazoacetate (19.2 mL, 24.00 mmol), and TEA (167 µL, 1.20 mmol) according to the procedure G. Compound **87** was obtained as a brown solid after precipitation. Yield: 817 mg (65%). ^{1}H NMR (300 MHz, DMSO-d_6) δ 13.22 (s, 2H), 7.87 (s, 1H), 7.57 (dd, *J* = 8.9, 5.6 Hz, 2H), 7.19 (t, *J* = 9.0 Hz, 2H). ^{13}C NMR (75 MHz, DMSO-d_6) δ 162.2, 161.3 (d, *J* = 243.5 Hz), 130.9 (d, *J* = 8.1 Hz), 128.5 (d, *J* = 3.1 Hz), 123.2, 114.7 (d, *J* = 21.3 Hz). HPLC-MS $[M + H]^+$ = 207, Rt = 2.66 (95%).

4-(*p*-Tolyl)-1*H*-pyrazole-3-carboxylic acid (88)

The title compound was prepared by reaction of (*E*)-1-methyl-4-(2-nitrovinyl)benzene (979 mg, 6.00 mmol), ethyl diazoacetate (19.2 mL, 24.00 mmol), and TEA (167 µL, 1.20 mmol) according to the procedure G. Compound **88** was obtained as a brown solid after precipitation. Yield: 594 mg (49%). ^{1}H NMR (300 MHz, DMSO-d_6) δ 13.25 (s, 2H), 7.82 (s, 1H), 7.42 (d, *J* = 8.1 Hz, 2H), 7.16 (d, *J* = 7.9 Hz, 2H), 2.31 (s, 3H). ^{13}C NMR (75 MHz, DMSO-d_6) δ 162.3, 136.0, 129.2, 128.8, 128.5, 124.2, 20.8. HPLC-MS $[M + H]^+$ = 203, Rt = 2.77 (99%).

4-(4-Methoxyphenyl)-1*H*-pyrazole-3-carboxylic acid (89)

The title compound was prepared by reaction of (*E*)-1-methoxy-4-(2-nitrovinyl)benzene (1.08 g, 6.00 mmol), ethyl diazoacetate (19.2 mL, 24.00 mmol), and TEA (167 µL, 1.20 mmol) according to the procedure G. Compound **89** was obtained as a brown solid after precipitation. Yield: 720 mg (55%). ^{1}H NMR (300 MHz, DMSO-d_6) δ

7.80 (s, 1H), 7.48 (d, *J* = 8.8 Hz, 2H), 6.93 (d, *J* = 8.9 Hz, 2H), 3.77 (s, 3H). ^{13}C NMR (75 MHz, DMSO-d_6) δ 162.3, 158.3, 130.1, 124.4, 124.1, 113.4, 55.1. HPLC-MS $[M + H]^+$ = 219, Rt = 2.60 (90%).

4-(Furan-2-yl)-1*H*-pyrazole-3-carboxylic acid (90)

The title compound was prepared by reaction of (*E*)-2-(2-nitrovinyl)furan (835 mg, 6.00 mmol), ethyl diazoacetate (19.2 mL, 24.00 mmol), and TEA (167 µL, 1.20 mmol) according to the procedure G. Compound **90** was obtained as a brown solid after precipitation. Yield: 178 mg (17%). ^{1}H NMR (300 MHz, DMSO-d_6) δ 13.47 (s, 2H), 8.01 (s, 1H), 7.66 (d, *J* = 1.7 Hz, 1H), 7.06 (d, *J* = 3.1 Hz, 1H), 6.53 (dd, *J* = 3.3, 1.8 Hz, 1H). ^{13}C NMR (75 MHz, DMSO-d_6) δ 161.9, 146.4, 141.8, 115.0, 111.6, 108.8. HPLC-MS $[M + H]^+$ = 179, Rt = 2.45 (98%).

^{1}H and ^{13}C NMR spectra and HPLC chromatogram of compound 53

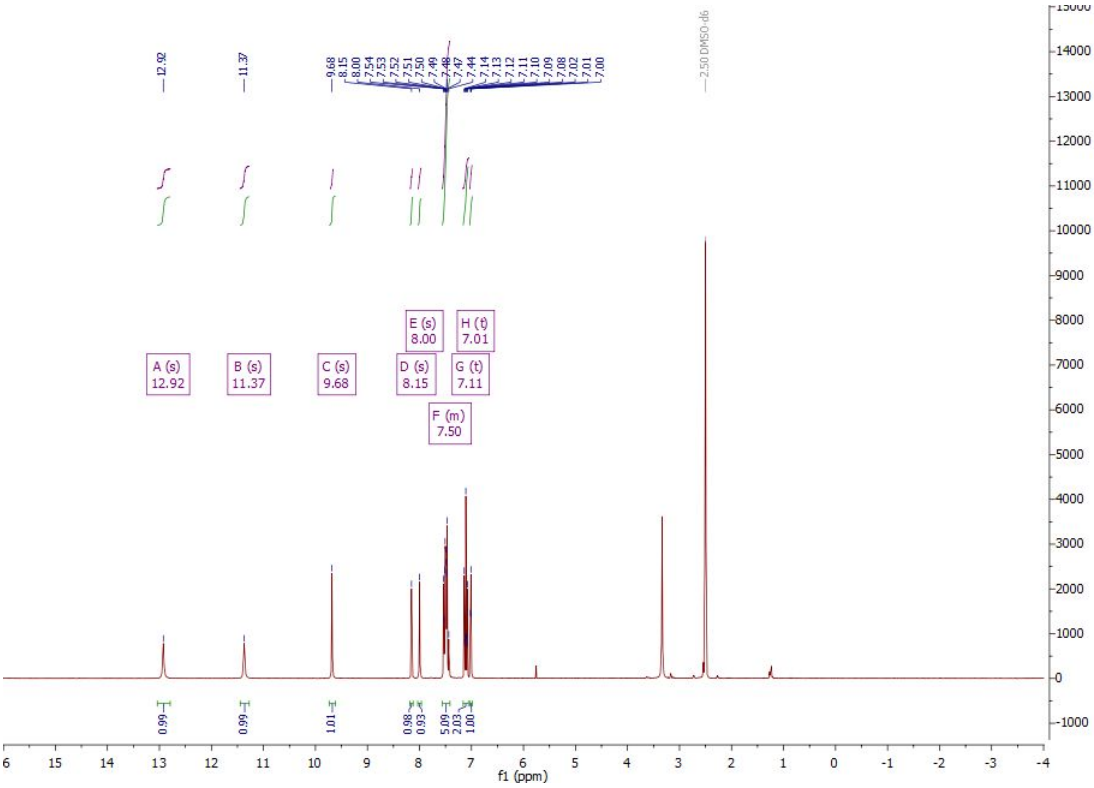

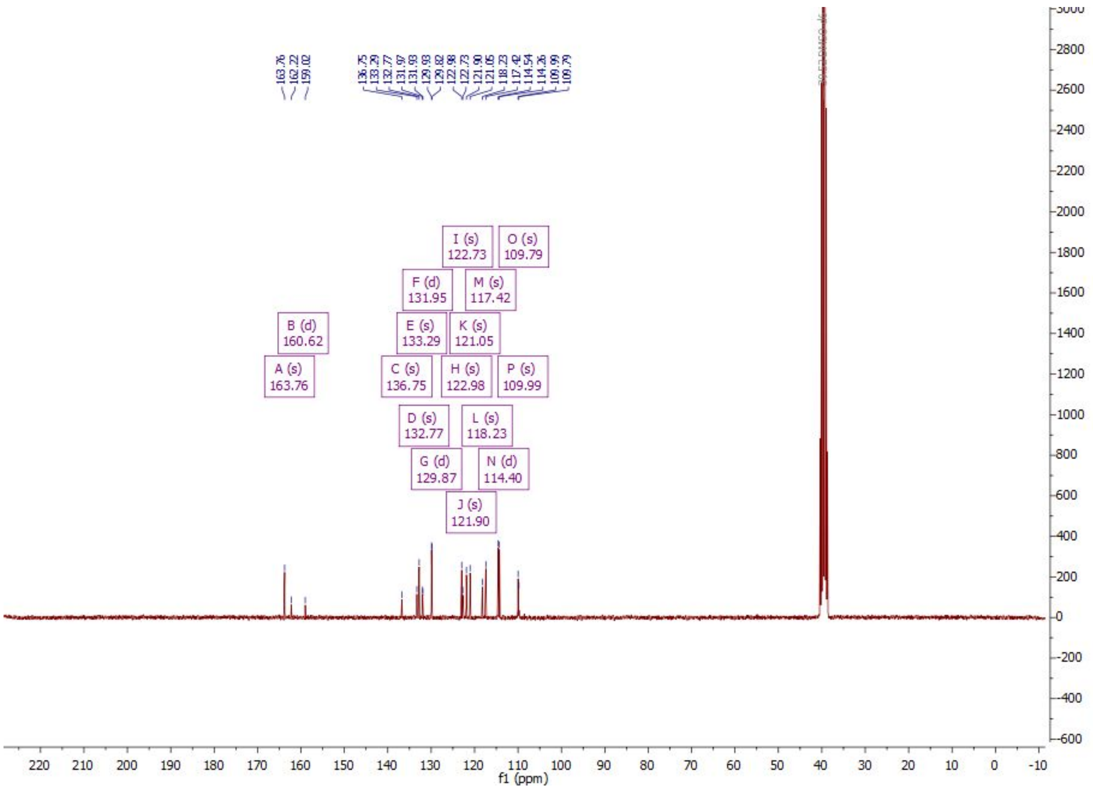

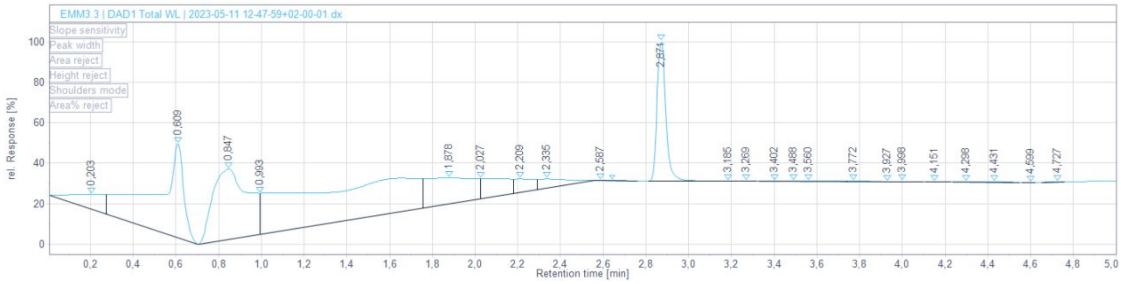
EMM3.3 | DAD1 Total WL | 2023-05-11 12-47-59+02-00-01.dx
Slope sensitivity
Peak width
Area reject
Height reject
Shoulders mode
Area% reject
rel. Response [%]
Retention time [min]
0,203
0,609
0,847
0,993
1,878
2,027
2,209
2,335
2,587
2,871
3,185
3,269
3,402
3,488
3,560
3,772
3,927
3,998
4,151
4,298
4,431
4,599
4,727

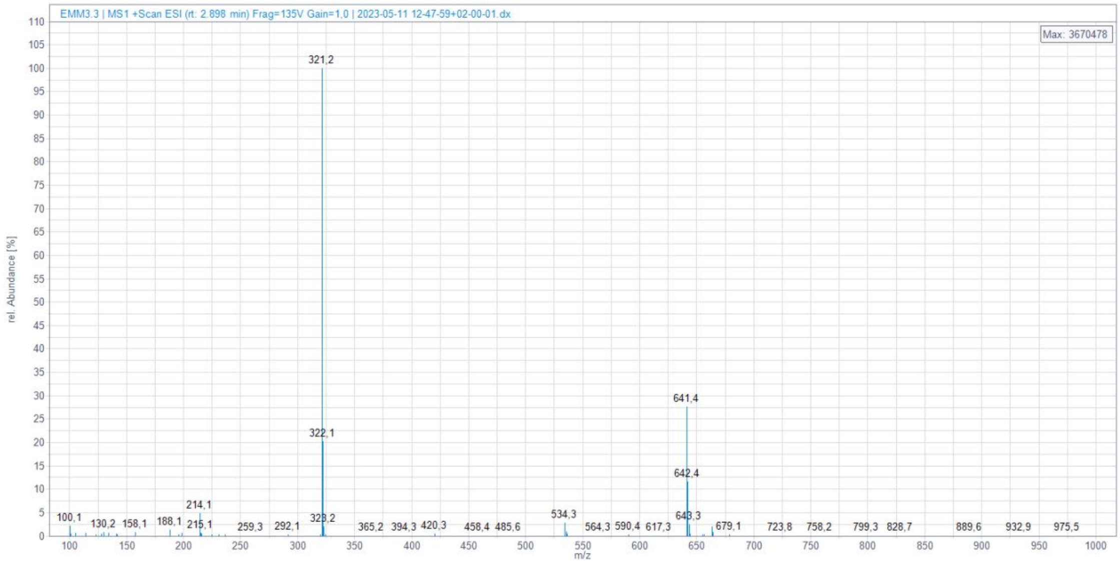
EMM3.3 | MS1 +Scan ESI (rt: 2.898 min) Frag=135V Gain=1,0 | 2023-05-11 12-47-59+02-00-01.dx
Max: 3670478
rel. Abundance [%]
m/z
321,2
322,1
323,2
641,4
642,4
643,3
100,1
130,2
158,1
188,1
214,1
215,1
259,3
292,1
365,2
394,3
420,3
458,4
485,6
534,3
564,3
590,4
617,3
679,1
723,8
758,2
799,3
828,7
889,6
932,9
975,5

^{1}H and ^{13}C NMR spectra and HPLC chromatogram of compound 55

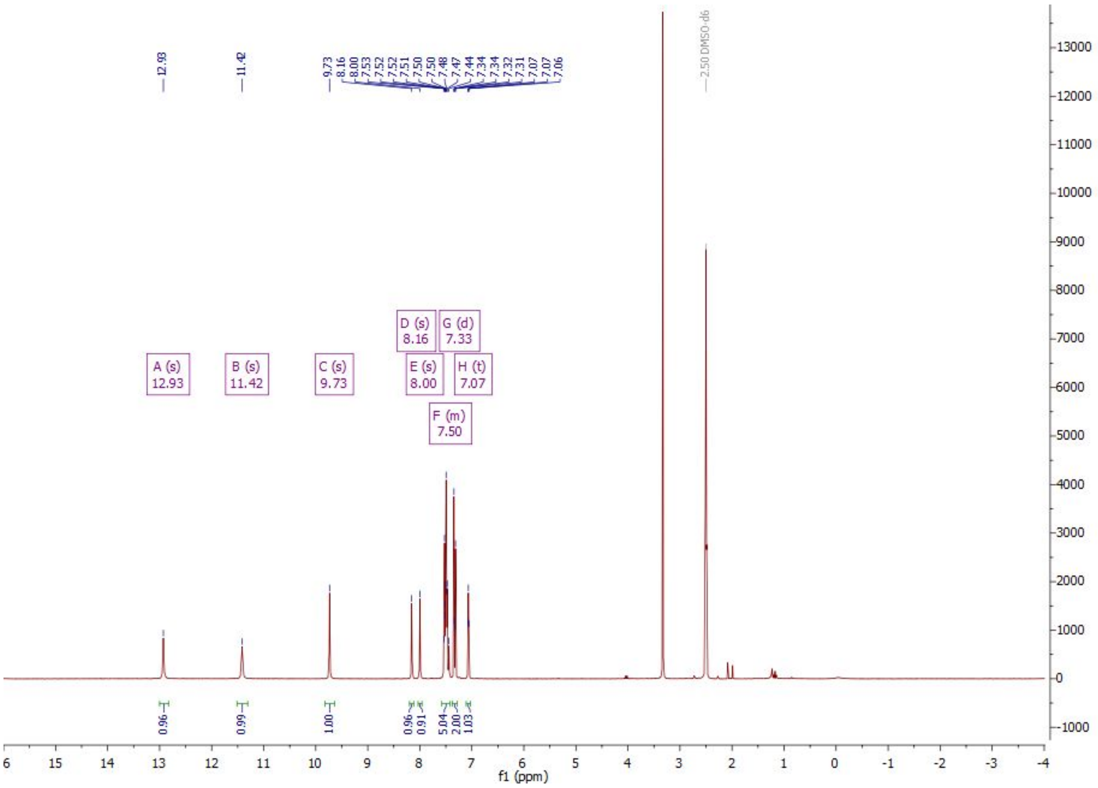

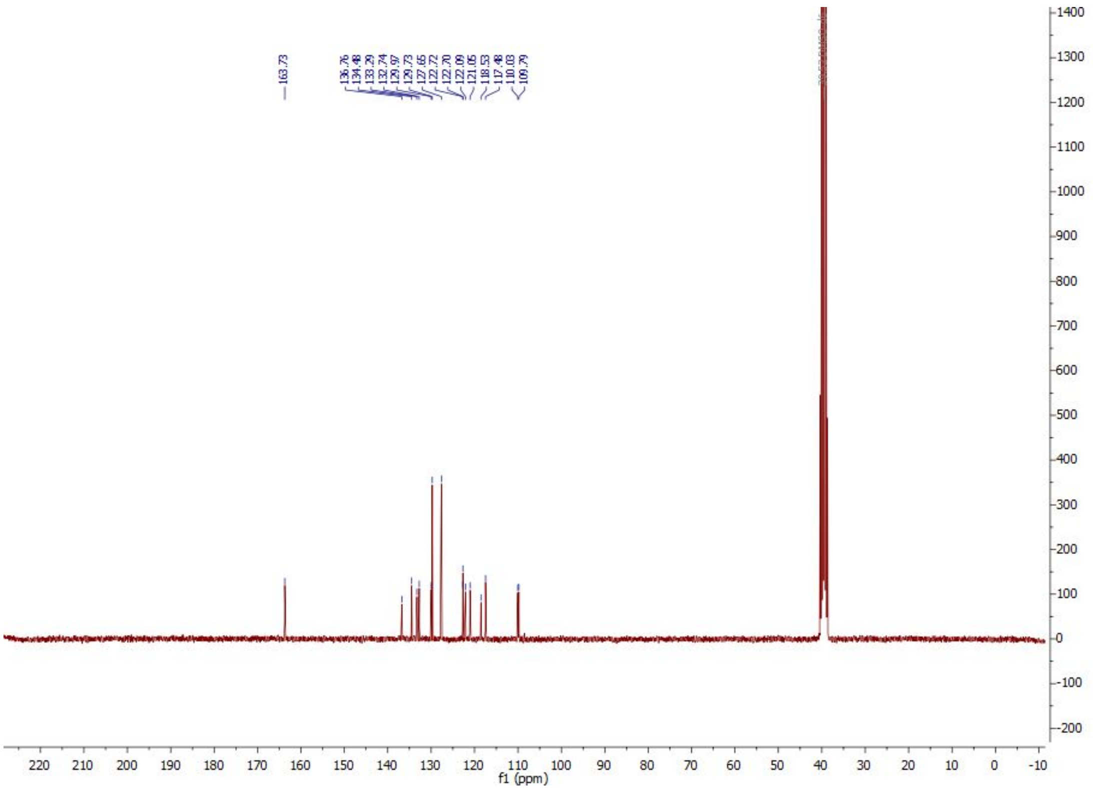

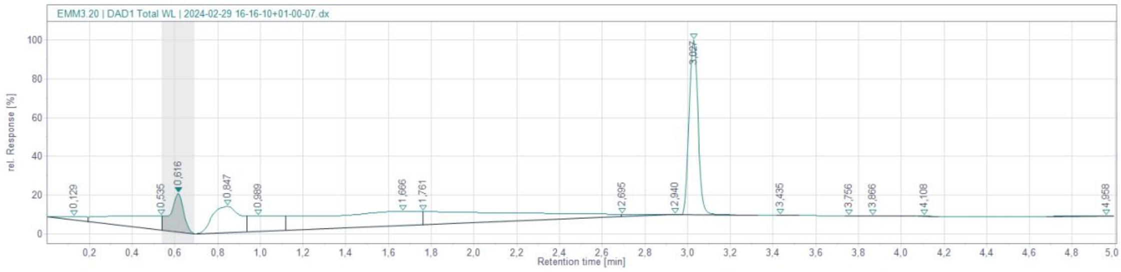
EMM3.20 | DAD1 Total WL | 2024-02-29 16-16-10+01-00-07.dx
rel. Response [%]
Retention time [min]
0,129
0,535
0,616
0,847
0,989
1,666
1,761
2,695
2,940
3,027
3,435
3,756
3,866
4,108
4,958

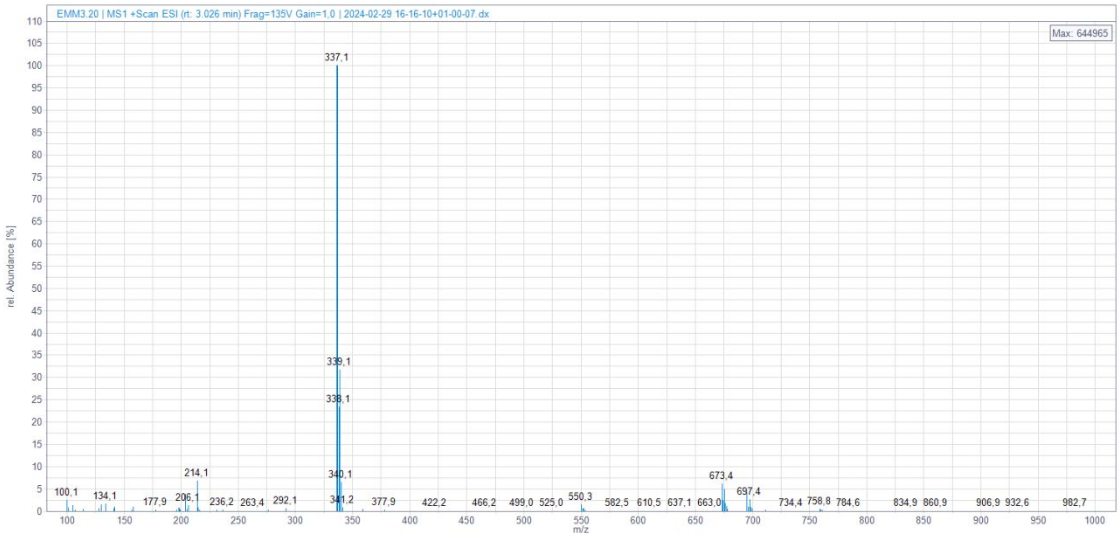
EMM3.20 | MS1 +Scan ESI (rt: 3.026 min) Frag=135V Gain=1,0 | 2024-02-29 16-16-10+01-00-07.dx
Max: 644965
rel. Abundance [%]
m/z
337,1
339,1
338,1
340,1
341,2
214,1
206,1
100,1
134,1
177,9
236,2
263,4
292,1
377,9
422,2
466,2
499,0
525,0
550,3
582,5
610,5
637,1
663,0
673,4
697,4
734,4
758,8
784,6
834,9
860,9
906,9
932,6
982,7

^{1}H and ^{13}C NMR spectra and HPLC chromatogram of compound 83

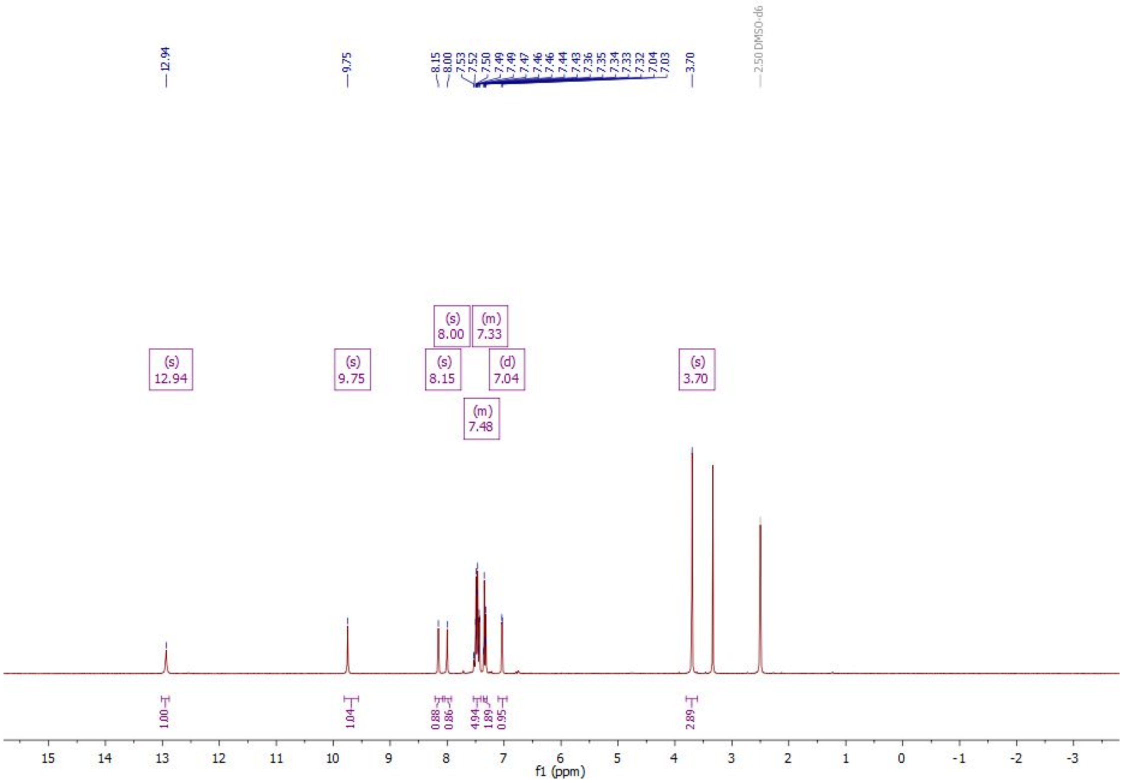

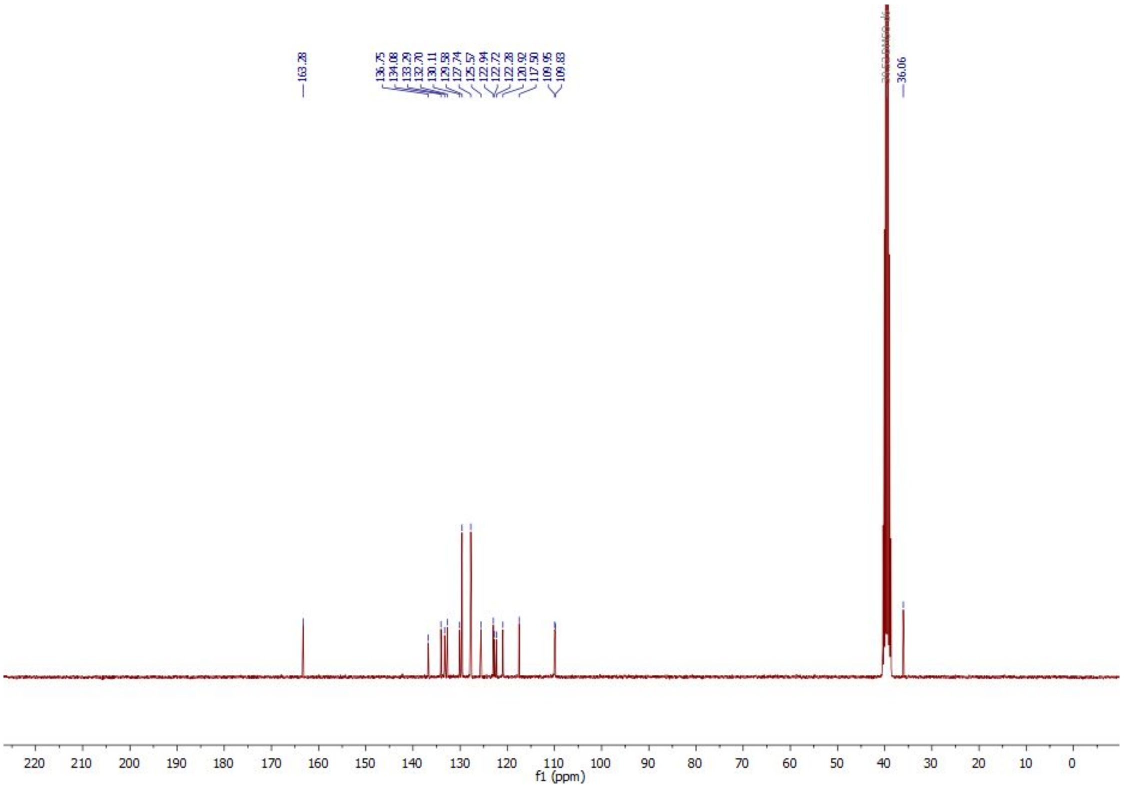

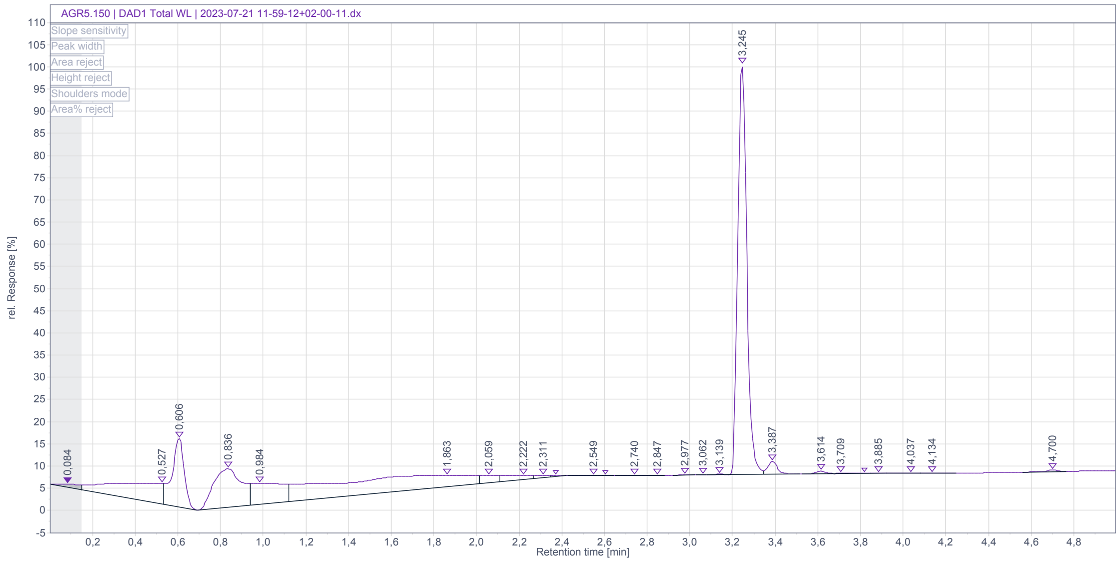
AGR5.150 | DAD1 Total WL | 2023-07-21 11-59-12+02-00-11.dx
Slope sensitivity
Peak width
Area reject
Height reject
Shoulders mode
Area% reject
rel. Response [%]
Retention time [min]
0,084
0,527
0,606
0,836
0,984
1,863
2,059
2,222
2,311
2,549
2,740
2,847
2,977
3,062
3,139
3,245
3,387
3,614
3,709
3,885
4,037
4,134
4,700

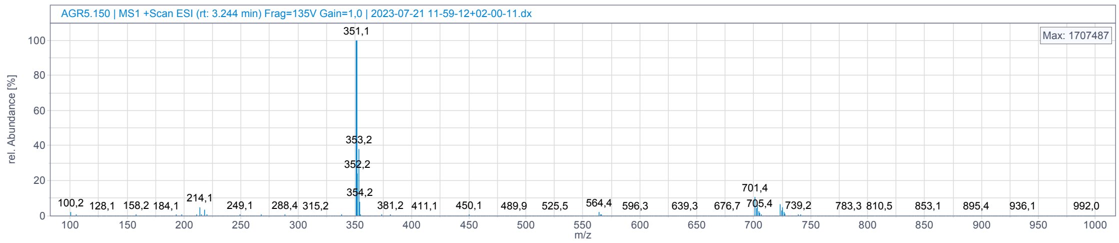
AGR5.150 | MS1 +Scan ESI (rt: 3.244 min) Frag=135V Gain=1,0 | 2023-07-21 11-59-12+02-00-11.dx
Max: 1707487
rel. Abundance [%]
m/z
351,1
353,2
352,2
354,2
100,2
128,1
158,2
184,1
214,1
249,1
288,4
315,2
381,2
411,1
450,1
489,9
525,5
564,4
596,3
639,3
676,7
701,4
705,4
739,2
783,3
810,5
853,1
895,4
936,1
992,0

^{1}H and ^{13}C NMR spectra and HPLC chromatogram of compound 102

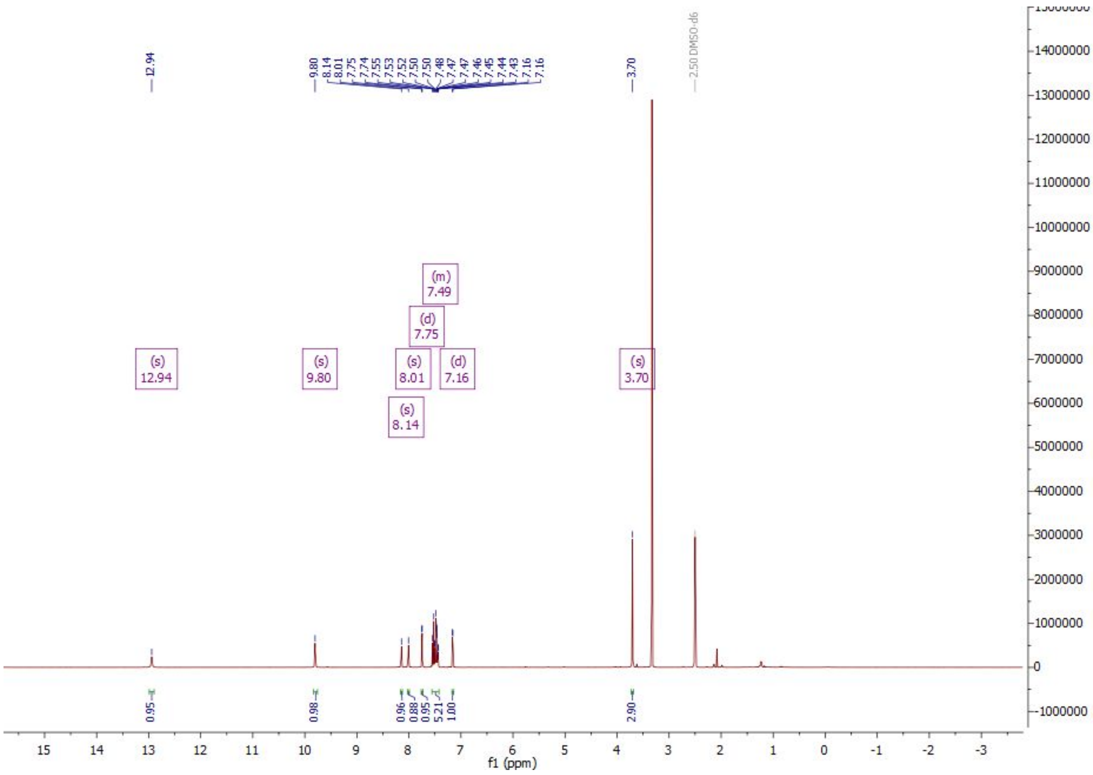

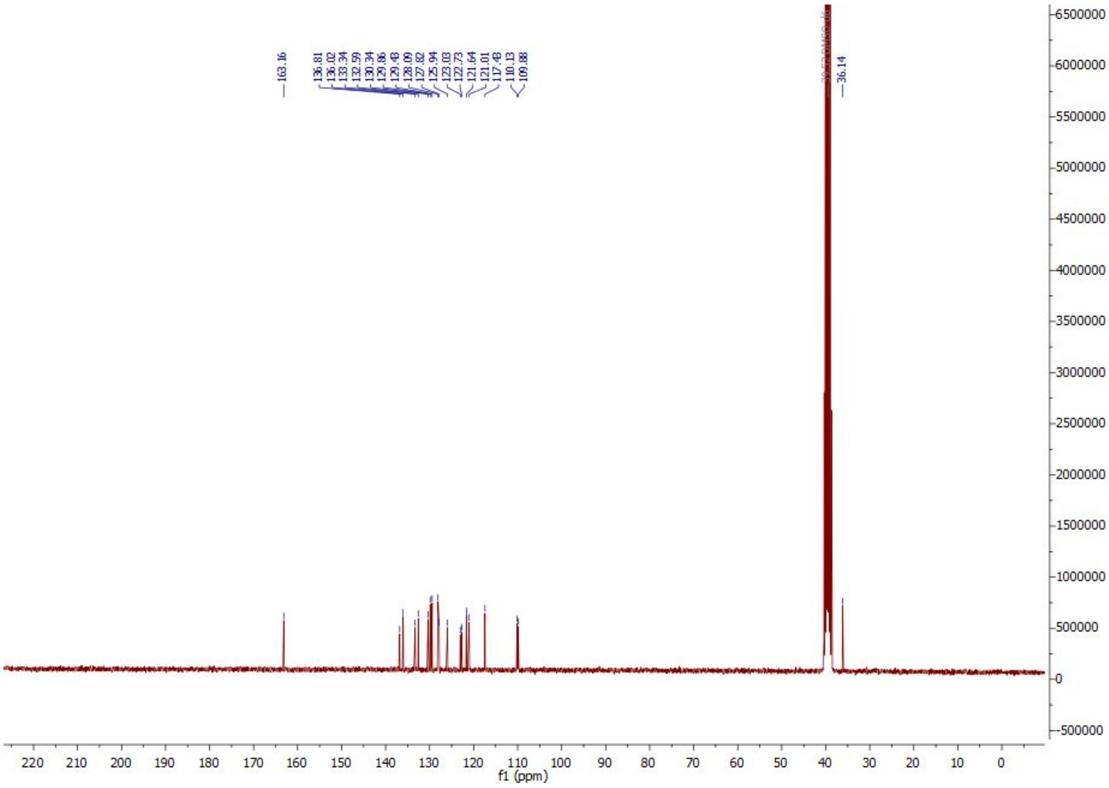

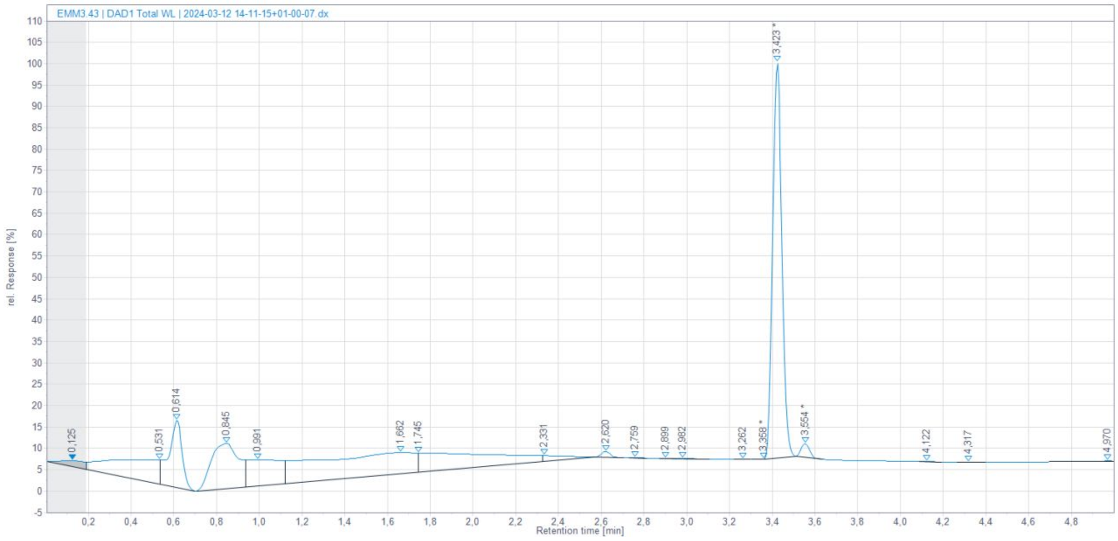
EMM3.43 | DAD1 Total WL | 2024-03-12 14-11-15+01-00-07.dx
rel. Response [%]
Retention time [min]
3,423 *
0,614
0,845
3,554 *

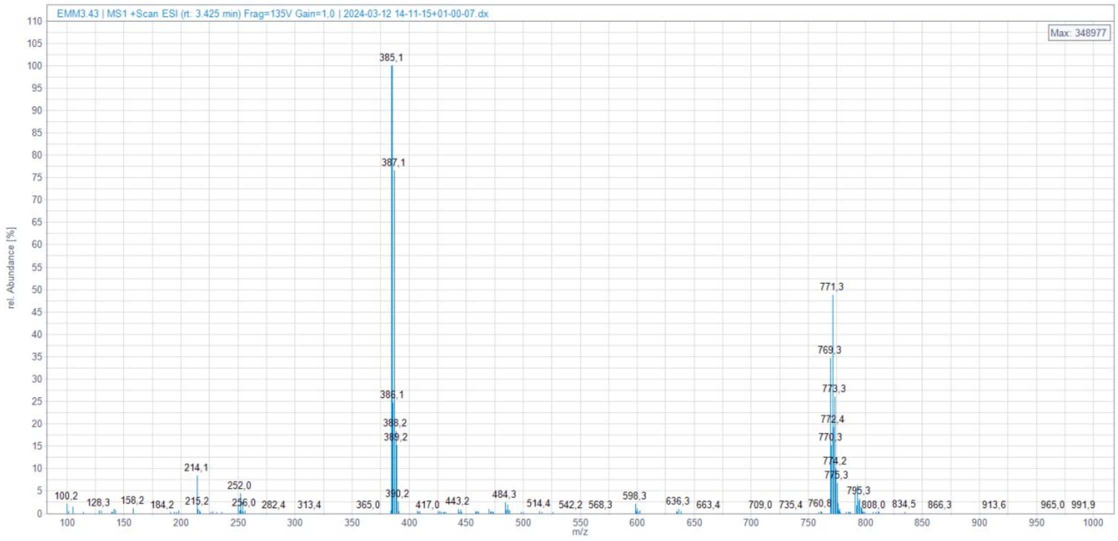
EMM3.43 | MS1 +Scan ESI (rt: 3.425 min) Frag=135V Gain=1,0 | 2024-03-12 14-11-15+01-00-07.dx
Max: 348977
rel. Abundance [%]
m/z
385,1
387,1
771,3
769,3
773,3
214,1
252,0
